# Health inequalities in disease burden of dementia and early-onset dementia: findings from the Global Burden of Disease 2021 study

**DOI:** 10.1186/s41256-025-00417-x

**Published:** 2025-05-19

**Authors:** Min Du, Lu Gram, Fude Yang, Donghua Mi, Hongguang Chen, Chao Song, Min Liu, Jue Liu

**Affiliations:** 1 Department of Epidemiology and Biostatistics, School of Public Health, Peking University, No.38, Xueyuan Road, Haidian District, Beijing, 100191 China; 2 Institute for Global Health, University College London, Gower Street, London, WC1E 6BT UK; 3 Peking University HuiLongGuan Clinical Medical School, Beijing Huilongguan Hospital, No. 29, Huilongnan Road, Changping District, Beijing, 102208 China; 4 China National Clinical Research Center for Neurological Diseases, Beijing Tiantan Hospital, Capital Medical University, No.6 Tiantan Xili, Dongcheng District, Beijing, 100050 China; 5 Peking University Institute of Mental Health, NHC Key Laboratory of Mental Health, No. 51 Huayuanbei Road, Haidian District, Beijing, 100191 China; 6 Kunming University of Science and Technology, Anning First People’s Hospital, No. 727 South Jingming Road, Chenggong District, Kunming, Yunnan Province China; 7 Institute for Global Health and Development, Peking University, No.5, Yiheyuan Road, Haidian District, Beijing, 100871 China

**Keywords:** Dementia, Health disparities, Incidence, Mortality, Early-onset

## Abstract

**Background:**

Given the increasing aging global population and advancements in dementia action plans, the latest disparities in the dementia burden may evolve. This study aimed to analyze and compare temporal trends at regional, national, and sociodemographic levels to provide evidence for public health planning and resource prioritization.

**Methods:**

The age-standardized prevalence rate (ASPR), age-standardized incidence rate (ASIR), age-standardized mortality rate (ASMR), and age-standardized disability-adjusted life years (DALYs) rate (ASDR) were derived from the Global Burden of Disease 2021 study. Estimated annual percentage changes (EAPCs) were calculated to quantify their temporal trends. Correlations between EAPC and the human resources for health (HRH) were assessed using Pearson correlation analysis.

**Results:**

Although the ASPR and ASIR remained stable or decreased globally, they both greatly increased in East Asia (ASPR: EAPC = 0.43; 95% CI 0.35, 0.51; ASIR: EAPC = 0.40; 95% CI 0.33, 0.47). For early-onset dementia, the ASPR (range of EAPC = 0.03–0.17) and ASIR (range of EAPC = 0.05–0.19) in the 45 to 59 years age group increased in North Africa and Middle East, particularly among females (ASPR: range of EAPC = 0.08–0.21; ASIR: range of EAPC = 0.09–0.2). Although the ASMR and ASDR both decreased globally and in high socio-demographic index region, they both increased greatly in South Asia (ASMR: EAPC = 0.7; 95% CI 0.66, 0.75; ASDR: EAPC = 0.40; 95% CI 0.37, 0.43). For early-onset dementia, the ASMR (range of EAPC = 0.43–0.78) and ASDR (range of EAPC = 0.19–0.33) in the 40 to 59 years age group had increased in Central Sub-Saharan Africa, particularly among females (ASMR: range of EAPC = 0.5–0.86; ASDR: range of EAPC = 0.19–0.4). Negative correlations were observed between the EAPC in ASPR, ASIR, ASMR and ASDR with various types of HRH at the national level.

**Conclusions:**

Although there has been progresses in controlling the global trends of dementia incidence, prevalence, and mortality, significant regional and national inequalities remain evident. More importantly, the early-onset dementia burden is increasing significantly among females in the African region, underscoring the critical need for healthcare systems that address dementia across the early midlife span.

**Supplementary Information:**

The online version contains supplementary material available at 10.1186/s41256-025-00417-x.

## Background

Dementia, recognized as the leading neurological burden globally, encompasses various challenges related to memory, learning, concentration, and decision-making [1]. It is a significant cause of disability and dependency among older adults worldwide [1]. The *Global action plan on the public health response to dementia 2017–2025*, published in May 2017, projected that the number of older adults with dementia would increase to 75 million by 2030 and 132 million by 2050 [2]. Dementia has been identified as a public health priority. In the absence of a cure or accessible treatment, risk reduction remains the most feasible and proactive approach to combat dementia. The fifth target of this plan emphasizes that the importance of integrating the reduction and control of modifiable dementia risk factors into national health planning processes and development agendas [2]. In 2023, the World Alzheimer Report urged governments to increase investment in research focused on risk reduction, fostering new insights and innovations to promote healthy aging [3]. Therefore, understanding the disparities in dementia burden is essential for resources allocation and achieving universal health coverage worldwide.

The increasing aging population worldwide, which means a longer life expectancy due to advancements in healthcare over the past century, may lead to a potential rise in the number of individuals affected by dementia. Additionally, national variations in the progress of the dementia action plan may alter the current distribution and disparities of dementia burden. In 2021, the World Health Organization (WHO) released *Global status report on the public health response to dementia* to support its Triple Billion Targets and to achieve the United Nations Sustainable Development Goals (SDGs) [4]. This report highlighted the inadequate implementation of actions and significant disparities in dementia plans (stand-alone, integrated or subnational), awareness-raising campaigns, dementia care services, health information systems and monitoring, as well as dementia research and innovation across member states [4]. For instance, fewer than 27% of WHO member states have established a dementia plan and awareness-raising campaign. The WHO recommends conducting multidomain intervention trials to identify evidence-based approaches to public health preventive interventions; however, such initiatives remain severely lacking in low- and middle-income countries (LMICs) [5]. It is important to note that these inequalities in health resources may exacerbate the disparity in dementia burden between countries. Wang et al. [6] found that health resources were negatively associated with disability-adjusted life years (DALYs) and deaths attributable to dementia. Health resources may influence a nation’s capacity for prevention, treatment, and care for dementia, making it is essential to analyze the relationship between health resources and trends in dementia burden.

The Global Burden of Diseases, Injuries, and Risk Factors Study (GBD) is an international collaboration that employs a consistent methodology and utilizes all available population-level data to generate population health metrics. This initiative offers a unique opportunity to analyze disease trends on a global scale. In this article, we utilized GBD 2021 data to investigate the changes and disparities in dementia burden across 204 countries and territories from 1990 to 2021. The study aimed to analyze and compare temporal trends at regional, national, and sociodemographic levels, providing evidence for public health planning and resource prioritization.

## Methods

### Data source

We utilized data from GBD 2021 which provided the most current estimates of descriptive epidemiological data for a total of 371 diseases and injuries across 204 countries and territories from 1990 to 2021 [7, 8]. Alzheimer's disease and other dementias were mapped according to the GBD cause list using the following International Classification of Diseases and Injuries (ICD) codes: F00–F02.0, F02.8–F03.91, F06.2, G30–G31.1, G31.8–G32.89 (ICD-10), and 290–290.9, 294.0–294.9, 331–331.2, 331.6–331.7, 331.82, 331.89–331.9 (ICD-9) [7, 8]. The Institute for Health Metrics and Evaluation at the University of Washington, USA, coordinated the GBD study as a systematic and scientific effort to quantify the comparative magnitude of health losses due to diseases by sex, age, region, and country over time [9]. The GBD network employed standardized tools within a Bayesian framework to leverage all available data. All disease estimates from GBD include 95% uncertainty intervals (UI) for every metric, which are based on the 2.5th and 97.5th ordered values from 1,000 draws of the posterior distribution [7, 8]. Detailed methods are described elsewhere [7, 8]. For GBD studies, the Institutional Review Board of the University of Washington reviewed and approved a waiver of informed consent.

### Socio-demographic index, healthcare access and quality index, human resources for health, and demographics

The socio-demographic index (SDI) for each country, a a composite indicator, is the geometric mean of 0 to 1 indices of total fertility rate under the age of 25, mean education for those ages 15 and older, and lag distributed income per capita [10]. A higher SDI value indicates a theoretically higher level of development status, which is relevant to health outcomes. All 204 countries or territories were classified into five SDI regions: low, low-middle, middle, high-middle, and high SDI regions. The healthcare access and quality (HAQ) index quantifies healthcare access and quality over time in a comparable way across countries, with higher values indicating superior levels of healthcare access and quality [11]. Human resources for health (HRH) encompass a diverse range of professions dedicated to improving human health [12]. The GBD Human Resources for Health Collaborators applied spatiotemporal Gaussian process regression (ST-GPR) to model HRH densities (e.g., all health workers, physicians, personal care workers, and psychologists per 10, 000 population) across all countries and territories [12]. ST-GPR, a three-stage modeling approach, is frequently utilize in GBD studies due to its ability to accommodate the incomplete or sparse data, variations across geography and time, and quantify uncertainty. In brief, ST-GPR first fits a linear regression to the data with fixed effects, then smooths the residuals between the regression fit and the data across time and geography to generate a non-linear trend. Finally, this trend is used as a mean function in a Gaussian process regression to account for input data variance and to generate UIs [12]. Based on epidemiological homogeneity and geographical contiguity, all 204 countries or territories were categorized into 21 GBD regions, such as High-income Asia Pacific, Central Asia, and others [7, 8]. Additionally, we examined the gender (male, female) and age distribution (40–44, 45–49, 50–54, 55–59, 60–64, 65–69, 70–74, 75–79, 80 + years) of dementia burden. Notably, dementia burden before 65 years old was defined as the burden of early-onset dementia.

### Statistical analysis

The absolute numbers, along with 95% UIs for prevalent cases, incident cases, deaths, and DALYs, as well as their age-standardized rates (ASR) with 95% UIs, were utilized to indicate the epidemic status [13]. The relative changes in absolute numbers from 1990 to 2021 were calculated, defined as $\frac{{{\text{number}}_{2021} - {\text{number}}_{1990} }}{{{\text{number}}_{1990} }} \times 100{\text{\% }}$. Estimated annual percentage change (EAPC) is a summary and widely used measure to assess the trend of ASR over a specified time interval, taking into account the gradual changes in ASR for consecutive years by setting the natural logarithm of ASR. It was calculated by fitting the regression line: y = α + βx + ε, where y = ln (ASR), x = calendar year, β coefficient represents the geometric mean ratio, and ε is the error term, which is assumed to follow a normal distribution with a mean of zero and constant variance. We calculated the EAPC as 100 × (${\text{e}}^{{\upbeta }} - 1$) to assess the temporal trend, along with the corresponding 95% confidence intervals (95% CI). If both the annual percentage change estimates and the 95% CI were greater than 0 (or both less than 0), we considered the corresponding rate to be in an upward (or downward) trend [13, 14].

Locally Estimated Scatterplot Smoothing (Loess) fitting and Pearson correlation analysis were employed to examine the relationship between the SDI, HAQ index, and HRH density in relation to the temporal trends of dementia burden. A hierarchical cluster analysis was performed to categorize countries and territories into four groups based on the temporal trends in dementia burden. All analyses were conducted using R (version 4.4.0). A two-tailed P-value of less than 0.05 was considered statistically significant.

## Results

### Prevalence and incidence of dementia

The global absolute number of prevalent cases (160.81%) and incident cases (156.54%) both increased from 1990 to 2021 (Tables S1, S2). The age-standardized prevalence rate (ASPR, EAPC = 0.005; 95% CI − 0.02, 0.03) and age-standardized incidence rate (ASIR, EAPC = − 0.02; 95% CI − 0.04, − 0.005) were stable and showed a downtrend worldwide, respectively. However, ASPR and ASIR both increased in East Asia (ASPR: EAPC = 0.43; 95% CI 0.35, 0.51; ASIR: EAPC = 0.40; 95% CI 0.33, 0.47) and High-income Asia Pacific (ASPR: EAPC = 0.28; 95% CI 0.23, 0.33; ASIR: EAPC = 0.19; 95% CI 0.14, 0.24). Among 204 countries and territories, the number of countries or territories with an uptrend in ASPR (Figure S1 and Table S3) and ASIR was 23 (Figure S2 and Table S4) and 20, respectively. The highest ASPR was 900.82 per 100,000 population (95% UI: 770.92, 1043.22), and the highest ASIR was 151.47 per 100,000 population (95% UI: 131.22, 173.34) in China in 2021. Both ASPR and ASIR showed the highest increasing trend (ASPR: EAPC = 0.44; 95% CI: 0.36, 0.52; ASIR: EAPC = 0.41; 95% CI 0.34, 0.49) in China.

**Fig. 1:**
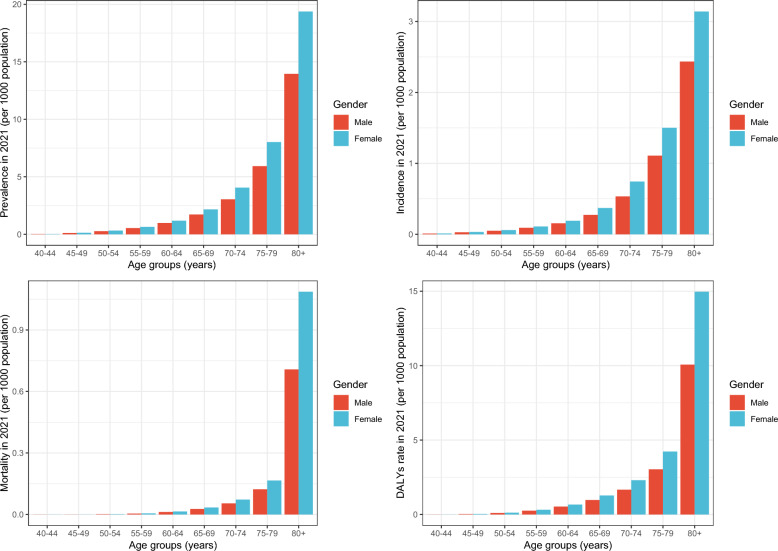
Global dementia burden by gender and age groups in 2021

**Fig. 2:**
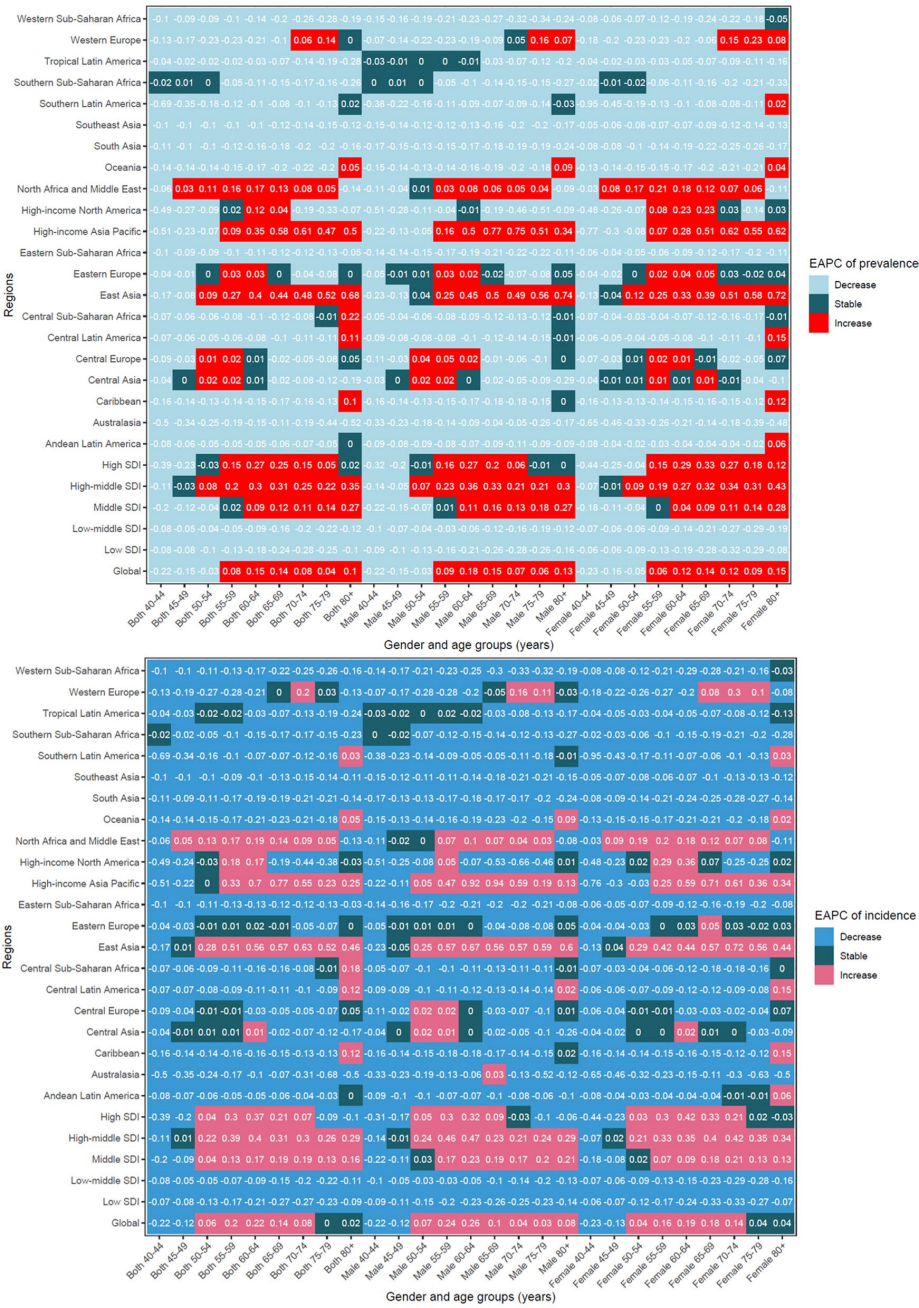
The global and regional EAPC of age-standardized prevalence rate and age-standardized incidence rate by the gender and age group. *Notes*: *EAPC* estimated annual percentage change

The ASPR and ASIR were higher in females than in males worldwide across all age groups in 2021 (Table S1, S2 and Fig. 1). The disparity in ASPR and ASIR between females and males increased with advancing age (Fig. 1). Except 40–50 years age group, the ASPR and ASIR both increased globally in most other age groups (Fig. 2). The ASPR (range of EAPC = 0.03–0.17) and ASIR (range of EAPC = 0.05–0.19) for early-onset dementia in the 45 to 64 years age group has increased in North Africa and Middle East, particularly among females (ASPR: range of EAPC = 0.08–0.21; ASIR: range of EAPC = 0.09–0.2).

**Fig. 3:**
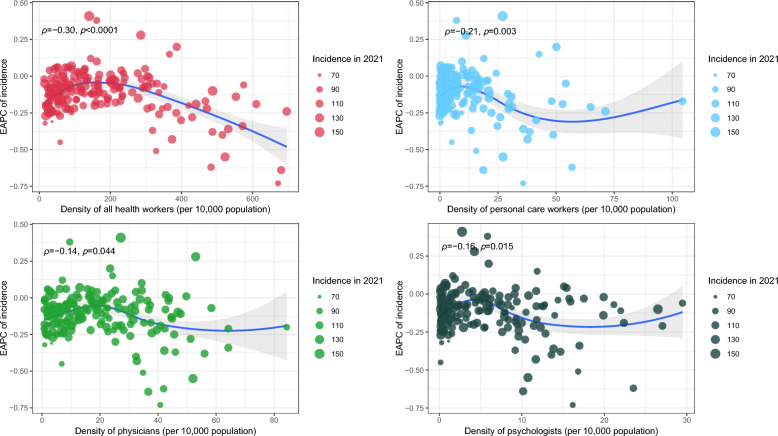
The correlations between EAPC of age-standardized incidence rate and HRH. *Notes*: *EAPC* estimated annual percentage change; *HRH* human resources for health

There were no correlations between the EAPC in ASPR and ASIR with SDI and HAQ index (all *P* > 0.05) (Figure S3). Significant negative correlations were detected between the EAPC in ASPR and the density of all health workers (ρ = − 0.25, *P* < 0.001) and personal care workers (ρ = − 0.16, *P* < 0.001) (Figure S4). Significant negative correlations were detected between the EAPC in ASIR and the density of all health workers (ρ = − 0.30, *P* < 0.0001), followed by personal care workers (ρ = − 0.21, *P* = 0.003), psychologists (ρ = − 0.16, *P* = 0.015), and physicians (ρ = − 0.14, *P* = 0.044) (Fig. 3).

### Mortality rate and DALYs rate of dementia

The global absolute number of deaths (194.39%) and DALYs (167.7%) both increased from 1990 to 2021 (Table 1 and Table S5). Although the age-standardized mortality rate (ASMR) and age-standardized DALYs rate (ASDR) both decreased worldwide, they increased in different SDI regions, except high SDI region. The ASMR and ASDR both increased the most in low SDI region (ASMR: EAPC = 0.45; 95% CI 0.37, 0.53; ASDR: EAPC = 0.24; 95% CI 0.18, 0.29), followed by low-middle SDI region (ASMR: EAPC = 0.43; 95% CI 0.41, 0.45; ASDR: EAPC = 0.23; 95% CI 0.22, 0.24). Among 21 GBD regions, South Asia had the largest increasing trend of ASMR (EAPC = 0.7; 95% CI 0.66, 0.75) and ASDR (EAPC = 0.40; 95% CI 0.37, 0.43), followed by Central Sub-Saharan Africa (ASMR: EAPC = 0.51; 95% CI 0.45, 0.57; ASDR: EAPC = 0.36; 95% CI 0.33, 0.4) and Southern Sub-Saharan Africa (ASMR: EAPC = 0.41; 95% CI 0.4, 0.42; ASDR: EAPC = 0.24; 95% CI 0.23, 0.25). Among 204 countries and territories, the number of countries or territories which showed an uptrend in ASMR (Table S6) and ASDR (Figure S5 and Table S7) was 64 and 49, respectively. The highest increasing trend of ASMR (EAPC = 0.89; 95% CI 0.83, 0.95) and ASDR (EAPC = 0.52; 95% CI 0.48, 0.55) were observed in India. The highest ASMR (35.44 per 100,000 population; 95% UI: 8.35, 93.78) and ASDR (600.24 per 100,000 population; 95% UI: 256.06, 1393.32) were observed in the Democratic Republic of the Congo (DRC) in 2021.

**Table 1:** The deaths and age-standardized mortality rate for dementia and their temporal change at global and regional level, 1990–2021

Regions	Deaths (95% UI) per 1000 population			Age-standardized mortality rate (95% UI), per 100,000 population		
	1990	2021	Percentage change (%)	1990	2021	EAPC (95% CI)
Global	663.29 (163.58, 1764.99)	1952.68 (512.98, 4984.74)	194.39	25.04 (6.29, 66.28)	25.16 (6.68, 64.25)	− 0.02 (− 0.03, − 0.01)
Gender						
Male	200.38 (47.76, 550.52)	626.87 (153.87, 1677.85)	212.84	20.18 (4.98, 55.70)	20.71 (5.19, 55.50)	0.07 (0.05, 0.09)
Female	462.91 (115.21, 1215.53)	1325.80 (356.48, 3316.45)	186.41	27.60 (6.96, 71.87)	27.88 (7.48, 69.79)	− 0.01 (− 0.02, 0.005)
Socio-demographic index						
High SDI	281.91 (71.31, 738.79)	719.33 (196.68, 1762.39)	155.17	27.06 (6.97, 70.01)	26.21 (7.05, 64.83)	− 0.12 (− 0.13, − 0.11)
High-middle SDI	170.71 (41.72, 462.49)	490.09 (128.59, 1265.12)	187.09	25.91 (6.38, 69.59)	26.42 (7.01, 68.31)	0.02 (0, 0.04)
Middle SDI	130.85 (31.33, 349.33)	493.42 (123.09, 1282.12)	277.09	23.69 (5.75, 63.9)	24.56 (6.3, 64.53)	0.03 (0, 0.05)
Low-middle SDI	58.42 (13.86, 159.85)	189.59 (45.9, 510.23)	224.52	17.72 (4.27, 49.17)	20 (4.85, 54.05)	0.43 (0.41, 0.45)
Low SDI	20.73 (4.97, 57.26)	58.64 (13.91, 162.64)	182.91	19.56 (4.72, 54.84)	22.07 (5.3, 61.34)	0.45 (0.37, 0.53)
GBD region						
High-income North America	106.65 (27.64, 276.05)	216.71 (58.26, 541.29)	103.21	29.01 (7.56, 74.58)	28.25 (7.49, 71.28)	− 0.11 (− 0.13, − 0.1)
Caribbean	3.24 (0.78, 9.11)	9.07 (2.25, 24.63)	180.14	16.35 (3.92, 44.84)	15.82 (3.87, 43.17)	− 0.1 (− 0.11, − 0.09)
Andean Latin America	2.3 (0.55, 6.18)	7.68 (1.91, 20.68)	234.05	14.54 (3.51, 39.06)	14.08 (3.49, 37.9)	− 0.13 (− 0.15, − 0.11)
Central Latin America	9.71 (2.38, 26.79)	38.74 (9.58, 102.46)	298.96	17.16 (4.23, 46.44)	16.84 (4.16, 44.59)	− 0.07 (− 0.08, − 0.06)
Tropical Latin America	15.92 (3.94, 41.76)	65.23 (17.12, 164.36)	309.78	28.03 (7.29, 73.09)	27.25 (7.13, 68.87)	− 0.06 (− 0.07, − 0.05)
North Africa and Middle East	26.39 (6.4, 70.58)	73.79 (18.12, 190.47)	179.67	28.02 (6.81, 75.27)	25.61 (6.31, 66.8)	− 0.3 (− 0.35, − 0.26)
South Asia	41.79 (9.6, 119.8)	165.38 (39.36, 454.12)	295.72	14.11 (3.23, 40.84)	17.2 (4.1, 47.28)	0.7 (0.66, 0.75)
Central Sub-Saharan Africa	2.51 (0.6, 6.84)	8.11 (1.93, 22.19)	223.1	30.39 (7.51, 79.92)	34.89 (8.43, 93.23)	0.51 (0.45, 0.57)
Eastern Sub-Saharan Africa	8.13 (1.97, 22.48)	23.35 (5.59, 64.42)	187.12	24.15 (5.95, 66.84)	27.18 (6.72, 73.25)	0.41 (0.4, 0.42)
Southern Sub-Saharan Africa	3.88 (0.95, 10.57)	7.86 (1.89, 21.83)	102.41	21.61 (5.33, 58.95)	22.7 (5.42, 62.82)	0.14 (0.06, 0.22)
Western Sub-Saharan Africa	8.52 (2.07, 23.46)	20.04 (4.71, 56.35)	135.3	18.02 (4.36, 49.87)	19.25 (4.66, 53.64)	0.29 (0.25, 0.33)
Oceania	0.26 (0.06, 0.73)	0.73 (0.17, 2.04)	186.77	22.93 (5.6, 63.25)	21.08 (5.19, 59.87)	− 0.32 (− 0.35, − 0.3)
Central Asia	7.26 (1.79, 19.9)	11.02 (2.73, 30.44)	51.83	20.66 (5.07, 56.11)	20.07 (5.03, 55.69)	− 0.12 (− 0.13, − 0.11)
Central Europe	22.49 (5.46, 63.37)	49.87 (12.52, 133.19)	121.8	20.75 (5.08, 56.95)	20.46 (5.15, 54.29)	− 0.04 (− 0.05, − 0.03)
East Asia	123.42 (29.18, 331.21)	507.66 (129.18, 1368.54)	311.34	30.87 (7.45, 82.33)	30.41 (7.81, 81.29)	− 0.19 (− 0.23, − 0.15)
Eastern Europe	43.18 (10.54, 122.19)	75.31 (18.26, 209.06)	74.42	21.5 (5.22, 59.64)	20.98 (5.07, 57.85)	− 0.09 (− 0.1, − 0.08)
High-income Asia Pacific	42.16 (10.71, 109.37)	200.16 (57.94, 463.17)	374.73	27.79 (7.27, 71.04)	26.59 (7.46, 62.96)	− 0.1 (− 0.12, − 0.07)
Australasia	5 (1.23, 13.39)	15.25 (4.01, 38.57)	204.78	24.52 (6.14, 64.68)	23.18 (6.03, 58.68)	− 0.17 (− 0.19, − 0.15)
Western Europe	153.04 (38.47, 403.81)	339.57 (90.93, 836.29)	121.88	26.77 (6.83, 69.36)	25.78 (6.82, 64.03)	− 0.11 (− 0.12, − 0.09)
Southeast Asia	30.07 (7.13, 80.14)	98.37 (24.21, 259.33)	227.17	20.67 (4.97, 55.97)	22.64 (5.56, 59.23)	0.24 (0.2, 0.29)
Southern Latin America	7.4 (1.84, 20.36)	18.79 (4.81, 48.96)	153.85	20.61 (5.15, 55.38)	20.06 (5.12, 52.25)	− 0.06 (− 0.07, − 0.04)

*CI* confidence interval; *EAPC* estimated annual percentage change; *GBD* Global Burden of Disease; *SDI* socio-demographic index; *UI* uncertainty interval

**Fig. 4:**
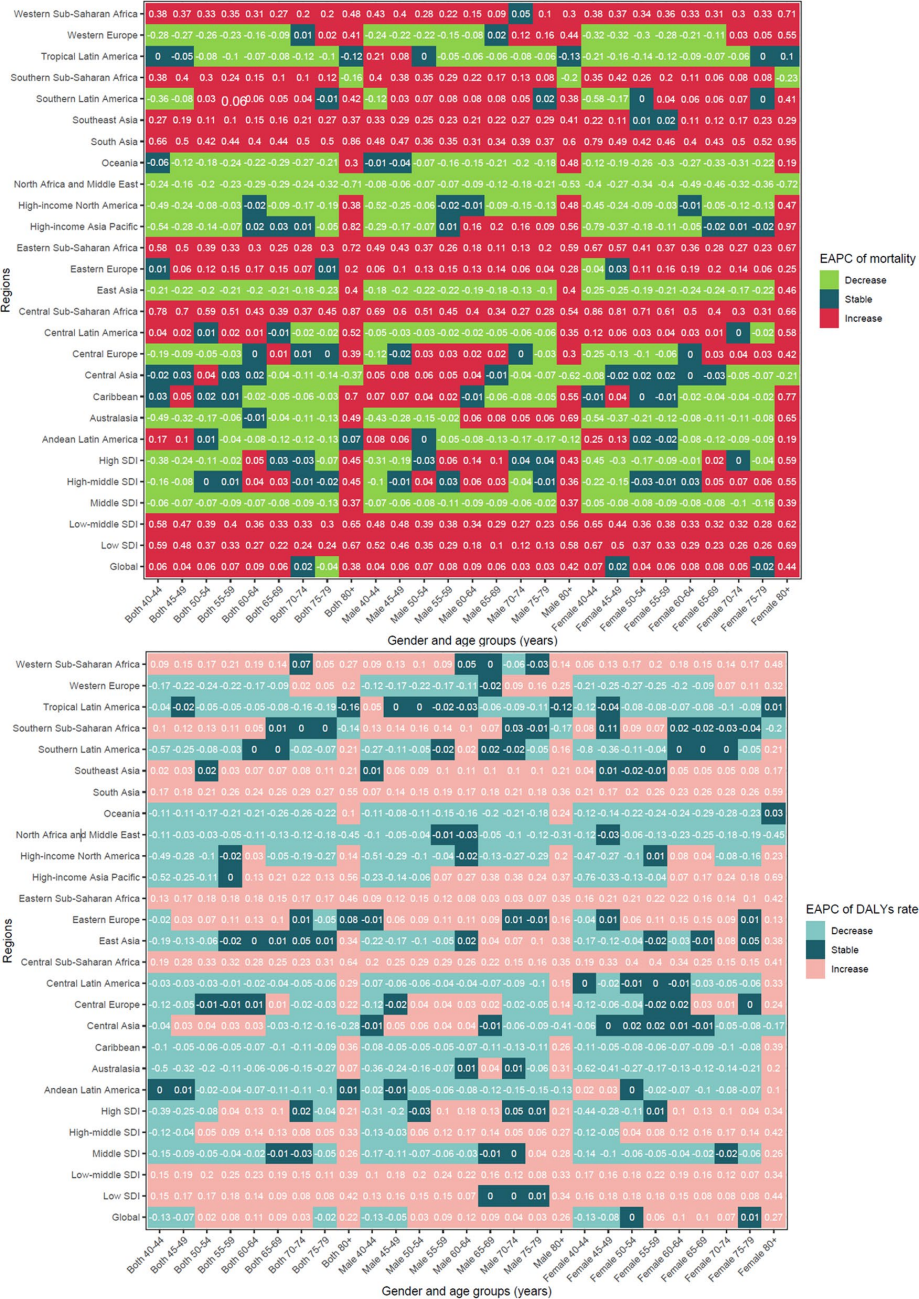
The global and regional EAPC of age-standardized mortality rate and age-standardized DALYs rate by the gender and age group. *Notes*: *DALYs* disability adjusted life years; *EAPC* estimated annual percentage change

The global ASMR and ASDR were higher in females than in males, regardless of age groups (Table 1, Table S5 and Fig. 1). The ASMR increased in all gender and age groups in the South Asia, Eastern Sub-Saharan Africa, and Central Sub-Saharan Africa (Fig. 4). The ASMR (range of EAPC = 0.43–0.78) and ASDR (range of EAPC = 0.19–0.33) of early-onset dementia in 40 to 64 years age group had increased in Central Sub-Saharan Africa, particularly among females (ASMR: range of EAPC = 0.5–0.86; ASDR: range of EAPC = 0.19–0.4).

**Fig. 5:**
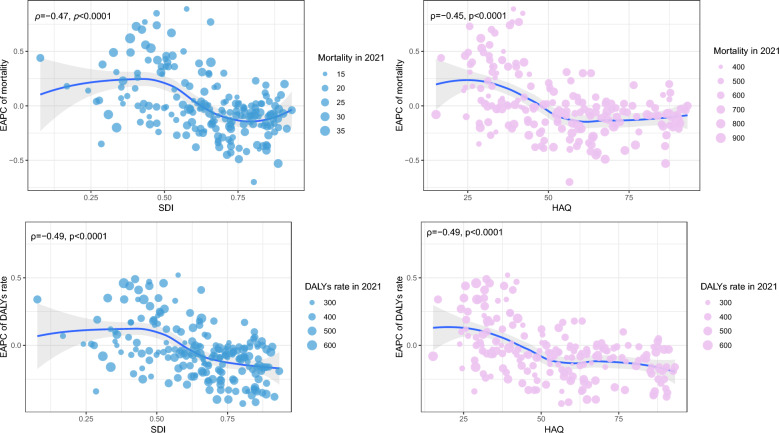
The correlations between EAPC of age-standardized mortality rate, age-standardized DALYs rate, and, SDI and HAQ index. *Notes*: *DALYs* disability adjusted life years; *EAPC* estimated annual percentage change; *HAQ* healthcare access and 1uality index; *SDI* socio-demographic Index

Negative correlations were observed between the EAPC in ASMR and ASDR with SDI and HAQ index (all *P* < 0.001) (Fig. 5). As the level of development status increased (ρ = − 0.47, *P* < 0.001) and health-care access and quality (ρ = − 0.45, *P* < 0.001) improved, a more pronounced decreasing trend in ASMR over time was noted. Significant negative correlations were detected between the EAPC in ASMR and density of all health workers (ρ = − 0.30, *P* < 0.001), physicians (ρ = − 0.30, *P* < 0.001), and psychologists (ρ = − 0.26, *P* < 0.001), except the personal care workers (Figure S6). Significant negative correlations were detected between the EAPC in ASDR and the density of all health workers (ρ = − 0.38, *P* < 0.0001), followed by physicians (ρ = − 0.35, *P* = 0.044), psychologists (ρ = − 0.31, *P* = 0.015), and personal care workers (ρ = − 0.18, *P* = 0.003) (Figure S7). A hierarchical cluster analysis revealed that there were 11 countries and territories (Maldives, Solomon Islands, Georgia, Slovenia, Latvia, Lithuania, Brunei Darussalam, DRC, Rwanda, Zambia, and Nauru) exhibited a concurrent increasing trend in ASPR, ASIR, ASMR, and ASDR from 2019 to 2021 (Figure S8 and Figure S9).

## Discussion

Dementia is one of the leading causes of neurological burden worldwide. Compared to the previous GBD 2019 publication, our study indicates that, despite notable achievements over the past 32 years, health disparities in dementia burden— particularly in terms of dementia mortality—appear to be widening globally [15, 16]. For instance, the global ASMR shifted from an increasing trend between 1990 and 2019 to a decreasing trend from 1990 to 2021 [16]. However, the ASMR only decreased in high SDI region from 1990 to 2021, suggesting a growing geographical gap in the progress of dementia control initiatives [16]. Our analysis of GBD dementia incidence and prevalence suggests that many countries have experienced a decline in dementia incidence and prevalence. However, countries in East Asia and High-income Asia Pacific, particularly China, have shown little or no progress. Dementia is emerging as one of the leading causes of mortality globally, and mortality due to dementia is expected to increase in the future due to corresponding increases in population growth and aging [16]. Our analysis of GBD mortality rate and DALYs rate suggests that only high SDI region have experienced a decline in GBD mortality rate and DALYs rate. However, economically disadvantaged countries in South Asia, Central Sub-Saharan Africa, and Southern Sub-Saharan Africa have shown no progress. More than one third of countries and territories experienced an increasing trend in mortality rate, particularly in India. The trend of dementia incidence was correlated with supply of HRH (personal care workers, psychologists, physicians). The trend of dementia mortality was commensurate with country-level socioeconomic status and supply of HRH (psychologists, physicians). We also found that the early-onset dementia incidence and mortality are increasing significantly in North Africa and Middle East, and Central Sub-Saharan Africa, respectively, especially among females. This highlights the importance of healthcare systems for dementia that cover the early midlife span.

This study used data from the 2021 GBD study to systematically evaluate global trends and regional differences in dementia burden. Although the prevalence and incidence remained stable or slightly decreased worldwide, the absolute number of prevalent cases and incident cases increased by more than 150% from 1990 to 2021. The substantial number of prevalent cases and incident cases continues to pose a significant challenge [17]. In comparison to other regions, East Asia and High-income Asia Pacific exhibited a notable upward trend in both prevalence and incidence. The concurrent marked regional disparities in the escalation of these rates suggest a potential imbalance in preventive initiatives against dementia over the past several decades. The Global dementia action plan address this issue by establishing a global target for 75% of WHO Member States to have developed or updated a national policy, strategy, plan, or framework for dementia—either as a stand-alone initiative or integrated into other policies—by 2025 [4, 17]. However, only 50 countries (26% of Member States) met this target in 2021 [4, 17]. Meanwhile, the distribution of national dementia plans is largely uneven across WHO regions, with half of the policies (n = 26) originating from the European Region [4]. Despite a significant number of countries having plans or guidelines for reducing dementia risk, approximately 78% of these countries are high-income countries [4]. The number of countries and territories experiencing increasing prevalence and incidence of dementia has been nearly a quarter of all countries worldwide from 1990 to 2021, which represents a decline compared to the period from 1990 to 2019, underscoring the importance of national dementia plans [18]. Currently, it is imperative that prevention programs be prioritized in the East Asia and the High-income Asia Pacific regions, alongside the ongoing implementation of existing initiatives in other areas. Additionally, we found that the global prevalence, incidence and mortality were higher among females than males. It is important to note that the factors contributing to the observed demographic variations in dementia burden between females and males are complex, involving various biological, social, and environmental influences [19]. Among these, the longer life expectancy of females compared to males is a common potential factor, indicating that females are more likely to reach the age ranges where the risk of dementia is elevated [20]. Furthermore, declining estrogen levels, related oophorectomy, and historically lower education levels among females may also be contribute to this disparity [19]. In any case, the current gender differences in dementia burden suggest that further exploration of the mechanisms underlying these differences is necessary, along with the implementation of measures to mitigate the higher burden experienced by females.

The escalating prevalence and incidence of dementia are placing a significant burden on the public and healthcare systems in China. One meta-analysis showed that the prevalence and incidence of dementia increased with age and over time in China from 2010 to 2020 [21]. Contributing factors may include the growing aging population, changes in lifestyle habits, and a high burden of vascular-metabolic multimorbidity may all be causes [3, 22]. Additionally, a decline in cultural and social engagement may further exacerbate the progression of dementia [23]. In China, despite improved access to health services, inadequate management of dementia remains common, particularly in rural areas [24]. As the primary health care institutions may be more concerned about diabetes, hypertension, and chronic obstructive pulmonary disease, etc. in the past years, the existing three—tier long-term care system is still insufficient to meet the needs of individuals with dementia [25]. China still needs to prioritize future improvement in primary health care institutions, focusing more on prevention of dementia, within the context of the highest ASPR and ASIR in 2021 and the highest increasing trend of these two indicators from 1990 to 2021. Establishing a national surveillance system to monitor the incidence, prevalence, and the risk and protective factors associated with dementia is essential. Our findings indicate that the trend of dementia incidence is correlated with the availability of HRH (personal care workers, psychologists, physicians) from 2019 to 2021. Within the framework of the Chinese healthcare system, community multidisciplinary teams and care home intervention teams in primary and secondary hospitals could be instrumental in achieving the goal of dementia prevention [25–27]. Additionally, multi-center national clinical trial centers should be established, particularly for traditional Chinese medicines, which, despite its popularity among many dementia patients, lacks substantial evidence supporting efficacy [24].

Progress in deceasing dementia-related deaths appears minimal. The global mortality rate has decreased by only 0.02%, yet more than one-third of countries have experienced an upward trend in mortality rates. Specifically, the mortality rate has significantly increased in low SDI region and low-middle SDI region. The trend of dementia mortality are commensurate with country-level socioeconomic status, indicating the need for enhanced support, particularly in LMICs. The *Global status report on the public health response to dementia* shows that community-based services for dementia are more prevalent in high-income countries compared to LMICs [4]. Access to medication for dementia, hygiene products, assistive technologies, and household modifications is more readily available in high-income countries due to higher levels of reimbursement compared to lower-income countries [4]. Recently, there has been a consolidated trend toward multi-component interventions for dementia progression, particularly evident in the World-Wide Finnish Geriatric Intervention Study (WW-FINGERS) to prevent cognitive impairment and disability [5]. The FINGER study highlighted the importance of addressing multiple dementia risk factors as a strategy to protect brain health, promote overall health and reduce the risk of developing new chronic diseases [5, 28, 29]. However, multidomain intervention trials in LMICs are currently lacking [5, 29].

We also found that the early-onset dementia incidence and mortality are increasing most significantly in North Africa and Middle East, and in Central Sub-Saharan Africa, respectively. This suggests that the gaps and barriers hindering effective action may be widening throughout the midlife span. In comparison to the limited number of low-income or lower-middle income countries with a national dementia plan, the number of high-income countries with such plans is considerably higher. Nearly one third of upper-middle-income countries and half of high-income countries have reported national or subnational dementia research plans [4]. Given the complexity of dementia, it cannot be effectively addressed in isolation. The underrepresentation of low-income or lower-middle income countries, due to limited funding and inadequate research capacity, along with the exclusion of individuals with dementia and their caregivers from research development impedes inclusivity [4]. We found that the ASMR increased the most in low SDI region, followed by low-middle SDI region. Additionally, the highest ASMR was observed in the DRC in 2021. It is possible that the lag in relevant research in lower-income countries has made it challenging to address the dementia burden, particularly in terms of reducing mortality. Effectively decreasing the risk of deaths attributed to dementia relies on robust clinical medical evidence and sound policy management. As proposed by the WHO, it is urgent to enhance research collaboration and establish a global coordination mechanism to support worldwide innovation in the approach to dementia [4].

Our study provides an in-depth analysis of dementia trends using the latest GBD data to understand disparities in the prevention and management of dementia within the context of an aging population. A notable limitation of this analysis is that the accuracy and robustness of GBD estimates largely depend on the quality and quantity of data used in the modeling [30]. In countries where national systematic surveillance and population-based studies are lacking or insufficient, the estimates may be subject to bias.

## Conclusions

Although the global trends in incidence, prevalence, and mortality for dementia was downward or stable, notable regional and national disparities in diseases burden have emerged. The observed decline in dementia incidence and prevalence in most countries or territories suggests that previous dementia action plans may have been effective. However, there has been little or no progress in certain countries in East Asia and High-income Asia Pacific, such as China. Controlling dementia mortality remains a challenge, particularly in low SDI region and low-middle SDI region. More than one-third of countries and territories have experienced an increasing trend of mortality, particularly in South Asia, Central Sub-Saharan Africa, and Southern Sub-Saharan Africa. The early-onset dementia incidence and mortality are significantly increasing among females in North Africa and Middle East and Central Sub-Saharan Africa, respectively, highlighting the need for healthcare systems to address dementia throughout the early to midlife span for females. The availability of HRH is correlated with a decreasing trend in both incidence and mortality. Strengthening the construction of multidisciplinary medical teams and enhancing international cooperation in the prevention, treatment, and management of dementia are essential to address the health inequalities in the dementia burden.

## Supplementary Information

Additional file 1.

## Data Availability

The datasets generated during and/or analyzed during the current study are available in the GBD repository, [http://ghdx.healthdata.org/gbd-results-tool.].

## Abbreviations

ASR: Age-standardized rate
CI: Confidence interval
DALYs: Disability adjusted life years
DRC: Democratic Republic of the Cong
EAPC: Estimated annual percentage change
GBD: Global Burden of Diseases, Injuries, and Risk Factors Study
HAQ: Healthcare access and quality
HRH: Human resources for health
ICD: International Classification of Diseases and Injuries
LMICs: Low- and middle-income countries
SDGs: Sustainable Development Goals
SDI: Socio-demographic index
UIs: Uncertainty intervals
WHO: World Health Organization
WW-FINGERS: World-Wide Finnish Geriatric Intervention Study
