# Supplementary material for: Health inequalities in disease burden of dementia and early-onset dementia: findings from the Global Burden of Disease 2021 study
Source: Glob Health Res Policy. 2025 May 19;10:21. doi: 10.1186/s41256-025-00417-x (PMC12087054; doi:10.1186/s41256-025-00417-x)
Supplement: Supplementary file 1 — Additional file 1. [file 41256_2025_417_MOESM1_ESM.docx]

supplement materials

Table S1. The prevalent cases and age-standardized prevalence rate for dementia and their temporal change at global and regional level, 1990−2021 2

Table S2. The incident cases and age-standardized incidence rate for dementia and their temporal change at global and regional level, 1990−2021 4

Figure S1. The age-standardized prevalence rate for dementia and its temporal trend at national level, 1990−2021 6

Table S3. The prevalent cases and age-standardized prevalence rate for dementia and their temporal change at national level, 1990−2021 7

Figure S2. The age-standardized incidence rate for dementia and its temporal trend at national level, 1990−2021 16

Table S4. The incident cases and age-standardized incidence rate for dementia and their temporal change at national level, 1990−2021 17

Figure S3. The correlations between EAPC of age-standardized prevalence rate, age-standardized incidence rate, SDI, and HAQ index. 25

Figure S4. The correlations between EAPC of age-standardized prevalence rate and HRH. 26

Table S5. The DALYs and age-standardized DALYs rate for dementia and their temporal change at global and regional level, 1990−2021 27

Table S6. The deaths and age-standardized mortality rate for dementia and their temporal change at national level, 1990−2021 29

Figure S5. The age-standardized DALYs rate for dementia and its temporal trend at national level, 1990−2021 37

Table S7. The DALYs and age-standardized DALYs rate for dementia and their temporal change at national level, 1990−2021 38

Figure S6. The correlations between EAPC of age-standardized mortality rate and HRH. 47

Figure S7. The correlations between EAPC of age-standardized DALYs rate and HRH. 48

Figure S8. A hierarchy cluster analysis in terms of temporal trends in diseases burden of dementia. 49

Figure S9. The cluster dendrogram of hierarchy cluster analysis in terms of temporal trends in diseases burden of dementia. 50

**Table S1**. **The prevalent cases and age-standardized prevalence rate for dementia and their temporal change at global and regional level, 1990−2021**

|  | **Prevalent cases (95% UI) per 1000 population** | | | **Age-standardized prevalence rate (95% UI), per 100,000 population** | | |
| --- | --- | --- | --- | --- | --- | --- |
| **Regions** | **1990** | **2021** | **Percentage change (%)** | **1990** | **2021** | **EAPC (95% CI)** |
| **Global** | 21799.76 (19067.09, 24837.69) | 56856.69 (49382.06, 64977.51) | 160.81 | 672.22 (588.73, 763.95) | 694.01 (602.88, 794.08) | 0.005 (-0.02, 0.03) |
| **Gender** |  |  |  |  |  |  |
| Male | 7656.32 (6611.28, 8728.10) | 20753.31 (17769.42, 23796.80) | 171.06 | 571.47 (497.63, 654.31) | 589.47 (507.48, 678.79) | 0.03 (0.01, 0.05) |
| Female | 14143.45 (12361.84, 16105.29) | 36103.38 (31468.18, 41117.47) | 155.27 | 736.15 (646.05, 834.27) | 769.94 (670.71, 877.57) | 0.04 (0.01, 0.06) |
| **Socio-demographic index** |  |  |  |  |  |  |
| High-middle SDI | 5680.57 (4933.84, 6518.29) | 14925.1 (12861.05, 17155.67) | 162.74 | 685.28 (598.24, 781.65) | 766.2 (659.8, 879.64) | 0.21 (0.17, 0.25) |
| High SDI | 8093.99 (7092.81, 9188.86) | 17216.18 (15033, 19545.52) | 112.7 | 724.21 (637.6, 815.94) | 709.47 (619.94, 807.4) | -0.05 (-0.06, -0.04) |
| Low-middle SDI | 2350.23 (2033.03, 2676.29) | 6009.38 (5214.01, 6839.57) | 155.69 | 542.48 (473.62, 616.16) | 524.47 (455.82, 596.8) | -0.17 (-0.19, -0.15) |
| Low SDI | 818.15 (700.4, 930) | 1854.99 (1603.96, 2101.15) | 126.73 | 539.74 (471.27, 612.06) | 514.37 (447.22, 584.18) | -0.19 (-0.21, -0.18) |
| Middle SDI | 4831.62 (4192.76, 5504.06) | 16802.25 (14485.31, 19311.41) | 247.76 | 651.47 (566.76, 743.9) | 723.42 (623.26, 830.91) | 0.12 (0.08, 0.17) |
| **GBD region** |  |  |  |  |  |  |
| High-income North America | 3021.8 (2617.28, 3444.67) | 5508.42 (4800.05, 6295.24) | 82.29 | 815.9 (709.63, 929.16) | 775.11 (673.67, 885.78) | -0.21 (-0.23, -0.19) |
| Caribbean | 130.47 (113.21, 148.47) | 301.09 (261.14, 341.2) | 130.77 | 557.24 (485.64, 629.27) | 550.22 (476.89, 624.84) | -0.15 (-0.18, -0.13) |
| Andean Latin America | 78.84 (68.47, 89.84) | 248.16 (215.29, 283.42) | 214.77 | 450.85 (390.47, 514.2) | 444.09 (384.1, 507.73) | -0.06 (-0.07, -0.05) |
| Central Latin America | 420.13 (364.26, 478.95) | 1397.1 (1216.19, 1585.19) | 232.54 | 625.86 (543.81, 713.04) | 596.48 (518.98, 680.63) | -0.11 (-0.12, -0.1) |
| Tropical Latin America | 540.71 (467.91, 618.34) | 1862.09 (1617.47, 2116.52) | 244.38 | 758.68 (660.76, 863.19) | 759.83 (660, 867.54) | -0.05 (-0.09, -0.01) |
| North Africa and Middle East | 977.03 (843.77, 1104.04) | 2684.37 (2322.37, 3040.39) | 174.75 | 812.51 (708.72, 918.84) | 772.66 (671.2, 877.55) | -0.17 (-0.17, -0.16) |
| South Asia | 1759.04 (1517.06, 2008.72) | 5147.48 (4444.26, 5886.07) | 192.63 | 446.47 (386.42, 510.25) | 437.07 (377, 500.95) | -0.18 (-0.22, -0.14) |
| Central Sub-Saharan Africa | 100.18 (85.94, 114.16) | 253.63 (220.06, 287.41) | 153.18 | 752.86 (650.12, 852.75) | 750.54 (654.7, 848.11) | 0.0001 (-0.02, 0.02) |
| Eastern Sub-Saharan Africa | 306.61 (262.59, 348.49) | 692.23 (597.29, 780.54) | 125.77 | 618.54 (538.17, 701.19) | 588.72 (513.88, 667.2) | -0.14 (-0.15, -0.14) |
| Southern Sub-Saharan Africa | 137.24 (118.25, 156.39) | 268.8 (231.16, 306.09) | 95.86 | 639.88 (554.13, 730.43) | 606.69 (524.69, 692.07) | -0.14 (-0.15, -0.13) |
| Western Sub-Saharan Africa | 281.67 (241.9, 319.13) | 573.97 (493.78, 650.72) | 103.77 | 436.85 (379.4, 496.47) | 406.02 (352.89, 462.39) | -0.24 (-0.26, -0.22) |
| Oceania | 11.66 (9.92, 13.44) | 30.7 (26.25, 34.93) | 163.21 | 676.8 (584.15, 778.03) | 644.83 (554.28, 737.37) | -0.2 (-0.22, -0.18) |
| Central Asia | 256.02 (221.62, 290.36) | 398.79 (345.21, 451.92) | 55.77 | 638.07 (553.08, 725.58) | 626.81 (541.85, 713.52) | -0.06 (-0.07, -0.04) |
| Central Europe | 868.66 (747.73, 997.94) | 1540.94 (1327.85, 1766.51) | 77.39 | 657.16 (565.4, 750.94) | 641.22 (554.05, 732.31) | -0.09 (-0.09, -0.08) |
| East Asia | 4151.55 (3556.09, 4768.97) | 17414.17 (14854.02, 20142.13) | 319.46 | 697.26 (603.5, 802.56) | 887.95 (759.95, 1027.48) | 0.43 (0.35, 0.51) |
| Eastern Europe | 1653.42 (1425, 1903.78) | 2380.52 (2059.57, 2730.95) | 43.98 | 669.2 (578.77, 768.96) | 658.68 (571.31, 752.67) | -0.08 (-0.12, -0.04) |
| High-income Asia Pacific | 1182.07 (1025.97, 1345.12) | 4109.52 (3547.34, 4692.56) | 247.65 | 658.06 (572.1, 745.38) | 684.82 (596.97, 780.11) | 0.28 (0.23, 0.33) |
| Australasia | 160.27 (139.13, 181.94) | 363.51 (317.55, 409.19) | 126.81 | 706.96 (614.59, 802.27) | 604.41 (526.77, 679.94) | -0.54 (-0.58, -0.51) |
| Western Europe | 4233.26 (3694.05, 4798.87) | 7747.19 (6705.23, 8805.14) | 83.01 | 691.03 (608.15, 774.18) | 670.36 (583.83, 762.62) | -0.11 (-0.14, -0.08) |
| Southeast Asia | 1269.36 (1094.13, 1445.54) | 3387.8 (2922.77, 3857.74) | 166.89 | 675.02 (589.62, 770.17) | 644.38 (560.58, 737.69) | -0.14 (-0.16, -0.12) |
| Southern Latin America | 259.76 (223.39, 295.89) | 546.19 (471.61, 621.96) | 110.27 | 622.39 (538.28, 706.11) | 595.26 (514.48, 676.41) | -0.15 (-0.16, -0.14) |

Note: CI, confidence interval; EAPC, estimated annual percentage change; GBD, Global Burden of Disease; SDI, socio-demographic index; UI, uncertainty interval

**Table S2**. **The incident cases and age-standardized incidence rate for dementia and their temporal change at global and regional level, 1990−2021**

|  | **Incident cases (95% UI) per 1000 population** | | | **Age-standardized incidence rate (95% UI), per 100,000 population** | | |
| --- | --- | --- | --- | --- | --- | --- |
| **Regions** | **1990** | **2021** | **Percentage change (%)** | **1990** | **2021** | **EAPC (95% CI)** |
| **Global** | 3834.53 (3367.54, 4358.43) | 9837.06 (8620.52, 11163.7) | 156.54 | 116.97 (102.77, 132.32) | 119.76 (104.96, 135.89) | -0.02 (-0.04, -0.005) |
| **Gender** |  |  |  |  |  |  |
| Male | 1352.32 (1177.68, 1551.79) | 3645.49 (3144.74, 4183.54) | 169.57 | 100.69 (88.05, 114.43) | 103.40 (89.45, 118.45) | 0.02 (-0.001, 0.03) |
| Female | 2482.21 (2183.97, 2820.92) | 6191.56 (5432.75, 7009.23) | 149.44 | 127.82 (112.82, 144.18) | 132.29 (116.30, 149.80) | 0.001 (-0.02, 0.03) |
| **Socio-demographic index** |  |  |  |  |  |  |
| High-middle SDI | 990.61 (862.2, 1138.43) | 2582.35 (2252.03, 2941.77) | 160.68 | 118.38 (103.82, 134.11) | 132.4 (115.43, 150.85) | 0.22 (0.18, 0.26) |
| High SDI | 1435.43 (1264.75, 1624.79) | 2952.15 (2586.72, 3344.55) | 105.66 | 127.18 (112.55, 142.92) | 122.61 (107.45, 138.44) | -0.11 (-0.12, -0.1) |
| Low-middle SDI | 415.38 (363.22, 471.14) | 1063.28 (929.61, 1207.79) | 155.98 | 95.55 (83.27, 108.9) | 92.61 (80.79, 105.71) | -0.17 (-0.18, -0.15) |
| Low SDI | 144.16 (125.72, 163.57) | 328.7 (287.11, 372.98) | 128 | 95.08 (82.74, 108.32) | 90.89 (79, 103.12) | -0.19 (-0.2, -0.17) |
| Middle SDI | 844.51 (735.19, 963.6) | 2902.08 (2542.32, 3314.77) | 243.64 | 113.27 (99.18, 128.9) | 123.79 (108.25, 141.26) | 0.09 (0.05, 0.14) |
| **GBD region** |  |  |  |  |  |  |
| High-income North America | 520.2 (454.54, 593.02) | 928.26 (812.57, 1051.34) | 78.44 | 139.57 (122.14, 157.89) | 131.39 (114.67, 149.04) | -0.22 (-0.24, -0.21) |
| Caribbean | 22.91 (19.92, 26.13) | 52.4 (45.97, 59.45) | 128.75 | 97.6 (85.15, 110.9) | 95.6 (83.53, 108.64) | -0.16 (-0.19, -0.14) |
| Andean Latin America | 14.09 (12.25, 15.98) | 44.49 (38.61, 50.84) | 215.64 | 80.6 (69.91, 92.12) | 79.55 (68.95, 91.05) | -0.06 (-0.07, -0.05) |
| Central Latin America | 74.98 (65.39, 85.45) | 248.66 (218.26, 282.09) | 231.64 | 111.25 (96.71, 126.56) | 106.12 (92.53, 120.99) | -0.11 (-0.12, -0.09) |
| Tropical Latin America | 92.95 (81.38, 105.41) | 311.56 (274.8, 352.66) | 235.2 | 129.14 (113.1, 146.66) | 126.83 (111.77, 144.42) | -0.11 (-0.15, -0.08) |
| North Africa and Middle East | 170.21 (149.71, 192.33) | 468.05 (411.55, 530.96) | 174.98 | 138.06 (121.37, 156.98) | 132.19 (115.75, 150.35) | -0.14 (-0.15, -0.13) |
| South Asia | 315.18 (273.89, 359.43) | 924.83 (799.98, 1056.26) | 193.43 | 80.57 (69.5, 92.11) | 79 (68.26, 90.52) | -0.18 (-0.22, -0.14) |
| Central Sub-Saharan Africa | 17.48 (15.15, 19.94) | 44.06 (38.63, 49.58) | 152.02 | 126.9 (111.06, 144.4) | 126.14 (111.31, 143.21) | -0.01 (-0.04, 0.01) |
| Eastern Sub-Saharan Africa | 53.37 (46.29, 60.69) | 121.21 (106.42, 137.29) | 127.13 | 107.12 (93.44, 121.63) | 102.41 (89.7, 116.09) | -0.13 (-0.14, -0.12) |
| Southern Sub-Saharan Africa | 24.23 (21.09, 27.41) | 47.44 (41.32, 54.03) | 95.83 | 112.76 (98.34, 128.64) | 107.13 (93.16, 122.19) | -0.14 (-0.15, -0.12) |
| Western Sub-Saharan Africa | 49.77 (43.26, 56.44) | 102 (88.93, 115.14) | 104.95 | 78.42 (67.93, 89.46) | 73.18 (63.36, 83.46) | -0.23 (-0.24, -0.21) |
| Oceania | 2.06 (1.76, 2.37) | 5.42 (4.71, 6.17) | 163.47 | 117.17 (102.35, 133.78) | 112.05 (96.98, 128.81) | -0.18 (-0.2, -0.16) |
| Central Asia | 44.78 (39.05, 51.31) | 69.75 (61.21, 79.09) | 55.76 | 111.72 (97.73, 127.69) | 109.82 (95.87, 125.3) | -0.06 (-0.07, -0.04) |
| Central Europe | 152.64 (131.92, 177.17) | 269.69 (233.1, 309.82) | 76.69 | 115.01 (100.69, 131.62) | 112.42 (97.97, 128.46) | -0.08 (-0.09, -0.07) |
| East Asia | 725.92 (621.29, 834.63) | 2988.72 (2569.17, 3434.39) | 311.71 | 120.29 (104.75, 137.02) | 149.61 (129.58, 171.14) | 0.4 (0.33, 0.47) |
| Eastern Europe | 291.03 (251.71, 335.61) | 416.09 (361.58, 476.87) | 42.97 | 117.31 (102.49, 133.78) | 115.66 (100.94, 131.87) | -0.08 (-0.11, -0.04) |
| High-income Asia Pacific | 212.52 (185.44, 242.78) | 701.76 (614.64, 802.67) | 230.21 | 116.94 (102.53, 133.08) | 118.62 (103.43, 135) | 0.19 (0.14, 0.24) |
| Australasia | 28.41 (24.87, 32.32) | 63.29 (55.46, 71.35) | 122.78 | 123.79 (108.48, 139.61) | 105.44 (92.56, 118.63) | -0.56 (-0.6, -0.52) |
| Western Europe | 758.29 (671.2, 848.02) | 1352.81 (1182.31, 1543.87) | 78.4 | 122.45 (108.7, 136.67) | 118.56 (103.36, 134.23) | -0.14 (-0.18, -0.11) |
| Southeast Asia | 216.53 (189.08, 245.89) | 578.24 (505.93, 658.51) | 167.05 | 114.85 (100.81, 130.85) | 110.07 (96.11, 125.72) | -0.14 (-0.15, -0.12) |
| Southern Latin America | 46.98 (40.64, 53.79) | 98.31 (85.03, 112.62) | 109.29 | 111.77 (97.43, 127.36) | 107.09 (92.9, 122.43) | -0.14 (-0.15, -0.13) |

Note: CI, confidence interval; EAPC, estimated annual percentage change; GBD, Global Burden of Disease; SDI, socio-demographic index; UI, uncertainty interval


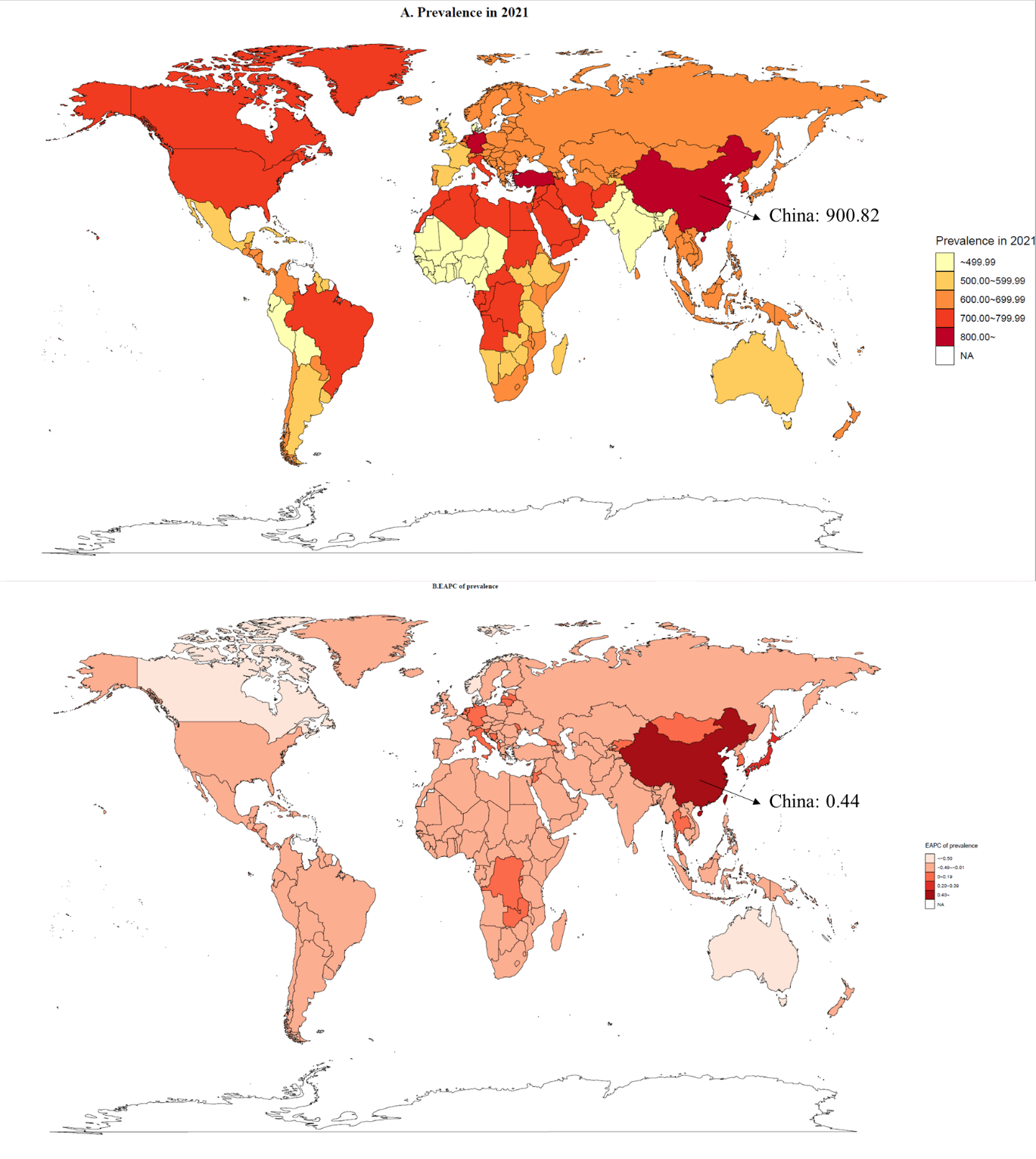


**Figure S1**. **The age-standardized prevalence rate for dementia and its temporal trend at national level, 1990−2021**

Note: EAPC, estimated annual percentage change

**Table S3**. **The prevalent cases and age-standardized prevalence rate for dementia and their temporal change at national level, 1990−2021**

|  | **Prevalent cases (95% UI) per 1000 population** | | | **Age-standardized prevalence rate (95% UI), per 100, 000 population** | | |
| --- | --- | --- | --- | --- | --- | --- |
| **Countries and territories** | **1990** | **2021** | **Percentage change (%)** | **1990** | **2021** | **EAPC (95% CI)** |
| Afghanistan | 38.68 (33.03, 44.21) | 49.01 (42.19, 56.06) | 26.72 | 777.91 (674.21, 886.04) | 761.83 (655.07, 867.52) | -0.07 (-0.09, -0.06) |
| Albania | 10.62 (9.17, 12.09) | 27.83 (23.95, 32.08) | 162.09 | 646.38 (556.6, 736.69) | 648.77 (562.35, 743.87) | 0.03 (0.02, 0.04) |
| Algeria | 68.76 (58.28, 79.85) | 213.9 (183.63, 246.72) | 211.1 | 787.05 (675.35, 896.79) | 752.64 (650.93, 866.12) | -0.16 (-0.17, -0.15) |
| American Samoa | 0.09 (0.08, 0.11) | 0.23 (0.2, 0.27) | 151.99 | 640.58 (540.81, 740.34) | 626.63 (528.15, 722.51) | -0.09 (-0.1, -0.08) |
| Andorra | 0.34 (0.29, 0.39) | 1.09 (0.93, 1.24) | 220.37 | 688.92 (594.24, 791.59) | 645.09 (551.71, 741.3) | -0.21 (-0.21, -0.2) |
| Angola | 16.85 (14.49, 19.17) | 52.58 (45.27, 59.75) | 212 | 760.96 (656.94, 865.61) | 738.83 (636.99, 840.23) | -0.1 (-0.1, -0.09) |
| Antigua and Barbuda | 0.33 (0.28, 0.38) | 0.5 (0.43, 0.58) | 52.73 | 559.11 (482.15, 639.66) | 545.71 (468.4, 624.81) | -0.07 (-0.08, -0.06) |
| Argentina | 182.12 (156.92, 207.95) | 347.97 (301.07, 398.59) | 91.07 | 624.58 (540.01, 715.42) | 592.53 (512.44, 676.59) | -0.18 (-0.2, -0.16) |
| Armenia | 14.7 (12.69, 16.81) | 28.42 (24.4, 32.44) | 93.37 | 655.25 (565.59, 746.77) | 651.84 (563.19, 741.68) | -0.02 (-0.03, -0.01) |
| Australia | 132.33 (114.61, 150.48) | 300.59 (262.04, 338.69) | 127.15 | 702.82 (607.11, 797.96) | 589.93 (513.23, 663.34) | -0.6 (-0.64, -0.56) |
| Austria | 89.76 (76.56, 103.58) | 143.9 (122.26, 167.78) | 60.31 | 700.14 (604.44, 803.39) | 654.27 (558.12, 755.81) | -0.26 (-0.29, -0.23) |
| Azerbaijan | 26.7 (23.15, 30.49) | 47.76 (41.32, 54.59) | 78.85 | 650.14 (562.27, 745.41) | 629.51 (541.15, 721.59) | -0.13 (-0.15, -0.11) |
| Bahamas | 0.72 (0.62, 0.82) | 1.86 (1.61, 2.12) | 156.85 | 557.67 (479.49, 634.59) | 545.68 (469.68, 625.79) | -0.08 (-0.09, -0.07) |
| Bahrain | 0.75 (0.64, 0.86) | 3.85 (3.3, 4.4) | 416.45 | 789.09 (678.43, 906.42) | 765.69 (660.19, 875.62) | -0.07 (-0.09, -0.06) |
| Bangladesh | 170.71 (147.05, 194.56) | 516.77 (446.67, 592.1) | 202.72 | 461.65 (396.87, 526.02) | 444.05 (383.22, 506.23) | -0.13 (-0.14, -0.12) |
| Barbados | 1.84 (1.58, 2.13) | 2.86 (2.45, 3.27) | 55.05 | 565.67 (492.06, 646.55) | 542.29 (465, 620.45) | -0.14 (-0.16, -0.12) |
| Belarus | 82.52 (70.63, 95.2) | 110.57 (95.06, 127.56) | 33.99 | 670.45 (576.02, 769.54) | 668.51 (574.81, 769.2) | 0 (-0.01, 0.02) |
| Belgium | 128.42 (111.01, 147.4) | 198.77 (169.53, 229.9) | 54.78 | 787.5 (685.21, 897.7) | 693.78 (598.36, 799.83) | -0.46 (-0.48, -0.43) |
| Belize | 0.52 (0.45, 0.6) | 1.39 (1.2, 1.58) | 165.73 | 580.72 (501.76, 665.24) | 557.96 (479.96, 636.81) | -0.12 (-0.12, -0.11) |
| Benin | 7.82 (6.72, 8.91) | 16.56 (14.5, 18.97) | 111.78 | 479.13 (412.99, 546.66) | 428.5 (376.12, 490.81) | -0.25 (-0.29, -0.21) |
| Bermuda | 0.32 (0.28, 0.37) | 0.87 (0.75, 1) | 167.95 | 576.86 (495.36, 659.37) | 567.27 (489.1, 648.4) | -0.06 (-0.06, -0.05) |
| Bhutan | 0.73 (0.62, 0.83) | 2.32 (2, 2.67) | 218.81 | 472.18 (407.19, 539.39) | 433.7 (372.38, 497.86) | -0.27 (-0.29, -0.24) |
| Bolivia (Plurinational State of) | 11.2 (9.6, 12.82) | 33.65 (28.94, 38.35) | 200.49 | 464.08 (398.47, 530.3) | 456.33 (392.47, 522.57) | -0.02 (-0.04, 0) |
| Bosnia and Herzegovina | 20.18 (17.31, 23.04) | 41.11 (35.02, 47.37) | 103.74 | 640.72 (550.04, 732.16) | 641.05 (550.88, 735.26) | 0.02 (-0.01, 0.05) |
| Botswana | 2.24 (1.9, 2.57) | 6.05 (5.16, 6.92) | 170.57 | 622.87 (536.42, 712.36) | 597.43 (511.15, 685.77) | -0.1 (-0.13, -0.08) |
| Brazil | 526.4 (455.69, 602.29) | 1825.79 (1586.09, 2075.17) | 246.85 | 759.73 (661.49, 864.32) | 761.45 (661.47, 869.62) | -0.05 (-0.09, -0.01) |
| Brunei Darussalam | 0.44 (0.37, 0.5) | 1.33 (1.14, 1.52) | 205.64 | 581.28 (494.56, 670) | 578.96 (496.58, 663.75) | 0.02 (-0.02, 0.05) |
| Bulgaria | 66.3 (55.63, 77.45) | 98.85 (84.43, 114.51) | 49.09 | 657.85 (564.21, 753.61) | 643.45 (553.32, 739.68) | -0.08 (-0.11, -0.06) |
| Burkina Faso | 14.69 (12.61, 16.78) | 30.52 (26.15, 34.75) | 107.78 | 480.35 (414.61, 547.47) | 447.82 (385.98, 510.39) | -0.25 (-0.25, -0.24) |
| Burundi | 10.64 (9.12, 12.22) | 18.57 (16.04, 21.06) | 74.6 | 632.52 (546.31, 717.71) | 580.93 (501.14, 662.01) | -0.25 (-0.27, -0.23) |
| Cabo Verde | 1.12 (0.96, 1.28) | 1.8 (1.54, 2.04) | 60.62 | 465.75 (399.93, 529.12) | 440.96 (378.26, 500.17) | -0.18 (-0.19, -0.17) |
| Cambodia | 20.92 (17.8, 23.84) | 59.41 (50.61, 68.05) | 184.03 | 683.19 (589.81, 782.26) | 658.61 (568.01, 754.43) | -0.17 (-0.19, -0.15) |
| Cameroon | 13.89 (11.93, 15.88) | 36.25 (31.04, 41.43) | 160.95 | 445.65 (383.06, 507.77) | 421.43 (362.09, 481.18) | -0.18 (-0.18, -0.17) |
| Canada | 285.21 (250.85, 320.42) | 631.35 (556.98, 707.71) | 121.36 | 885.63 (781.67, 993.27) | 792.31 (701.61, 888.19) | -0.55 (-0.64, -0.46) |
| Central African Republic | 4.96 (4.26, 5.66) | 9.27 (7.99, 10.58) | 86.83 | 800 (688.59, 906.05) | 795.18 (681.53, 900.67) | -0.1 (-0.13, -0.08) |
| Chad | 10.63 (9.04, 12.1) | 17.24 (14.83, 19.5) | 62.25 | 472.92 (405.5, 537.32) | 428.14 (366.66, 486.61) | -0.33 (-0.34, -0.32) |
| Chile | 53.09 (46.34, 60.55) | 159.05 (137.15, 182.06) | 199.57 | 611.01 (533.28, 694.64) | 601.08 (518.53, 687.09) | -0.02 (-0.04, 0.01) |
| China | 4024.54 (3446.4, 4623.09) | 16990.83 (14488.49, 19672.74) | 322.18 | 703.14 (608.36, 809.51) | 900.82 (770.92, 1043.22) | 0.44 (0.36, 0.52) |
| Colombia | 91.16 (78.65, 103.31) | 362.96 (313.43, 410.68) | 298.14 | 647.46 (559.46, 736.84) | 638.88 (549.98, 725.16) | -0.03 (-0.06, -0.01) |
| Comoros | 0.8 (0.68, 0.91) | 2.19 (1.88, 2.48) | 174.82 | 611.5 (525.54, 694.46) | 582.1 (503.27, 661.45) | -0.14 (-0.15, -0.13) |
| Congo | 4.91 (4.19, 5.63) | 11.81 (10.54, 13) | 140.59 | 742.92 (641.57, 845.44) | 706.08 (641.29, 770.22) | -0.14 (-0.15, -0.12) |
| Cook Islands | 0.06 (0.05, 0.07) | 0.16 (0.14, 0.19) | 163.51 | 644.33 (544.78, 747.85) | 631.64 (535.44, 728.65) | -0.08 (-0.09, -0.07) |
| Costa Rica | 10.29 (8.9, 11.76) | 35.07 (30.51, 39.92) | 240.84 | 646.87 (556.77, 739.05) | 631.1 (547.39, 722.93) | -0.07 (-0.09, -0.05) |
| Cote d'Ivoire | 10.63 (9.1, 12.13) | 31.52 (27.19, 36.06) | 196.54 | 446.24 (385.24, 513.22) | 428.76 (367.98, 489.19) | -0.13 (-0.15, -0.11) |
| Croatia | 35.2 (29.95, 40.58) | 65.21 (55.71, 75.36) | 85.26 | 671.05 (576.4, 770.36) | 647.44 (556.18, 745.36) | -0.09 (-0.1, -0.08) |
| Cuba | 51.19 (44.09, 58.48) | 111.07 (96.37, 125.73) | 116.99 | 521.16 (453.79, 590.27) | 526.06 (456.06, 596.36) | -0.19 (-0.24, -0.13) |
| Cyprus | 4.49 (3.75, 5.28) | 13.69 (11.6, 15.89) | 205.28 | 690.13 (589.98, 796.69) | 661.56 (568.54, 762.23) | -0.09 (-0.11, -0.07) |
| Czechia | 86.22 (73.39, 99.82) | 150.65 (129.27, 172.47) | 74.73 | 643.53 (550.56, 735.12) | 638.18 (547.88, 727.68) | -0.06 (-0.07, -0.05) |
| Democratic People's Republic of Korea | 69.72 (59.1, 80.2) | 177.07 (151.29, 204.07) | 153.98 | 631.91 (537.88, 725.98) | 618.92 (529.05, 710.41) | -0.08 (-0.11, -0.06) |
| Democratic Republic of the Congo | 69.18 (59.28, 79.14) | 172.19 (149.6, 195.62) | 148.89 | 746.9 (646.06, 847.11) | 755.89 (656.94, 858.4) | 0.05 (0.02, 0.09) |
| Denmark | 52.76 (45.92, 60.38) | 64.49 (55.73, 73.64) | 22.23 | 586.93 (511.27, 666.79) | 474.71 (409.31, 539.52) | -0.78 (-0.82, -0.73) |
| Djibouti | 0.49 (0.42, 0.55) | 2.3 (1.98, 2.62) | 372.72 | 638.22 (547.66, 724.77) | 603.12 (517.99, 686.84) | -0.14 (-0.16, -0.13) |
| Dominica | 0.33 (0.29, 0.38) | 0.42 (0.36, 0.48) | 27.28 | 569.19 (493.8, 648.33) | 552.34 (475.16, 630.23) | -0.09 (-0.1, -0.08) |
| Dominican Republic | 17 (14.78, 19.37) | 54.45 (46.95, 62) | 220.32 | 566.19 (491.01, 645.07) | 572.64 (494.88, 653) | -0.11 (-0.15, -0.07) |
| Ecuador | 21.19 (18.32, 24.23) | 70.16 (59.86, 80.09) | 231.04 | 466.53 (403.61, 534.38) | 455.05 (388.62, 521.67) | -0.04 (-0.05, -0.02) |
| Egypt | 126.46 (110.29, 141.56) | 283.12 (244.75, 318.07) | 123.88 | 754.52 (663.1, 848.65) | 726.9 (630.54, 823.49) | -0.06 (-0.08, -0.03) |
| El Salvador | 17.37 (15.12, 19.75) | 43.35 (37.71, 49.58) | 149.48 | 630.69 (545.57, 718.39) | 637.92 (552.58, 726.95) | 0.07 (0.05, 0.08) |
| Equatorial Guinea | 0.93 (0.8, 1.07) | 2.47 (2.11, 2.8) | 164.95 | 771.03 (667.01, 877.69) | 736.91 (636.47, 836.63) | -0.17 (-0.18, -0.16) |
| Eritrea | 3.5 (3, 3.99) | 9.86 (8.42, 11.26) | 181.8 | 642.78 (555.48, 731.56) | 602.42 (518.16, 687.76) | -0.2 (-0.2, -0.19) |
| Estonia | 13.05 (11.09, 15.06) | 21.19 (18.14, 24.37) | 62.39 | 657.29 (561.79, 755.68) | 648.82 (559.06, 741.98) | -0.01 (-0.06, 0.03) |
| Eswatini | 1.15 (0.99, 1.31) | 2.04 (1.75, 2.33) | 76.78 | 604.07 (520.51, 691.04) | 579.24 (499.45, 661.83) | -0.14 (-0.16, -0.13) |
| Ethiopia | 74.98 (64.19, 86.07) | 194.3 (168.32, 220.69) | 159.14 | 638.14 (554, 727.32) | 586.55 (509.54, 668.44) | -0.24 (-0.28, -0.21) |
| Fiji | 1.44 (1.21, 1.66) | 3.31 (2.8, 3.82) | 130.47 | 650.89 (555.43, 752.89) | 637.89 (544.12, 735.6) | -0.09 (-0.1, -0.07) |
| Finland | 50.59 (43.27, 57.56) | 95.44 (80.33, 109.9) | 88.66 | 690.51 (593.57, 781.35) | 616.9 (523.13, 709.76) | -0.4 (-0.41, -0.39) |
| France | 511.5 (451.63, 568.21) | 924.61 (807.08, 1045.11) | 80.76 | 553.5 (491.88, 615.65) | 525.22 (458.8, 593.27) | -0.2 (-0.23, -0.16) |
| Gabon | 3.34 (2.85, 3.8) | 5.31 (4.56, 6.05) | 58.98 | 753.38 (649.45, 860.8) | 734.43 (633.02, 837.16) | -0.05 (-0.06, -0.04) |
| Gambia | 1.12 (0.96, 1.28) | 3.22 (2.77, 3.68) | 187.09 | 473.34 (405.47, 538.3) | 436.83 (374.25, 499.13) | -0.27 (-0.28, -0.26) |
| Georgia | 36.65 (31.6, 41.68) | 43.61 (37.83, 49.55) | 19 | 651.05 (562.33, 741.66) | 651.46 (562.89, 743.97) | 0.01 (0, 0.03) |
| Germany | 1093.58 (962.01, 1237.64) | 1995.25 (1724.73, 2268.19) | 82.45 | 809.57 (715.41, 909.95) | 820.5 (713.82, 930.77) | 0.06 (0.03, 0.08) |
| Ghana | 18 (15.48, 20.68) | 48.62 (41.4, 55.39) | 170.09 | 429.93 (369, 491.63) | 419.76 (360.59, 476.55) | -0.06 (-0.07, -0.05) |
| Greece | 105.42 (89.91, 122.07) | 212.41 (181.05, 246.25) | 101.48 | 703.66 (602.22, 809.6) | 671.46 (575.61, 767.68) | -0.13 (-0.15, -0.11) |
| Greenland | 0.17 (0.14, 0.19) | 0.4 (0.34, 0.46) | 137.3 | 805.34 (683.87, 935.07) | 786.97 (673.97, 906.26) | -0.09 (-0.11, -0.06) |
| Grenada | 0.48 (0.41, 0.55) | 0.52 (0.45, 0.6) | 8.54 | 570.58 (491.2, 651.7) | 562.63 (487.52, 643.75) | -0.02 (-0.05, 0.01) |
| Guam | 0.31 (0.26, 0.35) | 1.41 (1.21, 1.62) | 361.86 | 632.58 (536.4, 731.64) | 629.81 (535.65, 725.95) | -0.03 (-0.04, -0.02) |
| Guatemala | 15.48 (13.37, 17.56) | 61.94 (53.27, 70.94) | 300.08 | 646.74 (561.57, 736.53) | 639.86 (553.28, 730.62) | -0.02 (-0.03, 0) |
| Guinea | 12.34 (10.74, 14.03) | 18.99 (16.33, 21.58) | 53.93 | 466.54 (403.27, 530.43) | 438.63 (379.03, 498.93) | -0.2 (-0.22, -0.18) |
| Guinea-Bissau | 1.12 (0.96, 1.29) | 1.87 (1.6, 2.14) | 66.04 | 443.86 (380.49, 503.71) | 431.13 (370.44, 493.27) | -0.08 (-0.09, -0.07) |
| Guyana | 1.64 (1.4, 1.87) | 2.73 (2.33, 3.1) | 66.66 | 554.65 (477.24, 635) | 549.03 (471.54, 629.36) | -0.04 (-0.05, -0.03) |
| Haiti | 12.45 (10.64, 14.14) | 26.29 (22.59, 30.02) | 111.07 | 586.32 (506.12, 662.48) | 541.86 (468.24, 619.7) | -0.28 (-0.29, -0.26) |
| Honduras | 10.4 (8.96, 11.82) | 31.86 (27.16, 36.51) | 206.47 | 653.72 (564.16, 740.88) | 641.67 (553.16, 730.68) | -0.06 (-0.07, -0.06) |
| Hungary | 89.41 (76.16, 102.78) | 139.42 (119.7, 159.96) | 55.93 | 645.34 (551.33, 739.87) | 638.1 (548.27, 732.18) | -0.02 (-0.06, 0.02) |
| Iceland | 2.35 (2.02, 2.66) | 4.53 (3.89, 5.1) | 92.76 | 761 (654.06, 859.57) | 683.68 (586.64, 767.04) | -0.4 (-0.42, -0.38) |
| India | 1350.13 (1162.81, 1543.95) | 4169.68 (3591.61, 4772.66) | 208.84 | 440.05 (382.11, 503.9) | 436.13 (376.02, 501.15) | -0.16 (-0.21, -0.12) |
| Indonesia | 460.74 (396.35, 528.84) | 1109.36 (953.36, 1273.17) | 140.78 | 678.72 (587.97, 777.61) | 662.13 (571.11, 761.15) | -0.08 (-0.1, -0.05) |
| Iran (Islamic Republic of) | 133.81 (115.41, 152.67) | 506.63 (438.93, 578.9) | 278.62 | 804.38 (692.92, 921.7) | 775.51 (671.84, 887.85) | -0.11 (-0.12, -0.1) |
| Iraq | 55.21 (47.18, 63.2) | 126.35 (108.2, 144.6) | 128.86 | 796.72 (678.19, 914.39) | 759.67 (652.95, 868.12) | -0.19 (-0.2, -0.18) |
| Ireland | 27.68 (23.5, 32.02) | 53.86 (45.78, 62.14) | 94.59 | 691.14 (590.07, 791.51) | 629.49 (535.55, 725.24) | -0.31 (-0.34, -0.29) |
| Israel | 31.75 (27, 36.96) | 87.01 (74.41, 100.6) | 174.08 | 678.13 (581.61, 781.72) | 636.97 (546.32, 734.42) | -0.21 (-0.22, -0.21) |
| Italy | 633.48 (535.32, 736.35) | 1427.95 (1213.98, 1647.06) | 125.41 | 692.96 (589.47, 798.14) | 759.15 (652.54, 869.83) | 0.13 (-0.01, 0.27) |
| Jamaica | 12.08 (10.37, 13.75) | 20.33 (17.53, 23.07) | 68.29 | 643.37 (553.27, 732.56) | 609.75 (523.06, 695.65) | -0.2 (-0.21, -0.18) |
| Japan | 1023.23 (885.71, 1169.18) | 3367.36 (2893.95, 3872.08) | 229.09 | 649.68 (564.43, 739.33) | 674.1 (584.78, 770.24) | 0.3 (0.25, 0.36) |
| Jordan | 6.99 (6, 7.97) | 41.15 (35.43, 46.86) | 488.82 | 784.45 (675.13, 901.07) | 786.72 (675.7, 906.66) | 0.05 (0.01, 0.09) |
| Kazakhstan | 67.95 (58.57, 77.8) | 87.91 (75.73, 100.52) | 29.37 | 649.97 (561.89, 746.48) | 634.04 (542.82, 724.42) | -0.08 (-0.11, -0.06) |
| Kenya | 37.54 (32.43, 42.75) | 94.64 (81.57, 107.74) | 152.14 | 613.02 (529.32, 697.76) | 605.07 (525.16, 689.45) | -0.04 (-0.07, -0.02) |
| Kiribati | 0.16 (0.13, 0.18) | 0.31 (0.26, 0.35) | 92.27 | 699.11 (598.21, 802.88) | 701.89 (602.78, 803.92) | 0 (-0.03, 0.04) |
| Kuwait | 3.12 (2.7, 3.51) | 16.14 (14.06, 18.14) | 416.8 | 820.13 (709.23, 935.64) | 771.61 (668.04, 878.03) | -0.2 (-0.22, -0.17) |
| Kyrgyzstan | 16.68 (14.4, 18.92) | 23.64 (20.39, 26.81) | 41.75 | 645.42 (559.15, 733.86) | 649.99 (562.04, 741.22) | 0.05 (0.04, 0.06) |
| Lao People's Democratic Republic | 9.18 (7.78, 10.58) | 21.89 (18.99, 24.98) | 138.42 | 674.31 (585.55, 768.35) | 655.85 (567.01, 749.46) | -0.07 (-0.09, -0.06) |
| Latvia | 23.31 (19.91, 26.94) | 31.57 (27.02, 36.44) | 35.44 | 659.29 (565.04, 757.79) | 660.59 (570.4, 758.14) | 0.04 (0.02, 0.06) |
| Lebanon | 13.98 (12.02, 15.95) | 54.35 (46.45, 62.27) | 288.76 | 826.34 (713.64, 946.49) | 828.25 (710.4, 948.14) | -0.02 (-0.03, -0.01) |
| Lesotho | 4.22 (3.62, 4.79) | 4.51 (3.86, 5.11) | 6.79 | 636.5 (551.08, 723.89) | 625.49 (537.97, 713.46) | -0.03 (-0.04, -0.02) |
| Liberia | 3.79 (3.23, 4.34) | 6.14 (5.31, 6.97) | 62.13 | 435.63 (377.36, 494.91) | 419.94 (360.58, 478.03) | -0.12 (-0.13, -0.11) |
| Libya | 12.54 (10.9, 14.16) | 30.32 (26.22, 34.4) | 141.72 | 805.14 (695.68, 912.97) | 762.64 (660.37, 872.63) | -0.18 (-0.19, -0.16) |
| Lithuania | 29.31 (25.04, 33.96) | 45 (38.72, 51.8) | 53.51 | 650.56 (561.65, 749.04) | 648.52 (560.43, 740.2) | 0.02 (0, 0.05) |
| Luxembourg | 3.04 (2.57, 3.53) | 5.61 (4.76, 6.52) | 84.72 | 551.09 (468.86, 636.23) | 473.86 (402.43, 551.8) | -0.53 (-0.6, -0.47) |
| Madagascar | 21.75 (18.7, 24.76) | 38.47 (33.09, 44.11) | 76.89 | 606.36 (524.14, 687.06) | 574.95 (493.29, 652.02) | -0.16 (-0.17, -0.14) |
| Malawi | 15.37 (13.13, 17.52) | 30.19 (25.79, 34.28) | 96.35 | 607.88 (526.91, 694.55) | 605.24 (517.75, 689.89) | 0 (-0.01, 0.02) |
| Malaysia | 53.82 (46.76, 60.83) | 153.81 (130.89, 175.27) | 185.8 | 695.54 (601.08, 791.73) | 657.55 (561.66, 750.29) | -0.13 (-0.16, -0.11) |
| Maldives | 0.37 (0.31, 0.42) | 1.79 (1.54, 2.06) | 388.94 | 647.37 (556.97, 738.29) | 667.3 (572.03, 764.23) | 0.1 (0.07, 0.13) |
| Mali | 12.16 (10.4, 13.83) | 26.75 (23.2, 30.49) | 119.89 | 460.8 (396.23, 525.5) | 439.59 (379.27, 499.83) | -0.15 (-0.15, -0.14) |
| Malta | 2.73 (2.33, 3.16) | 7.3 (6.25, 8.44) | 167.87 | 695.45 (597.76, 804.83) | 642.06 (551.42, 738.48) | -0.24 (-0.27, -0.21) |
| Marshall Islands | 0.07 (0.06, 0.08) | 0.12 (0.1, 0.13) | 75.77 | 617.4 (523.92, 711.85) | 594.07 (502.31, 685.62) | -0.13 (-0.13, -0.12) |
| Mauritania | 3.56 (3.03, 4.08) | 7.42 (6.37, 8.44) | 108.59 | 467.98 (400.78, 533.61) | 433.09 (374.33, 493.21) | -0.25 (-0.26, -0.24) |
| Mauritius | 3.62 (3.1, 4.19) | 11.14 (9.55, 12.62) | 207.28 | 663.44 (568.33, 762.47) | 657.17 (567.03, 748.3) | 0 (-0.02, 0.03) |
| Mexico | 200.52 (172.13, 230.33) | 608.49 (526.1, 699.6) | 203.46 | 585.32 (506.17, 669.22) | 534.2 (460.41, 612.87) | -0.2 (-0.23, -0.16) |
| Micronesia (Federated States of) | 0.24 (0.21, 0.28) | 0.32 (0.27, 0.37) | 31.42 | 691.53 (590.09, 798.37) | 695.38 (594.38, 799.43) | 0.05 (0.04, 0.06) |
| Monaco | 0.59 (0.5, 0.69) | 0.77 (0.66, 0.89) | 30.3 | 696.22 (595.18, 799.43) | 639.25 (542.47, 742.18) | -0.3 (-0.3, -0.29) |
| Mongolia | 5.38 (4.65, 6.15) | 10.28 (8.91, 11.61) | 90.98 | 653.39 (565.27, 748.42) | 660.02 (573.53, 749.23) | 0.05 (0.04, 0.06) |
| Montenegro | 3.75 (3.25, 4.29) | 5.76 (4.89, 6.61) | 53.59 | 656.91 (570.25, 753.22) | 639.32 (547.61, 729.15) | -0.13 (-0.17, -0.09) |
| Morocco | 93.82 (81.21, 106.44) | 210.67 (180.86, 239.5) | 124.55 | 804.49 (696.28, 914.48) | 748.52 (644.54, 856.83) | -0.24 (-0.25, -0.23) |
| Mozambique | 24.88 (21.22, 28.32) | 43.03 (36.96, 48.76) | 72.98 | 624.73 (540.32, 711.49) | 605.09 (520.43, 691.27) | -0.08 (-0.1, -0.06) |
| Myanmar | 115.86 (99.6, 131.75) | 257.64 (222.04, 292.33) | 122.38 | 720.54 (625.38, 824.31) | 663.53 (571.48, 757.76) | -0.28 (-0.29, -0.26) |
| Namibia | 2.6 (2.19, 2.95) | 5.77 (5, 6.52) | 122.04 | 616.32 (530.85, 701.68) | 593.68 (514.23, 676.82) | -0.11 (-0.12, -0.1) |
| Nauru | 0.02 (0.01, 0.02) | 0.02 (0.02, 0.03) | 42.1 | 632.73 (535.53, 733.18) | 652.63 (555.94, 749.51) | 0.07 (0.03, 0.12) |
| Nepal | 31.93 (27.61, 36.15) | 83.34 (71.56, 95.05) | 161.01 | 510.81 (441.59, 585.55) | 454.19 (392.41, 519.26) | -0.46 (-0.49, -0.43) |
| Netherlands | 148.61 (130.05, 165.2) | 278.82 (242.06, 316.18) | 87.62 | 711.18 (625.56, 787.39) | 700.28 (610.56, 794.56) | 0.01 (-0.03, 0.04) |
| New Zealand | 27.94 (23.98, 32.14) | 62.91 (54.14, 72.38) | 125.19 | 728.11 (627.47, 840.73) | 684.59 (589.02, 787.91) | -0.24 (-0.29, -0.2) |
| Nicaragua | 8.19 (7.1, 9.23) | 27.8 (24.2, 31.75) | 239.34 | 660.24 (569.18, 749.74) | 654.85 (569.17, 748.6) | 0 (-0.01, 0.01) |
| Niger | 8.16 (7, 9.33) | 24.49 (20.96, 27.91) | 200.05 | 470.52 (404.38, 536.04) | 439.52 (380.44, 501.25) | -0.22 (-0.23, -0.21) |
| Nigeria | 139.8 (119.31, 159.12) | 253.56 (218.39, 289.16) | 81.37 | 417.35 (360.95, 476.61) | 377.62 (326.26, 432.75) | -0.35 (-0.38, -0.31) |
| Niue | 0.02 (0.01, 0.02) | 0.01 (0.01, 0.01) | -20.9 | 635.45 (538.72, 734.68) | 616.47 (519.99, 710.42) | -0.13 (-0.14, -0.11) |
| North Macedonia | 10.03 (8.6, 11.4) | 17.23 (14.56, 19.88) | 71.69 | 641.1 (548.24, 734.12) | 636.56 (546.06, 726.34) | -0.01 (-0.02, 0.01) |
| Northern Mariana Islands | 0.06 (0.05, 0.06) | 0.22 (0.18, 0.25) | 292.43 | 630.79 (530.47, 734.74) | 621.13 (528.08, 721.28) | -0.04 (-0.06, -0.03) |
| Norway | 59.57 (51.41, 68.43) | 73.85 (63.75, 85.24) | 23.98 | 769.17 (664.98, 878.38) | 639.18 (549.63, 735.45) | -0.67 (-0.72, -0.63) |
| Oman | 3.63 (3.11, 4.17) | 9.01 (7.68, 10.32) | 148.08 | 787.34 (678.3, 903.05) | 728.25 (624.77, 840.49) | -0.24 (-0.28, -0.19) |
| Pakistan | 205.55 (175.76, 235.97) | 375.36 (321.68, 430) | 82.61 | 464.81 (400.97, 530.52) | 433.58 (374.32, 499.28) | -0.25 (-0.26, -0.24) |
| Palau | 0.04 (0.04, 0.05) | 0.09 (0.08, 0.1) | 109.1 | 616.08 (520.12, 716.46) | 594.28 (501.85, 687.51) | -0.11 (-0.12, -0.1) |
| Palestine | 5.56 (4.77, 6.31) | 13.75 (11.88, 15.66) | 147.36 | 803.58 (690.88, 915.33) | 773.83 (669.58, 881.66) | -0.14 (-0.16, -0.11) |
| Panama | 8.35 (7.26, 9.49) | 28.21 (24.27, 32.08) | 237.8 | 628.65 (543.24, 715.98) | 620.46 (530.21, 708.4) | -0.05 (-0.05, -0.04) |
| Papua New Guinea | 6.96 (5.93, 8.03) | 19.75 (16.87, 22.46) | 183.69 | 693.65 (602.01, 797.17) | 649.65 (557.31, 742.38) | -0.26 (-0.29, -0.24) |
| Paraguay | 14.31 (12.37, 16.18) | 36.3 (31.3, 41.13) | 153.64 | 729.89 (629.4, 832.72) | 687.12 (593.84, 779.77) | -0.22 (-0.23, -0.21) |
| Peru | 46.44 (40.35, 53.07) | 144.35 (125.3, 165.28) | 210.79 | 441.69 (382.66, 505.23) | 436.46 (377.5, 501.78) | -0.09 (-0.11, -0.07) |
| Philippines | 148.62 (127.91, 170.25) | 419.36 (364.5, 477.88) | 182.18 | 691.99 (601.34, 792.19) | 662.94 (575.83, 762.08) | -0.17 (-0.2, -0.15) |
| Poland | 274.98 (236.32, 317.72) | 501.49 (432.86, 576.74) | 82.37 | 683.36 (589.12, 787.44) | 645.55 (558.48, 741.84) | -0.19 (-0.2, -0.18) |
| Portugal | 87.61 (74.6, 101.09) | 203.14 (171.37, 235.89) | 131.85 | 672.6 (577.21, 772.67) | 659.36 (561.71, 757.5) | -0.04 (-0.06, -0.02) |
| Puerto Rico | 20.23 (17.33, 23.2) | 50.55 (43.48, 58) | 149.89 | 576.17 (496.29, 659.73) | 563.62 (484.51, 646.62) | -0.08 (-0.09, -0.07) |
| Qatar | 0.41 (0.35, 0.46) | 3.43 (2.94, 3.89) | 740.31 | 761.25 (655.07, 876.05) | 740.91 (636.99, 852.34) | -0.02 (-0.06, 0.02) |
| Republic of Korea | 149.62 (129.87, 168.61) | 697.05 (609.57, 789.74) | 365.87 | 751.01 (651.71, 856.59) | 739.81 (647.7, 836.31) | 0.01 (-0.04, 0.06) |
| Republic of Moldova | 22 (18.81, 25.08) | 39.09 (33.37, 44.62) | 77.66 | 633.94 (547.62, 724.01) | 640.52 (549.24, 730.71) | 0.03 (0.01, 0.05) |
| Romania | 150.69 (127.97, 173.36) | 265.11 (226.93, 304.83) | 75.93 | 638.6 (547.75, 734.41) | 637.2 (547.1, 729.32) | -0.04 (-0.05, -0.02) |
| Russian Federation | 1044.4 (900.32, 1202.42) | 1605.86 (1392.75, 1846.54) | 53.76 | 669.98 (580.01, 769.14) | 661.42 (573.18, 758.61) | -0.08 (-0.13, -0.04) |
| Rwanda | 11.09 (9.51, 12.65) | 26.43 (22.78, 29.9) | 138.42 | 619.83 (537.09, 704.91) | 616.11 (532.05, 699.77) | 0.04 (0.02, 0.06) |
| Saint Kitts and Nevis | 0.2 (0.17, 0.23) | 0.28 (0.24, 0.32) | 39.57 | 540.25 (465.78, 620.82) | 534.03 (461.67, 613.08) | -0.05 (-0.06, -0.04) |
| Saint Lucia | 0.44 (0.38, 0.51) | 1.28 (1.1, 1.47) | 189.15 | 568.49 (488.1, 652.02) | 551.55 (473.03, 632.04) | -0.13 (-0.15, -0.1) |
| Saint Vincent and the Grenadines | 0.38 (0.33, 0.44) | 0.73 (0.63, 0.84) | 90.87 | 583.48 (499.61, 667.35) | 553.95 (476.99, 632.63) | -0.16 (-0.17, -0.16) |
| Samoa | 0.4 (0.34, 0.46) | 0.71 (0.6, 0.82) | 76.23 | 643.9 (547.03, 740.91) | 622.74 (531.09, 716.31) | -0.13 (-0.14, -0.12) |
| San Marino | 0.27 (0.23, 0.31) | 0.6 (0.52, 0.7) | 126.42 | 704.99 (610.5, 806.61) | 623.02 (535.54, 718.97) | -0.41 (-0.43, -0.39) |
| Sao Tome and Principe | 0.24 (0.21, 0.27) | 0.34 (0.29, 0.39) | 41.18 | 442.32 (381.71, 502.56) | 410.94 (351.44, 470.85) | -0.23 (-0.25, -0.21) |
| Saudi Arabia | 30.76 (26.36, 35.09) | 71.83 (61.58, 82.12) | 133.56 | 747.42 (639.76, 857.11) | 701.74 (600.97, 807.91) | -0.2 (-0.21, -0.19) |
| Senegal | 11.11 (9.55, 12.69) | 25.54 (21.9, 29.1) | 129.93 | 462.03 (395.41, 527.14) | 429.79 (370.45, 490.06) | -0.26 (-0.27, -0.25) |
| Serbia | 55.54 (47.22, 63.83) | 111.69 (95.44, 128.38) | 101.09 | 636.43 (544.83, 728.62) | 638.51 (545.56, 731.1) | -0.01 (-0.02, 0.01) |
| Seychelles | 0.37 (0.32, 0.43) | 0.64 (0.54, 0.73) | 71.09 | 667.93 (571.7, 769.28) | 643.49 (544.72, 741.31) | -0.14 (-0.14, -0.13) |
| Sierra Leone | 7.68 (6.58, 8.77) | 12.38 (10.65, 14.09) | 61.16 | 469.47 (403.35, 535.67) | 442.53 (380.65, 506.69) | -0.19 (-0.2, -0.18) |
| Singapore | 8.79 (7.52, 9.95) | 43.79 (39, 48.43) | 398.4 | 516.08 (443.63, 583.07) | 534.49 (474.45, 589.82) | 0.11 (0.07, 0.15) |
| Slovakia | 36.5 (31.16, 41.74) | 60.99 (52.45, 70.4) | 67.09 | 646.5 (555.59, 735.34) | 635.02 (547.19, 728.34) | -0.06 (-0.07, -0.05) |
| Slovenia | 15.35 (13.13, 17.64) | 33.19 (28.4, 38.24) | 116.23 | 634.22 (546.7, 725.16) | 635.78 (543.67, 726.69) | 0.05 (0.02, 0.08) |
| Solomon Islands | 0.51 (0.43, 0.59) | 1.44 (1.22, 1.65) | 181.6 | 654.61 (557.57, 751.56) | 652.57 (555.17, 753.61) | 0.03 (0.01, 0.06) |
| Somalia | 8.33 (7.18, 9.44) | 20.26 (17.34, 23.14) | 143.38 | 623.18 (537.91, 706.76) | 617.03 (535.75, 699.67) | -0.01 (-0.02, 0) |
| South Africa | 109.92 (94.75, 125.48) | 224.95 (193.26, 257.57) | 104.66 | 646.32 (559.39, 738.74) | 609.51 (526.77, 695.77) | -0.16 (-0.17, -0.15) |
| South Sudan | 11.49 (9.85, 13.15) | 14.64 (12.58, 16.62) | 27.4 | 616.16 (531.63, 702.53) | 572.38 (489.94, 650.54) | -0.23 (-0.26, -0.2) |
| Spain | 373.39 (324.69, 413.76) | 713.87 (618.88, 810.22) | 91.19 | 676.37 (589.33, 747.58) | 582.15 (504.96, 664.35) | -0.37 (-0.44, -0.3) |
| Sri Lanka | 53.75 (45.88, 61.24) | 152.89 (129.86, 175.78) | 184.43 | 658.88 (567.42, 750.44) | 634.13 (545.74, 727.67) | -0.13 (-0.15, -0.12) |
| Sudan | 53.97 (46.23, 61.82) | 105.44 (91.15, 119.88) | 95.35 | 790.96 (686.08, 900.75) | 732.33 (633.47, 834.54) | -0.26 (-0.27, -0.25) |
| Suriname | 1.33 (1.14, 1.51) | 3.32 (2.87, 3.78) | 149.35 | 602.96 (519.13, 689.29) | 574.18 (495.68, 655.29) | -0.17 (-0.19, -0.16) |
| Sweden | 130.52 (112.97, 149.46) | 185.34 (159.94, 212.19) | 42 | 755.36 (656.33, 856.34) | 697.2 (600.37, 799.05) | -0.18 (-0.22, -0.13) |
| Switzerland | 83.49 (71.52, 96.12) | 141.61 (121.47, 164.12) | 69.61 | 715.63 (614.44, 820.18) | 639.88 (549.35, 735.13) | -0.39 (-0.42, -0.37) |
| Syrian Arab Republic | 32.57 (28.24, 36.9) | 74.18 (63.56, 84.73) | 127.77 | 819.88 (707.35, 932.52) | 753.82 (648.01, 860.72) | -0.28 (-0.29, -0.27) |
| Taiwan (Province of China) | 57.3 (49.47, 65.77) | 246.28 (209.4, 276.22) | 329.8 | 507.64 (438.25, 581.48) | 555.31 (472.14, 624.76) | 0.46 (0.35, 0.57) |
| Tajikistan | 14.98 (12.94, 16.99) | 23.73 (20.34, 27.18) | 58.4 | 638.14 (552.15, 725.33) | 597.35 (515.42, 684.84) | -0.23 (-0.24, -0.22) |
| Thailand | 162.94 (140.91, 183.45) | 659.44 (571.37, 751.94) | 304.71 | 601.34 (522.75, 679.65) | 603.22 (522.58, 689.6) | 0.15 (0.08, 0.21) |
| Timor-Leste | 1.23 (1.07, 1.41) | 4.51 (3.82, 5.18) | 265.06 | 711.62 (616.21, 810.55) | 664.45 (572.75, 759.71) | -0.21 (-0.23, -0.18) |
| Togo | 3.8 (3.28, 4.33) | 10.75 (9.19, 12.32) | 183.25 | 454.23 (390.67, 518.47) | 438.21 (378.97, 499.37) | -0.1 (-0.11, -0.09) |
| Tokelau | 0.01 (0.01, 0.01) | 0.01 (0.01, 0.01) | 23.67 | 640.34 (542.8, 734.01) | 626.8 (531.25, 725.34) | -0.07 (-0.08, -0.06) |
| Tonga | 0.28 (0.24, 0.32) | 0.48 (0.41, 0.55) | 71.23 | 665.84 (572.95, 764.8) | 644.39 (551.18, 740.15) | -0.12 (-0.13, -0.1) |
| Trinidad and Tobago | 4.19 (3.58, 4.82) | 10.4 (8.93, 11.85) | 148.19 | 578.04 (497.45, 659.1) | 564.63 (486.16, 644.68) | -0.06 (-0.07, -0.06) |
| Tunisia | 31.48 (27.09, 35.96) | 92.44 (80.3, 104.68) | 193.68 | 846.86 (730.77, 961.13) | 791.09 (685.56, 901.07) | -0.21 (-0.22, -0.2) |
| Turkey | 235.49 (203.4, 268.35) | 688.43 (595.24, 787.22) | 192.33 | 873.29 (753.94, 992.75) | 819.36 (706.2, 936.59) | -0.25 (-0.27, -0.23) |
| Turkmenistan | 9.34 (8.03, 10.59) | 18.83 (16.19, 21.36) | 101.64 | 641.51 (553.07, 728.88) | 609.86 (523.2, 698.24) | -0.18 (-0.21, -0.15) |
| Tuvalu | 0.03 (0.02, 0.03) | 0.05 (0.04, 0.06) | 83.65 | 668.63 (572.05, 767.56) | 650.7 (560.53, 748.55) | -0.09 (-0.1, -0.07) |
| Uganda | 27.59 (23.68, 31.41) | 61.43 (52.96, 69.59) | 122.63 | 601.84 (519.22, 682.95) | 594.04 (508.15, 676.31) | -0.05 (-0.07, -0.02) |
| Ukraine | 438.83 (373.96, 512.46) | 527.24 (453.77, 609.73) | 20.15 | 672.2 (576.91, 781.63) | 651.18 (564.01, 749.5) | -0.12 (-0.16, -0.08) |
| United Arab Emirates | 1.7 (1.45, 1.94) | 11.96 (9.94, 14.03) | 605.37 | 723.88 (612.89, 834.43) | 652.43 (552.16, 753.8) | -0.33 (-0.37, -0.29) |
| United Kingdom | 607.85 (521.26, 696.58) | 906.47 (778.22, 1036.14) | 49.13 | 625.6 (539.95, 712.3) | 591.08 (508.13, 674.77) | -0.13 (-0.17, -0.09) |
| United Republic of Tanzania | 46.62 (40.19, 53.14) | 108.49 (95.56, 120.85) | 132.71 | 620.11 (533.93, 703.93) | 569.9 (503.41, 636.97) | -0.25 (-0.27, -0.24) |
| United States of America | 2736.35 (2365.02, 3137.76) | 4876.58 (4231.62, 5601.21) | 78.21 | 809.27 (700.87, 926.57) | 773.07 (670.26, 887.66) | -0.17 (-0.19, -0.16) |
| United States Virgin Islands | 0.36 (0.31, 0.42) | 1.04 (0.89, 1.22) | 189.19 | 560.83 (481.51, 642.06) | 543.82 (470.45, 627.55) | -0.11 (-0.12, -0.1) |
| Uruguay | 24.54 (20.97, 28.14) | 39.15 (34.07, 44.86) | 59.52 | 628.76 (541.01, 717.19) | 595.24 (515.9, 680.56) | -0.23 (-0.25, -0.21) |
| Uzbekistan | 63.64 (54.54, 72.7) | 114.62 (98.14, 130.05) | 80.09 | 608.52 (524.64, 693.37) | 605.59 (519.15, 691.73) | -0.02 (-0.04, 0) |
| Vanuatu | 0.23 (0.19, 0.27) | 0.67 (0.57, 0.78) | 194.09 | 617.37 (527.17, 714.38) | 604.27 (513.73, 697.46) | -0.09 (-0.11, -0.07) |
| Venezuela (Bolivarian Republic of) | 58.36 (50.64, 65.93) | 197.42 (170.95, 222.4) | 238.28 | 732.83 (636.65, 830.94) | 717.86 (620.45, 816.18) | -0.08 (-0.12, -0.04) |
| Viet Nam | 236.1 (202.44, 268.3) | 531.2 (453.59, 601.71) | 124.98 | 689.39 (596.14, 787.87) | 649.42 (556.89, 744.39) | -0.23 (-0.24, -0.22) |
| Yemen | 26.83 (23, 30.75) | 75.92 (66.03, 86.7) | 182.97 | 856.69 (745.98, 974.32) | 777.94 (673.66, 883.49) | -0.32 (-0.35, -0.3) |
| Zambia | 11.35 (9.74, 12.94) | 26.84 (23.11, 30.58) | 136.48 | 589.24 (508.96, 670.23) | 591.53 (514.14, 674.45) | 0.06 (0.04, 0.08) |
| Zimbabwe | 17.12 (14.49, 19.46) | 25.49 (21.69, 29.01) | 48.89 | 611.51 (525.8, 699.36) | 584.43 (506.45, 665.47) | -0.14 (-0.15, -0.13) |

Note: CI, confidence interval; EAPC, estimated annual percentage change; GBD, Global Burden of Disease; UI, uncertainty interval


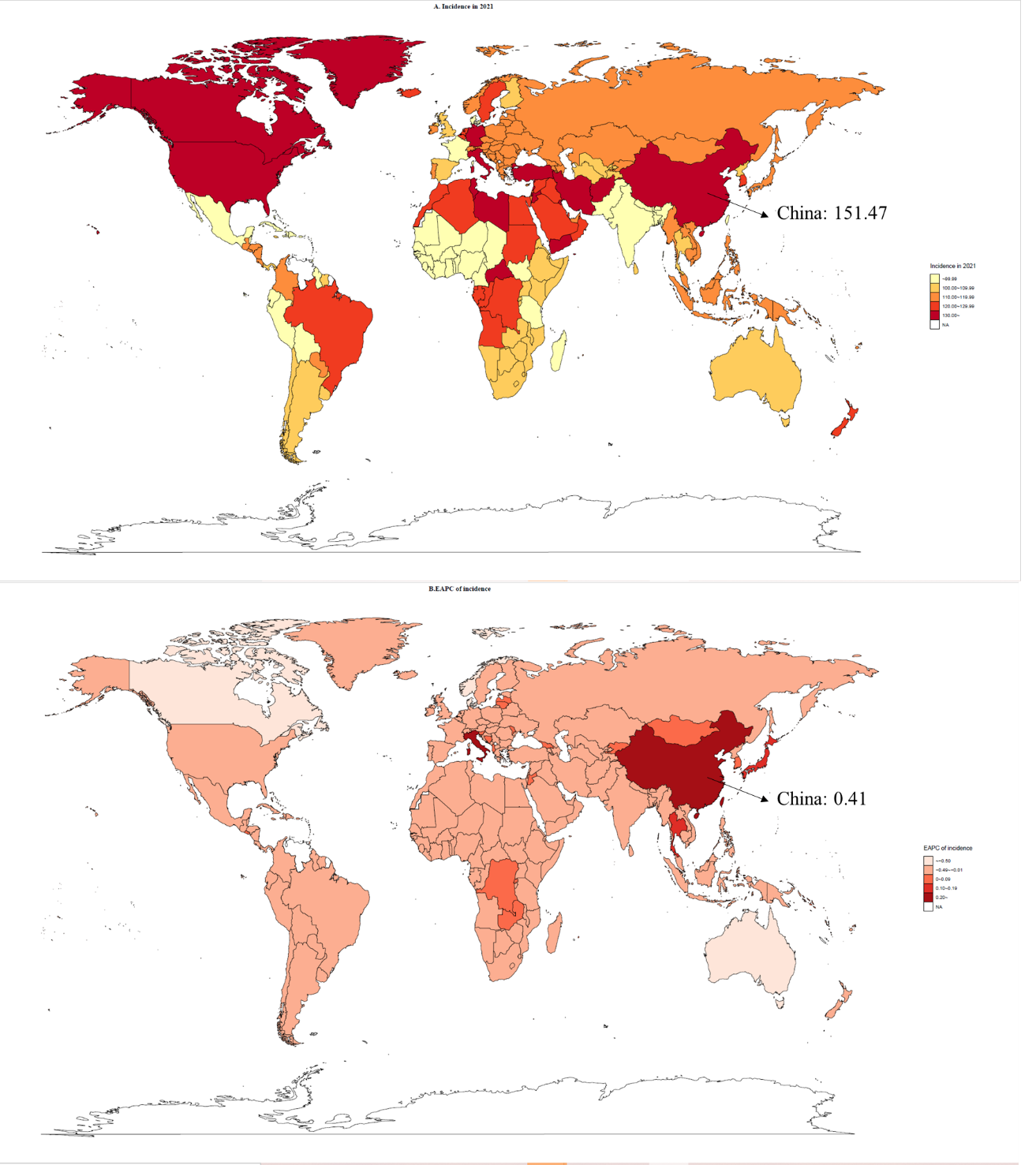


**Figure S2**. **The age-standardized incidence rate for dementia and its temporal trend at national level, 1990−2021**

Note: EAPC, estimated annual percentage change

**Table S4**. **The incident cases and age-standardized incidence rate for dementia and their temporal change at national level, 1990−2021**

|  | **Incident cases (95% UI) per 1000 population** | | | **Age-standardized incidence rate (95% UI), per 100, 000 population** | | |
| --- | --- | --- | --- | --- | --- | --- |
| **Countries and territories** | **1990** | **2021** | **Percentage change (%)** | **1990** | **2021** | **EAPC (95% CI)** |
| Afghanistan | 6.79 (5.88, 7.74) | 8.76 (7.69, 10.03) | 29.03 | 132.83 (116.86, 152.07) | 130.94 (115.12, 149.09) | -0.05 (-0.06, -0.03) |
| Albania | 1.85 (1.61, 2.11) | 4.83 (4.17, 5.53) | 161.16 | 111.95 (97.18, 127.9) | 112.62 (98, 128.7) | 0.04 (0.03, 0.05) |
| Algeria | 12.12 (10.34, 14.09) | 37.17 (32.17, 43.07) | 206.64 | 134.08 (117.34, 152.85) | 128.59 (112.65, 146.91) | -0.14 (-0.15, -0.14) |
| American Samoa | 0.02 (0.01, 0.02) | 0.04 (0.03, 0.05) | 150.27 | 111.24 (95.84, 128.11) | 109.14 (93.39, 125.47) | -0.08 (-0.09, -0.07) |
| Andorra | 0.06 (0.05, 0.07) | 0.19 (0.16, 0.22) | 210.86 | 121.55 (105.04, 139.09) | 114.28 (98.41, 131.55) | -0.19 (-0.2, -0.19) |
| Angola | 2.94 (2.55, 3.33) | 9.17 (7.98, 10.42) | 212.18 | 127.48 (111.77, 145.28) | 124.21 (109.07, 141.23) | -0.08 (-0.09, -0.07) |
| Antigua and Barbuda | 0.06 (0.05, 0.07) | 0.09 (0.08, 0.1) | 53.47 | 97.4 (85.27, 111.61) | 95.08 (82.7, 109.64) | -0.07 (-0.08, -0.06) |
| Argentina | 32.95 (28.37, 37.76) | 62.82 (54.33, 72.19) | 90.66 | 112.19 (97.11, 128.39) | 106.73 (92.2, 122.29) | -0.17 (-0.18, -0.15) |
| Armenia | 2.55 (2.23, 2.92) | 4.93 (4.26, 5.65) | 92.96 | 114.41 (99.75, 130.36) | 114 (99.56, 130.25) | -0.02 (-0.02, -0.01) |
| Australia | 23.45 (20.54, 26.66) | 52.19 (45.95, 58.77) | 122.55 | 122.93 (107.54, 138.36) | 102.66 (90.27, 115.36) | -0.62 (-0.67, -0.58) |
| Austria | 15.98 (13.69, 18.73) | 25.29 (21.75, 29.37) | 58.25 | 123.67 (107.3, 141.71) | 116.09 (99.55, 133.87) | -0.25 (-0.27, -0.22) |
| Azerbaijan | 4.66 (4.08, 5.35) | 8.31 (7.22, 9.44) | 78.28 | 113.74 (99.35, 129.86) | 110.27 (96.65, 126.18) | -0.13 (-0.15, -0.11) |
| Bahamas | 0.13 (0.11, 0.15) | 0.33 (0.28, 0.37) | 155.57 | 97.29 (84.85, 110.7) | 95.28 (82.97, 109.64) | -0.07 (-0.08, -0.07) |
| Bahrain | 0.13 (0.12, 0.15) | 0.69 (0.59, 0.79) | 415.69 | 133.65 (117.28, 152.36) | 130.61 (114.78, 148.7) | -0.05 (-0.06, -0.04) |
| Bangladesh | 30.55 (26.39, 34.91) | 92.25 (79.99, 105.81) | 202.01 | 82.54 (71.13, 94.38) | 79.47 (68.72, 90.87) | -0.12 (-0.13, -0.11) |
| Barbados | 0.32 (0.28, 0.37) | 0.5 (0.43, 0.57) | 55.42 | 98.52 (86.53, 112.22) | 94.55 (82.58, 109.16) | -0.14 (-0.16, -0.12) |
| Belarus | 14.33 (12.33, 16.55) | 19.12 (16.47, 21.92) | 33.4 | 116.59 (101.35, 133.27) | 116.35 (101.03, 133.43) | 0 (-0.01, 0.02) |
| Belgium | 22.51 (19.65, 25.8) | 34.24 (29.72, 39.18) | 52.14 | 136.89 (120.08, 155.5) | 121.59 (105.24, 139.55) | -0.43 (-0.46, -0.4) |
| Belize | 0.09 (0.08, 0.1) | 0.24 (0.21, 0.28) | 166.81 | 101.05 (87.86, 115) | 97.17 (84.64, 110.79) | -0.11 (-0.12, -0.11) |
| Benin | 1.38 (1.2, 1.58) | 2.91 (2.55, 3.32) | 111.18 | 84.72 (73.95, 97.15) | 75.85 (65.91, 87.05) | -0.25 (-0.29, -0.21) |
| Bermuda | 0.06 (0.05, 0.06) | 0.15 (0.13, 0.17) | 166.43 | 99.8 (86.1, 114.94) | 98.24 (84.8, 112.71) | -0.06 (-0.06, -0.05) |
| Bhutan | 0.13 (0.11, 0.15) | 0.42 (0.36, 0.47) | 221.77 | 83.88 (72.92, 96.22) | 77.45 (67.36, 88.68) | -0.25 (-0.27, -0.23) |
| Bolivia (Plurinational State of) | 2.02 (1.74, 2.3) | 6.05 (5.22, 6.92) | 200.16 | 83.32 (72.16, 95.49) | 82.05 (71.22, 94.16) | -0.01 (-0.03, 0.01) |
| Bosnia and Herzegovina | 3.52 (3.01, 4.07) | 7.14 (6.15, 8.27) | 102.76 | 111.71 (96.53, 128.25) | 111.79 (96.79, 127.27) | 0.02 (-0.02, 0.05) |
| Botswana | 0.39 (0.34, 0.45) | 1.06 (0.92, 1.22) | 171.43 | 108.19 (93.86, 123.77) | 103.94 (90.71, 119.61) | -0.1 (-0.12, -0.08) |
| Brazil | 90.51 (79.22, 102.65) | 305.42 (269.29, 345.7) | 237.43 | 129.35 (113.26, 146.89) | 127.08 (112.01, 144.66) | -0.11 (-0.15, -0.08) |
| Brunei Darussalam | 0.08 (0.07, 0.09) | 0.24 (0.2, 0.27) | 203.72 | 101.91 (88.2, 118.19) | 101.56 (88.37, 116.86) | 0.02 (-0.01, 0.06) |
| Bulgaria | 11.72 (9.85, 13.92) | 17.3 (14.85, 20.2) | 47.59 | 114.97 (99.8, 131.97) | 112.43 (97.8, 128.73) | -0.08 (-0.11, -0.06) |
| Burkina Faso | 2.57 (2.21, 2.94) | 5.39 (4.69, 6.14) | 109.87 | 85.31 (73.99, 97.74) | 79.8 (69.33, 91.77) | -0.23 (-0.24, -0.23) |
| Burundi | 1.85 (1.6, 2.12) | 3.24 (2.82, 3.67) | 74.99 | 109.09 (95.51, 124.1) | 100.5 (88.07, 114.87) | -0.24 (-0.26, -0.22) |
| Cabo Verde | 0.2 (0.17, 0.23) | 0.32 (0.28, 0.36) | 61.29 | 82.37 (70.98, 94.3) | 78.13 (67.85, 89.4) | -0.18 (-0.18, -0.17) |
| Cambodia | 3.59 (3.09, 4.1) | 10.16 (8.84, 11.66) | 183.26 | 116.07 (101.32, 132.03) | 112.21 (97.73, 128.37) | -0.16 (-0.18, -0.14) |
| Cameroon | 2.45 (2.12, 2.8) | 6.43 (5.59, 7.32) | 162.05 | 79.7 (69.02, 91.36) | 75.45 (65.39, 86.37) | -0.18 (-0.18, -0.17) |
| Canada | 48.6 (43.43, 54.21) | 105.26 (93.82, 117.16) | 116.58 | 149.59 (134.39, 165.87) | 132.4 (118.02, 147.29) | -0.55 (-0.61, -0.48) |
| Central African Republic | 0.86 (0.74, 0.98) | 1.62 (1.39, 1.84) | 87.54 | 132.85 (116.29, 150.68) | 130.87 (114.21, 148.33) | -0.1 (-0.11, -0.08) |
| Chad | 1.87 (1.62, 2.15) | 3.05 (2.65, 3.47) | 63.02 | 84.07 (72.82, 96.5) | 76.45 (66.54, 87.28) | -0.32 (-0.33, -0.31) |
| Chile | 9.6 (8.39, 10.95) | 28.47 (24.78, 32.54) | 196.5 | 109.85 (96.31, 124.05) | 107.87 (93.91, 123.11) | -0.03 (-0.05, -0.01) |
| China | 703.18 (601.51, 808.63) | 2914.11 (2504.73, 3350.74) | 314.42 | 121.11 (105.5, 137.99) | 151.47 (131.22, 173.34) | 0.41 (0.34, 0.49) |
| Colombia | 16.11 (14.11, 18.3) | 63.67 (55.8, 72.06) | 295.13 | 113.71 (99.52, 130.26) | 112.13 (97.75, 127.51) | -0.03 (-0.05, -0.01) |
| Comoros | 0.14 (0.12, 0.16) | 0.38 (0.33, 0.43) | 176.03 | 105.26 (92.01, 119.38) | 100.96 (88.05, 115.39) | -0.12 (-0.13, -0.11) |
| Congo | 0.86 (0.74, 0.98) | 2.08 (1.89, 2.27) | 142.64 | 124.97 (109.29, 142.35) | 120.8 (109.94, 132.53) | -0.08 (-0.11, -0.06) |
| Cook Islands | 0.01 (0.01, 0.01) | 0.03 (0.02, 0.03) | 161.39 | 111.54 (95.15, 129.33) | 109.53 (93.6, 126.15) | -0.07 (-0.08, -0.06) |
| Costa Rica | 1.81 (1.58, 2.08) | 6.14 (5.38, 6.98) | 238.97 | 113.52 (98.91, 129.64) | 110.62 (95.96, 126.87) | -0.07 (-0.09, -0.06) |
| Cote d'Ivoire | 1.88 (1.63, 2.13) | 5.6 (4.85, 6.4) | 198.43 | 79.55 (69.19, 91.73) | 76.63 (66.06, 88.28) | -0.12 (-0.14, -0.11) |
| Croatia | 6.09 (5.28, 7.06) | 11.3 (9.71, 12.99) | 85.49 | 116.47 (101.76, 133.92) | 112.67 (98.23, 128.39) | -0.08 (-0.1, -0.07) |
| Cuba | 9.05 (7.85, 10.35) | 19.28 (16.87, 21.94) | 112.98 | 92.22 (80.31, 104.84) | 91.41 (79.7, 103.65) | -0.2 (-0.25, -0.16) |
| Cyprus | 0.83 (0.7, 0.98) | 2.46 (2.1, 2.87) | 196.12 | 122.06 (106.32, 139.42) | 117.37 (101.52, 134.87) | -0.09 (-0.11, -0.07) |
| Czechia | 15.05 (12.91, 17.52) | 26.33 (22.78, 30.3) | 74.88 | 111.88 (97.48, 128.14) | 111.23 (96.31, 127.59) | -0.05 (-0.07, -0.04) |
| Democratic People's Republic of Korea | 12.41 (10.61, 14.3) | 31.41 (26.87, 36.67) | 153.09 | 110.89 (95.65, 127.83) | 108.5 (93.89, 125.37) | -0.09 (-0.11, -0.07) |
| Democratic Republic of the Congo | 12.09 (10.45, 13.8) | 29.85 (26.22, 33.72) | 146.83 | 126.38 (110.62, 143.95) | 126.99 (111.77, 144.57) | 0.02 (-0.01, 0.05) |
| Denmark | 9.29 (8.12, 10.64) | 11.47 (9.91, 13.15) | 23.4 | 102.88 (90.99, 117.38) | 84.34 (72.78, 96.21) | -0.73 (-0.76, -0.69) |
| Djibouti | 0.09 (0.07, 0.1) | 0.4 (0.35, 0.45) | 371.36 | 109.58 (95.81, 124.89) | 104.02 (90.57, 118.65) | -0.13 (-0.14, -0.11) |
| Dominica | 0.06 (0.05, 0.07) | 0.07 (0.06, 0.08) | 26.71 | 99.5 (86.83, 113.7) | 96.57 (83.47, 110.4) | -0.09 (-0.1, -0.08) |
| Dominican Republic | 2.99 (2.58, 3.42) | 9.48 (8.27, 10.75) | 217.19 | 99.14 (86.33, 112.62) | 99.53 (86.68, 113.07) | -0.12 (-0.15, -0.08) |
| Ecuador | 3.78 (3.26, 4.33) | 12.52 (10.76, 14.47) | 231.07 | 83.15 (71.74, 95.91) | 81.16 (69.99, 93.4) | -0.03 (-0.05, -0.02) |
| Egypt | 22.68 (19.87, 25.36) | 50.93 (44.46, 57.31) | 124.58 | 130.73 (116.04, 147.5) | 126.46 (111.45, 144.07) | -0.04 (-0.07, -0.02) |
| El Salvador | 3.07 (2.7, 3.48) | 7.62 (6.7, 8.71) | 148.47 | 111.03 (97.01, 126.66) | 112.22 (98.14, 128.45) | 0.06 (0.05, 0.08) |
| Equatorial Guinea | 0.16 (0.14, 0.18) | 0.43 (0.38, 0.49) | 166.34 | 129.4 (113.39, 147.34) | 124.45 (108.79, 141.57) | -0.15 (-0.16, -0.14) |
| Eritrea | 0.62 (0.53, 0.7) | 1.73 (1.5, 1.97) | 181 | 110.64 (96.26, 126.77) | 104.21 (90.44, 119.18) | -0.18 (-0.19, -0.17) |
| Estonia | 2.27 (1.95, 2.64) | 3.66 (3.19, 4.2) | 61.48 | 113.98 (99.49, 130.87) | 112.78 (98.89, 128.28) | -0.01 (-0.05, 0.04) |
| Eswatini | 0.2 (0.18, 0.23) | 0.36 (0.31, 0.41) | 76.89 | 105.08 (91.43, 120.24) | 101.07 (88.65, 115.8) | -0.13 (-0.14, -0.12) |
| Ethiopia | 13.01 (11.19, 14.83) | 34.23 (30.05, 38.96) | 163.15 | 110.74 (96.77, 126.37) | 102.87 (90.07, 116.73) | -0.21 (-0.24, -0.17) |
| Fiji | 0.25 (0.22, 0.29) | 0.58 (0.49, 0.67) | 128.32 | 112.73 (96.97, 130.52) | 110.65 (95.5, 127.91) | -0.08 (-0.09, -0.07) |
| Finland | 9.01 (7.81, 10.31) | 16.66 (14.21, 19.33) | 84.85 | 121.24 (105.54, 137.41) | 108.45 (92.52, 124.81) | -0.4 (-0.41, -0.39) |
| France | 91.99 (82.42, 101.76) | 163.14 (143.06, 184.35) | 77.35 | 99.39 (89.46, 109.62) | 93.63 (82.25, 105.56) | -0.22 (-0.26, -0.19) |
| Gabon | 0.57 (0.49, 0.65) | 0.91 (0.79, 1.04) | 59.99 | 125.84 (109.61, 143.75) | 123.01 (107.72, 140.92) | -0.05 (-0.07, -0.04) |
| Gambia | 0.2 (0.17, 0.23) | 0.57 (0.49, 0.66) | 189.36 | 84.22 (73.25, 96.89) | 77.92 (67.57, 89.88) | -0.26 (-0.27, -0.25) |
| Georgia | 6.4 (5.54, 7.31) | 7.58 (6.6, 8.61) | 18.4 | 113.74 (99.03, 129.23) | 113.98 (99.53, 129.49) | 0.01 (0, 0.02) |
| Germany | 200.15 (178.69, 222.83) | 339.83 (299.33, 382.75) | 69.79 | 145.12 (130.54, 161.52) | 142.13 (124.57, 159.72) | -0.1 (-0.13, -0.07) |
| Ghana | 3.18 (2.73, 3.63) | 8.61 (7.45, 9.81) | 170.98 | 76.92 (66.34, 88.65) | 75.09 (64.72, 86.42) | -0.06 (-0.07, -0.05) |
| Greece | 18.81 (16.04, 21.94) | 37.09 (31.84, 42.95) | 97.21 | 124.2 (107.47, 142.66) | 119.17 (103.16, 136.72) | -0.11 (-0.13, -0.09) |
| Greenland | 0.03 (0.03, 0.03) | 0.07 (0.06, 0.08) | 132.18 | 138.09 (119.55, 159.13) | 135.63 (117.63, 156.11) | -0.07 (-0.1, -0.05) |
| Grenada | 0.08 (0.07, 0.1) | 0.09 (0.08, 0.11) | 10.1 | 99.53 (86.87, 113.92) | 98.02 (85.92, 112.22) | -0.02 (-0.05, 0.01) |
| Guam | 0.05 (0.05, 0.06) | 0.24 (0.21, 0.27) | 351.51 | 109.67 (94.24, 128.33) | 108.97 (93.85, 125.54) | -0.03 (-0.03, -0.02) |
| Guatemala | 2.75 (2.4, 3.13) | 10.94 (9.62, 12.43) | 297.46 | 113.72 (99.12, 129.64) | 112.54 (98.73, 127.7) | -0.02 (-0.03, 0) |
| Guinea | 2.17 (1.89, 2.48) | 3.37 (2.92, 3.85) | 55.09 | 83.16 (72.43, 95.24) | 78.39 (67.86, 90.01) | -0.19 (-0.21, -0.18) |
| Guinea-Bissau | 0.2 (0.17, 0.22) | 0.33 (0.28, 0.38) | 67.28 | 79.09 (68.36, 91.21) | 77.05 (66.66, 89.04) | -0.07 (-0.08, -0.06) |
| Guyana | 0.29 (0.25, 0.33) | 0.48 (0.42, 0.55) | 66.52 | 97.11 (84.1, 111.09) | 96.25 (83.56, 110.32) | -0.04 (-0.05, -0.03) |
| Haiti | 2.21 (1.9, 2.51) | 4.67 (4.07, 5.35) | 111.38 | 102.3 (89.46, 116.07) | 94.82 (82.42, 108.66) | -0.26 (-0.28, -0.25) |
| Honduras | 1.84 (1.61, 2.11) | 5.68 (4.92, 6.49) | 207.65 | 115.03 (100.77, 131.35) | 113.19 (98.75, 129.15) | -0.05 (-0.06, -0.05) |
| Hungary | 15.68 (13.5, 18.32) | 24.33 (21.06, 27.87) | 55.12 | 112.52 (98.61, 128.63) | 111.47 (96.69, 127.63) | -0.02 (-0.05, 0.02) |
| Iceland | 0.42 (0.36, 0.47) | 0.8 (0.69, 0.9) | 92.01 | 135.01 (117.98, 152.05) | 122.36 (106.34, 137.53) | -0.36 (-0.38, -0.35) |
| India | 241.55 (209.57, 274.95) | 749.49 (648.65, 856.82) | 210.28 | 79.45 (68.69, 90.82) | 78.92 (68.29, 90.58) | -0.16 (-0.21, -0.11) |
| Indonesia | 78.84 (68.68, 89.4) | 189.72 (164.14, 216.6) | 140.64 | 115.97 (101.07, 132) | 113.31 (98.38, 129.52) | -0.07 (-0.09, -0.05) |
| Iran (Islamic Republic of) | 23.33 (20.15, 26.48) | 87.94 (77.51, 99.88) | 276.85 | 137.67 (120.79, 156.35) | 133.73 (117.53, 151.85) | -0.08 (-0.09, -0.07) |
| Iraq | 9.44 (8.25, 10.71) | 22.2 (19.38, 25.18) | 135.25 | 135.28 (117.85, 154.35) | 129.62 (113.05, 148.98) | -0.17 (-0.19, -0.16) |
| Ireland | 4.99 (4.27, 5.79) | 9.58 (8.21, 11.08) | 91.98 | 122.27 (105.91, 140.72) | 111.99 (95.75, 128.99) | -0.3 (-0.32, -0.27) |
| Israel | 5.74 (4.86, 6.71) | 15.41 (13.25, 17.72) | 168.36 | 120.3 (103.78, 138.38) | 113.13 (97.01, 130.26) | -0.21 (-0.22, -0.2) |
| Italy | 106.54 (89.87, 123.11) | 248.81 (215.07, 285.48) | 133.53 | 116.29 (98.96, 133.09) | 134.76 (116.74, 153.65) | 0.28 (0.09, 0.46) |
| Jamaica | 2.08 (1.82, 2.38) | 3.49 (3.05, 3.97) | 67.91 | 110.63 (96.95, 126.27) | 104.77 (91.01, 120.07) | -0.2 (-0.21, -0.18) |
| Japan | 184.31 (160.12, 210.89) | 576.27 (503.46, 662.79) | 212.67 | 115.81 (101.01, 131.94) | 117.23 (102.05, 133.68) | 0.2 (0.15, 0.25) |
| Jordan | 1.22 (1.06, 1.39) | 7.24 (6.31, 8.27) | 494.01 | 133.38 (116.04, 152.85) | 134.51 (116.99, 153.81) | 0.07 (0.03, 0.1) |
| Kazakhstan | 11.93 (10.45, 13.73) | 15.44 (13.39, 17.64) | 29.5 | 113.75 (99.27, 130.56) | 111.1 (96.76, 126.81) | -0.08 (-0.1, -0.05) |
| Kenya | 6.57 (5.71, 7.46) | 16.49 (14.43, 18.73) | 150.99 | 106.99 (93.29, 121.75) | 105.14 (91.55, 119.62) | -0.06 (-0.08, -0.04) |
| Kiribati | 0.03 (0.02, 0.03) | 0.05 (0.05, 0.06) | 92.29 | 121.16 (104.74, 139.34) | 121.84 (106.12, 138.96) | 0.01 (-0.02, 0.04) |
| Kuwait | 0.54 (0.48, 0.61) | 2.81 (2.48, 3.16) | 416.92 | 137.77 (120.63, 157) | 131.46 (115.44, 149.34) | -0.15 (-0.17, -0.13) |
| Kyrgyzstan | 2.91 (2.55, 3.33) | 4.13 (3.62, 4.68) | 41.89 | 112.94 (98.41, 128.4) | 113.88 (99.39, 130.07) | 0.05 (0.04, 0.06) |
| Lao People's Democratic Republic | 1.57 (1.34, 1.8) | 3.76 (3.28, 4.29) | 139.99 | 114.26 (100.61, 130.8) | 111.82 (97.43, 127.89) | -0.06 (-0.07, -0.04) |
| Latvia | 4.05 (3.49, 4.71) | 5.47 (4.73, 6.28) | 34.83 | 114.61 (99.77, 131.15) | 114.97 (99.97, 131) | 0.04 (0.02, 0.06) |
| Lebanon | 2.4 (2.11, 2.74) | 9.17 (8, 10.45) | 281.86 | 139.66 (122.17, 159.68) | 140.48 (122.53, 159.62) | 0 (-0.01, 0.01) |
| Lesotho | 0.74 (0.64, 0.84) | 0.79 (0.68, 0.91) | 7.09 | 110.5 (95.98, 126.11) | 108.92 (94.83, 124.74) | -0.02 (-0.03, -0.01) |
| Liberia | 0.66 (0.57, 0.76) | 1.1 (0.96, 1.25) | 65.79 | 77.77 (67.19, 89.07) | 75.16 (64.95, 86.14) | -0.11 (-0.12, -0.1) |
| Libya | 2.15 (1.9, 2.43) | 5.29 (4.62, 6) | 145.41 | 137.01 (120.05, 155.79) | 130.47 (114.04, 148.13) | -0.16 (-0.18, -0.14) |
| Lithuania | 5.08 (4.37, 5.89) | 7.77 (6.69, 9.02) | 52.82 | 113.06 (98.58, 129.94) | 112.73 (98.22, 129.2) | 0.02 (-0.01, 0.05) |
| Luxembourg | 0.56 (0.47, 0.65) | 1.03 (0.88, 1.19) | 85.16 | 100.78 (86.37, 116.34) | 87.36 (74.31, 101.13) | -0.51 (-0.57, -0.45) |
| Madagascar | 3.78 (3.27, 4.3) | 6.74 (5.81, 7.65) | 78.35 | 105.1 (91.62, 120.32) | 99.76 (86.8, 114.13) | -0.15 (-0.17, -0.14) |
| Malawi | 2.68 (2.32, 3.06) | 5.28 (4.6, 6.03) | 96.8 | 105.4 (91.35, 120.59) | 104.71 (90.82, 119.89) | -0.01 (-0.02, 0.01) |
| Malaysia | 9.07 (7.98, 10.27) | 26.05 (22.44, 29.86) | 187.13 | 116.13 (101.28, 132.46) | 111.29 (95.99, 127.46) | -0.12 (-0.13, -0.1) |
| Maldives | 0.06 (0.05, 0.07) | 0.31 (0.27, 0.36) | 395.41 | 109.84 (95.49, 126.07) | 113.22 (98.24, 129.67) | 0.1 (0.07, 0.13) |
| Mali | 2.12 (1.83, 2.42) | 4.71 (4.07, 5.36) | 121.77 | 81.91 (71.19, 93.65) | 78.55 (68.4, 89.84) | -0.13 (-0.14, -0.12) |
| Malta | 0.49 (0.42, 0.57) | 1.3 (1.11, 1.49) | 164.68 | 122.8 (106.32, 141.45) | 113.91 (98.03, 130.83) | -0.22 (-0.25, -0.2) |
| Marshall Islands | 0.01 (0.01, 0.01) | 0.02 (0.02, 0.02) | 75.85 | 107.43 (92.62, 124.49) | 103.94 (89.43, 120.5) | -0.11 (-0.11, -0.1) |
| Mauritania | 0.63 (0.54, 0.72) | 1.32 (1.14, 1.5) | 110.04 | 83.53 (71.72, 96.45) | 77.53 (67.33, 88.83) | -0.24 (-0.25, -0.23) |
| Mauritius | 0.62 (0.53, 0.71) | 1.88 (1.63, 2.14) | 205.78 | 112.36 (97.71, 130.05) | 111.73 (96.55, 127.55) | 0.01 (-0.01, 0.04) |
| Mexico | 36.27 (31.43, 41.67) | 110.47 (96.17, 125.49) | 204.58 | 106.03 (91.84, 120.63) | 97.19 (84.27, 111.26) | -0.19 (-0.23, -0.15) |
| Micronesia (Federated States of) | 0.04 (0.04, 0.05) | 0.06 (0.05, 0.06) | 31 | 119.8 (102.66, 137.56) | 120.71 (104.84, 139.46) | 0.06 (0.05, 0.07) |
| Monaco | 0.1 (0.09, 0.12) | 0.14 (0.12, 0.16) | 30.02 | 123.3 (106.75, 141.67) | 113.72 (97.81, 131.33) | -0.28 (-0.28, -0.27) |
| Mongolia | 0.95 (0.82, 1.1) | 1.82 (1.6, 2.05) | 91 | 114.4 (99.85, 130.62) | 115.83 (101.21, 132.52) | 0.05 (0.04, 0.06) |
| Montenegro | 0.65 (0.57, 0.75) | 1.01 (0.86, 1.16) | 53.86 | 114.61 (100.1, 131.64) | 111.66 (97.13, 128.15) | -0.12 (-0.16, -0.08) |
| Morocco | 16.23 (14.14, 18.42) | 36.51 (31.64, 41.83) | 124.92 | 136.84 (120, 155.37) | 127.85 (111.31, 146.15) | -0.23 (-0.23, -0.22) |
| Mozambique | 4.33 (3.74, 4.93) | 7.52 (6.51, 8.55) | 73.44 | 107.72 (94.15, 122.63) | 104.33 (90.49, 119.04) | -0.08 (-0.1, -0.07) |
| Myanmar | 19.76 (17.26, 22.52) | 44.03 (38.39, 49.95) | 122.79 | 122.22 (107.45, 139.08) | 113.09 (99.06, 128.82) | -0.27 (-0.28, -0.25) |
| Namibia | 0.45 (0.39, 0.52) | 1.01 (0.88, 1.15) | 122.96 | 107.16 (93.53, 121.95) | 103.46 (89.84, 117.7) | -0.11 (-0.12, -0.09) |
| Nauru | 0 (0, 0) | 0 (0, 0) | 41.91 | 110.81 (95.43, 128.03) | 113.97 (98.71, 131.23) | 0.06 (0.02, 0.1) |
| Nepal | 5.69 (4.97, 6.48) | 14.86 (12.92, 16.96) | 161.25 | 91.22 (79.31, 104.24) | 81.31 (70.51, 93.7) | -0.45 (-0.48, -0.42) |
| Netherlands | 26.95 (23.61, 29.77) | 48.27 (42.17, 54.73) | 79.14 | 127.6 (112.23, 140.58) | 121.79 (106.84, 137.86) | -0.14 (-0.16, -0.11) |
| New Zealand | 4.96 (4.29, 5.7) | 11.1 (9.64, 12.75) | 123.87 | 128.02 (110.92, 146.45) | 120.84 (104.92, 138.57) | -0.22 (-0.25, -0.19) |
| Nicaragua | 1.44 (1.27, 1.64) | 4.88 (4.28, 5.56) | 238.32 | 115.45 (101.47, 131.74) | 114.62 (100.58, 131.05) | 0 (-0.01, 0.01) |
| Niger | 1.43 (1.24, 1.63) | 4.3 (3.71, 4.92) | 199.86 | 83.26 (72.29, 95.94) | 78.36 (68.05, 90.06) | -0.2 (-0.21, -0.19) |
| Nigeria | 24.79 (21.49, 28.16) | 45.31 (39.64, 51.19) | 82.75 | 75.45 (65.4, 86.12) | 68.83 (59.58, 78.6) | -0.31 (-0.35, -0.27) |
| Niue | 0 (0, 0) | 0 (0, 0) | -20.69 | 110.25 (95.02, 127.06) | 107.28 (91.33, 123.83) | -0.11 (-0.12, -0.1) |
| North Macedonia | 1.75 (1.52, 2.01) | 3.03 (2.59, 3.53) | 72.97 | 111.85 (97.22, 128.3) | 111.12 (96.41, 127.65) | -0.01 (-0.02, 0) |
| Northern Mariana Islands | 0.01 (0.01, 0.01) | 0.04 (0.03, 0.04) | 278.05 | 109.52 (93.43, 127.91) | 107.94 (92.46, 125.17) | -0.04 (-0.05, -0.03) |
| Norway | 10.64 (9.28, 12.22) | 13.15 (11.35, 15.11) | 23.58 | 136.54 (119.38, 155.02) | 114.3 (98.69, 131.41) | -0.64 (-0.69, -0.6) |
| Oman | 0.64 (0.55, 0.72) | 1.62 (1.4, 1.86) | 154.43 | 132.92 (116.54, 151.46) | 124.36 (108.14, 143.21) | -0.2 (-0.24, -0.15) |
| Pakistan | 37.27 (32.34, 42.65) | 67.82 (58.77, 77.29) | 81.97 | 84.44 (73.08, 97.03) | 78.8 (67.99, 90.38) | -0.25 (-0.26, -0.24) |
| Palau | 0.01 (0.01, 0.01) | 0.02 (0.01, 0.02) | 107.95 | 107.16 (91.89, 124.54) | 103.98 (88.42, 120.53) | -0.09 (-0.09, -0.08) |
| Palestine | 0.96 (0.84, 1.09) | 2.4 (2.1, 2.73) | 150.03 | 136.47 (119.22, 154.4) | 131.55 (115.83, 150.08) | -0.13 (-0.16, -0.1) |
| Panama | 1.47 (1.29, 1.68) | 4.94 (4.34, 5.64) | 235.78 | 110.21 (95.8, 125.86) | 108.64 (94.58, 124.54) | -0.05 (-0.06, -0.05) |
| Papua New Guinea | 1.23 (1.05, 1.41) | 3.5 (3.03, 3.98) | 184.88 | 120.02 (105.08, 136.87) | 112.89 (97.86, 129.7) | -0.25 (-0.28, -0.22) |
| Paraguay | 2.43 (2.12, 2.76) | 6.14 (5.39, 6.97) | 152.42 | 123.19 (107.15, 139.9) | 115.77 (101.31, 132.33) | -0.22 (-0.23, -0.21) |
| Peru | 8.3 (7.19, 9.39) | 25.92 (22.44, 29.53) | 212.36 | 78.97 (68.46, 90.1) | 78.28 (67.57, 89.77) | -0.09 (-0.11, -0.06) |
| Philippines | 25.71 (22.3, 29.46) | 72.09 (63.05, 81.98) | 180.35 | 119.03 (104.7, 135.62) | 113.91 (99.74, 130) | -0.18 (-0.19, -0.16) |
| Poland | 48.71 (41.95, 56.29) | 88.41 (76.69, 101.19) | 81.49 | 120.58 (105.09, 137.37) | 114.27 (99.44, 130.4) | -0.18 (-0.19, -0.17) |
| Portugal | 15.78 (13.6, 18.44) | 35.67 (30.67, 41.51) | 126.09 | 118.74 (102.91, 136.33) | 116.97 (100.45, 134.76) | -0.02 (-0.04, 0) |
| Puerto Rico | 3.51 (3.01, 4.02) | 8.78 (7.67, 10.04) | 150.05 | 99.86 (86.41, 113.62) | 97.8 (84.88, 112.14) | -0.08 (-0.09, -0.07) |
| Qatar | 0.08 (0.07, 0.08) | 0.64 (0.55, 0.72) | 751.51 | 129.42 (112.13, 147.88) | 126.56 (110.1, 145.4) | -0.01 (-0.05, 0.02) |
| Republic of Korea | 26.57 (23.16, 30.28) | 117.49 (102.91, 133.13) | 342.26 | 127.7 (112.52, 145.12) | 124.63 (109.44, 140.89) | 0.01 (-0.04, 0.06) |
| Republic of Moldova | 3.86 (3.32, 4.45) | 6.8 (5.91, 7.74) | 76.1 | 110.64 (96.04, 126.38) | 111.97 (97.12, 128.21) | 0.03 (0.01, 0.05) |
| Romania | 26.48 (22.58, 30.91) | 46.18 (39.79, 53.46) | 74.38 | 111.56 (96.96, 128.05) | 111.32 (96.82, 127.73) | -0.03 (-0.05, -0.02) |
| Russian Federation | 183.44 (158.33, 212.15) | 279.81 (243.53, 319.87) | 52.53 | 117.07 (102.29, 133.67) | 115.89 (101.23, 131.98) | -0.07 (-0.12, -0.03) |
| Rwanda | 1.93 (1.66, 2.19) | 4.61 (4.02, 5.19) | 139.17 | 107.03 (93.96, 122.04) | 106.57 (93.24, 120.76) | 0.04 (0.02, 0.06) |
| Saint Kitts and Nevis | 0.03 (0.03, 0.04) | 0.05 (0.04, 0.06) | 39.21 | 94.29 (82.57, 107.74) | 93.38 (80.85, 107.47) | -0.04 (-0.05, -0.03) |
| Saint Lucia | 0.08 (0.07, 0.09) | 0.22 (0.19, 0.25) | 187.19 | 98.8 (85.78, 113.56) | 95.98 (82.99, 109.74) | -0.12 (-0.14, -0.1) |
| Saint Vincent and the Grenadines | 0.07 (0.06, 0.08) | 0.13 (0.11, 0.15) | 89.83 | 101.6 (88.04, 115.94) | 96.56 (84.19, 110.27) | -0.16 (-0.17, -0.16) |
| Samoa | 0.07 (0.06, 0.08) | 0.12 (0.11, 0.14) | 77.14 | 111.48 (96.61, 128.63) | 108.37 (93.13, 125.19) | -0.11 (-0.12, -0.1) |
| San Marino | 0.05 (0.04, 0.05) | 0.11 (0.09, 0.12) | 124.25 | 124.29 (108.62, 141.54) | 110.9 (96.02, 127.71) | -0.38 (-0.4, -0.36) |
| Sao Tome and Principe | 0.04 (0.04, 0.05) | 0.06 (0.05, 0.07) | 42.73 | 78.52 (67.94, 90.02) | 73.4 (63.08, 84.79) | -0.21 (-0.23, -0.19) |
| Saudi Arabia | 5.4 (4.68, 6.17) | 13.06 (11.31, 14.78) | 141.8 | 127.34 (111.13, 146.35) | 120.44 (104.42, 138.45) | -0.19 (-0.2, -0.17) |
| Senegal | 1.96 (1.69, 2.23) | 4.51 (3.92, 5.17) | 130.79 | 82.21 (71.27, 94.43) | 76.64 (66.53, 88.26) | -0.26 (-0.26, -0.25) |
| Serbia | 9.63 (8.17, 11.1) | 19.54 (16.95, 22.54) | 102.83 | 111.13 (96.63, 127.15) | 111.65 (96.91, 128.06) | 0 (-0.01, 0.01) |
| Seychelles | 0.06 (0.05, 0.07) | 0.11 (0.09, 0.12) | 72.06 | 113.14 (98.39, 129.4) | 109.25 (94.47, 126.6) | -0.13 (-0.14, -0.13) |
| Sierra Leone | 1.36 (1.17, 1.56) | 2.2 (1.91, 2.53) | 61.82 | 83.78 (72.77, 95.51) | 79.15 (68.54, 91.02) | -0.18 (-0.19, -0.17) |
| Singapore | 1.57 (1.36, 1.76) | 7.76 (6.99, 8.58) | 394.02 | 91.55 (80.57, 102.5) | 94.88 (85.54, 104.85) | 0.15 (0.12, 0.17) |
| Slovakia | 6.38 (5.51, 7.37) | 10.65 (9.28, 12.23) | 66.9 | 112.67 (98.27, 128.86) | 110.87 (97.08, 127) | -0.05 (-0.06, -0.04) |
| Slovenia | 2.67 (2.31, 3.08) | 5.73 (4.98, 6.58) | 115.08 | 110.1 (96.03, 125.51) | 110.51 (96.63, 126.67) | 0.05 (0.02, 0.08) |
| Solomon Islands | 0.09 (0.08, 0.1) | 0.26 (0.22, 0.3) | 183.89 | 113.58 (98.83, 130.2) | 113.74 (98.07, 131.08) | 0.05 (0.02, 0.07) |
| Somalia | 1.48 (1.28, 1.67) | 3.59 (3.09, 4.11) | 143.27 | 107.79 (94.2, 122.85) | 106.72 (93.34, 121.51) | -0.02 (-0.02, -0.01) |
| South Africa | 19.44 (16.98, 21.98) | 39.71 (34.53, 45.26) | 104.27 | 114.18 (99.52, 130.09) | 107.78 (93.63, 122.59) | -0.16 (-0.17, -0.14) |
| South Sudan | 1.98 (1.72, 2.27) | 2.57 (2.23, 2.9) | 29.56 | 105.62 (92.12, 120.57) | 99.16 (85.85, 113.38) | -0.19 (-0.21, -0.16) |
| Spain | 65.15 (57.01, 72.71) | 123.77 (107.15, 142.15) | 89.96 | 117.06 (103.03, 129.59) | 102.5 (88.99, 116.45) | -0.37 (-0.44, -0.3) |
| Sri Lanka | 9.12 (7.92, 10.42) | 25.79 (22.3, 29.88) | 182.72 | 111.29 (96.77, 127.29) | 107.46 (93.3, 123.54) | -0.13 (-0.14, -0.12) |
| Sudan | 9.49 (8.22, 10.86) | 18.54 (16.24, 21.02) | 95.36 | 134.77 (118.25, 154.07) | 125.81 (110.08, 143.27) | -0.23 (-0.24, -0.22) |
| Suriname | 0.23 (0.2, 0.26) | 0.58 (0.51, 0.66) | 149.3 | 105.02 (91.54, 120.14) | 100.19 (87.41, 115.47) | -0.16 (-0.18, -0.15) |
| Sweden | 24.22 (21.34, 27.35) | 33.51 (29.06, 38.43) | 38.36 | 137.72 (121.98, 154.24) | 126.32 (109.55, 144.06) | -0.24 (-0.27, -0.2) |
| Switzerland | 14.54 (12.61, 16.72) | 24.78 (21.5, 28.44) | 70.41 | 124.61 (108.66, 142.14) | 113.27 (97.88, 129.61) | -0.34 (-0.36, -0.32) |
| Syrian Arab Republic | 5.63 (4.95, 6.36) | 13.05 (11.34, 14.94) | 132.01 | 139.41 (122.36, 159.02) | 129.77 (113.5, 147.95) | -0.24 (-0.25, -0.23) |
| Taiwan (Province of China) | 10.34 (8.83, 11.88) | 43.2 (36.98, 48.56) | 317.97 | 91.21 (78.72, 104.55) | 98.1 (83.69, 110.2) | 0.38 (0.29, 0.47) |
| Tajikistan | 2.62 (2.3, 2.98) | 4.16 (3.63, 4.72) | 58.74 | 111.93 (98.19, 127.63) | 104.98 (92.03, 120.4) | -0.23 (-0.23, -0.22) |
| Thailand | 27.75 (24.16, 31.44) | 112.6 (98.74, 127.88) | 305.81 | 102.51 (89.15, 116.4) | 103.27 (90.64, 117.58) | 0.12 (0.06, 0.17) |
| Timor-Leste | 0.21 (0.19, 0.24) | 0.77 (0.67, 0.89) | 259.7 | 120.37 (105.37, 137.35) | 113.2 (98.46, 129.74) | -0.18 (-0.2, -0.16) |
| Togo | 0.67 (0.59, 0.77) | 1.9 (1.63, 2.16) | 182.31 | 81.07 (70.07, 92.89) | 78.26 (67.73, 90) | -0.09 (-0.1, -0.09) |
| Tokelau | 0 (0, 0) | 0 (0, 0) | 24.04 | 111.36 (95.94, 128.64) | 109.37 (94.11, 126.6) | -0.06 (-0.07, -0.05) |
| Tonga | 0.05 (0.04, 0.06) | 0.08 (0.07, 0.1) | 71.07 | 115.12 (99.63, 133.62) | 111.8 (97.12, 128.75) | -0.11 (-0.12, -0.09) |
| Trinidad and Tobago | 0.73 (0.63, 0.85) | 1.82 (1.59, 2.07) | 147.9 | 100.82 (87.88, 115.33) | 98.6 (85.73, 112.51) | -0.06 (-0.07, -0.05) |
| Tunisia | 5.48 (4.75, 6.26) | 15.84 (13.95, 18.01) | 189.16 | 142.94 (125.86, 162.19) | 134.32 (117.99, 153.04) | -0.19 (-0.2, -0.18) |
| Turkey | 40.37 (35.49, 45.67) | 117.99 (102.84, 133.4) | 192.27 | 147.22 (129.54, 166.99) | 139.1 (120.57, 158.16) | -0.22 (-0.24, -0.2) |
| Turkmenistan | 1.64 (1.44, 1.86) | 3.3 (2.87, 3.74) | 100.6 | 112.55 (98.74, 128.5) | 107.05 (93.39, 122.99) | -0.18 (-0.21, -0.16) |
| Tuvalu | 0 (0, 0.01) | 0.01 (0.01, 0.01) | 83.55 | 116.09 (100.33, 134.46) | 113.41 (97.97, 130.03) | -0.07 (-0.08, -0.06) |
| Uganda | 4.8 (4.14, 5.48) | 10.73 (9.35, 12.17) | 123.42 | 104.09 (91.02, 118.56) | 102.68 (89.05, 117.37) | -0.05 (-0.07, -0.02) |
| Ukraine | 78 (66.9, 90.22) | 93.47 (80.74, 107.94) | 19.84 | 119.03 (103.58, 135.93) | 115.57 (100.64, 132.68) | -0.11 (-0.15, -0.07) |
| United Arab Emirates | 0.31 (0.27, 0.36) | 2.32 (1.96, 2.69) | 642.45 | 123.53 (107.13, 142.63) | 113.49 (98.05, 131.08) | -0.26 (-0.29, -0.23) |
| United Kingdom | 112.87 (97.2, 130.28) | 164.95 (142.72, 190.06) | 46.14 | 114.58 (99.74, 130.77) | 107.93 (93.37, 123.89) | -0.17 (-0.2, -0.13) |
| United Republic of Tanzania | 8.09 (7.04, 9.22) | 18.87 (16.81, 21.1) | 133.26 | 107.04 (92.95, 122.58) | 98.48 (87.78, 110.14) | -0.25 (-0.26, -0.23) |
| United States of America | 471.56 (410.8, 540.65) | 822.91 (718.8, 935.11) | 74.51 | 138.61 (120.85, 157.59) | 131.29 (113.93, 149.6) | -0.19 (-0.2, -0.17) |
| United States Virgin Islands | 0.06 (0.05, 0.07) | 0.18 (0.16, 0.21) | 186.11 | 97.53 (84.95, 111.49) | 94.57 (82.02, 109.17) | -0.11 (-0.12, -0.11) |
| Uruguay | 4.42 (3.83, 5.07) | 7.02 (6.12, 8.05) | 58.69 | 112.68 (98.67, 128.38) | 107.09 (93.07, 121.78) | -0.21 (-0.23, -0.19) |
| Uzbekistan | 11.12 (9.61, 12.82) | 20.09 (17.47, 22.88) | 80.72 | 106.71 (92.5, 122.44) | 106.19 (92.22, 122.08) | -0.02 (-0.04, -0.01) |
| Vanuatu | 0.04 (0.03, 0.05) | 0.12 (0.1, 0.14) | 194.34 | 107.32 (92.84, 124.06) | 105.3 (90.64, 121.61) | -0.08 (-0.1, -0.06) |
| Venezuela (Bolivarian Republic of) | 10.21 (8.94, 11.61) | 34.33 (30.25, 38.77) | 236.29 | 127.09 (110.71, 143.85) | 124.37 (108.4, 141.62) | -0.08 (-0.12, -0.05) |
| Viet Nam | 39.85 (34.72, 45.6) | 90.17 (78.82, 102.84) | 126.27 | 116.54 (102.61, 133.79) | 110.09 (96.21, 126.57) | -0.22 (-0.23, -0.21) |
| Yemen | 4.71 (4.1, 5.38) | 13.41 (11.72, 15.19) | 184.58 | 144.23 (127.32, 163.56) | 133.11 (117.14, 151.66) | -0.27 (-0.29, -0.24) |
| Zambia | 1.99 (1.72, 2.26) | 4.72 (4.11, 5.33) | 137.78 | 102.35 (89.13, 116.62) | 102.65 (89.99, 117.22) | 0.06 (0.04, 0.08) |
| Zimbabwe | 3 (2.58, 3.42) | 4.51 (3.92, 5.13) | 50.29 | 106.36 (93.06, 121.67) | 102.44 (89.41, 116.93) | -0.11 (-0.12, -0.1) |

Note: CI, confidence interval; EAPC, estimated annual percentage change; GBD, Global Burden of Disease; UI, uncertainty interval


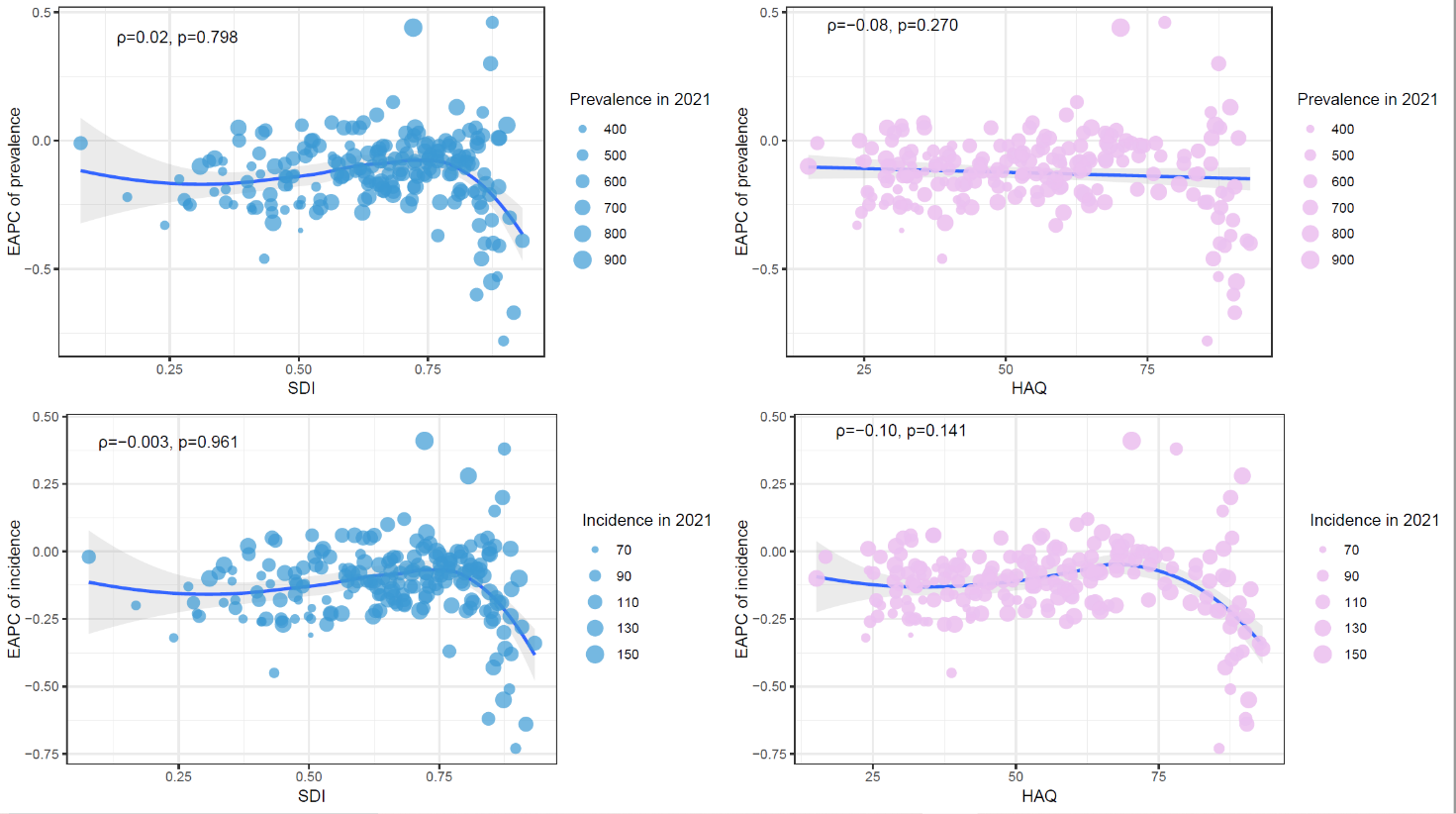


**Figure S3**. **The correlations between EAPC of age-standardized prevalence rate, age-standardized incidence rate, SDI, and HAQ index.**

Notes: EAPC, estimated annual percentage change; HAQ, healthcare access and 1uality index; SDI, socio-demographic Index


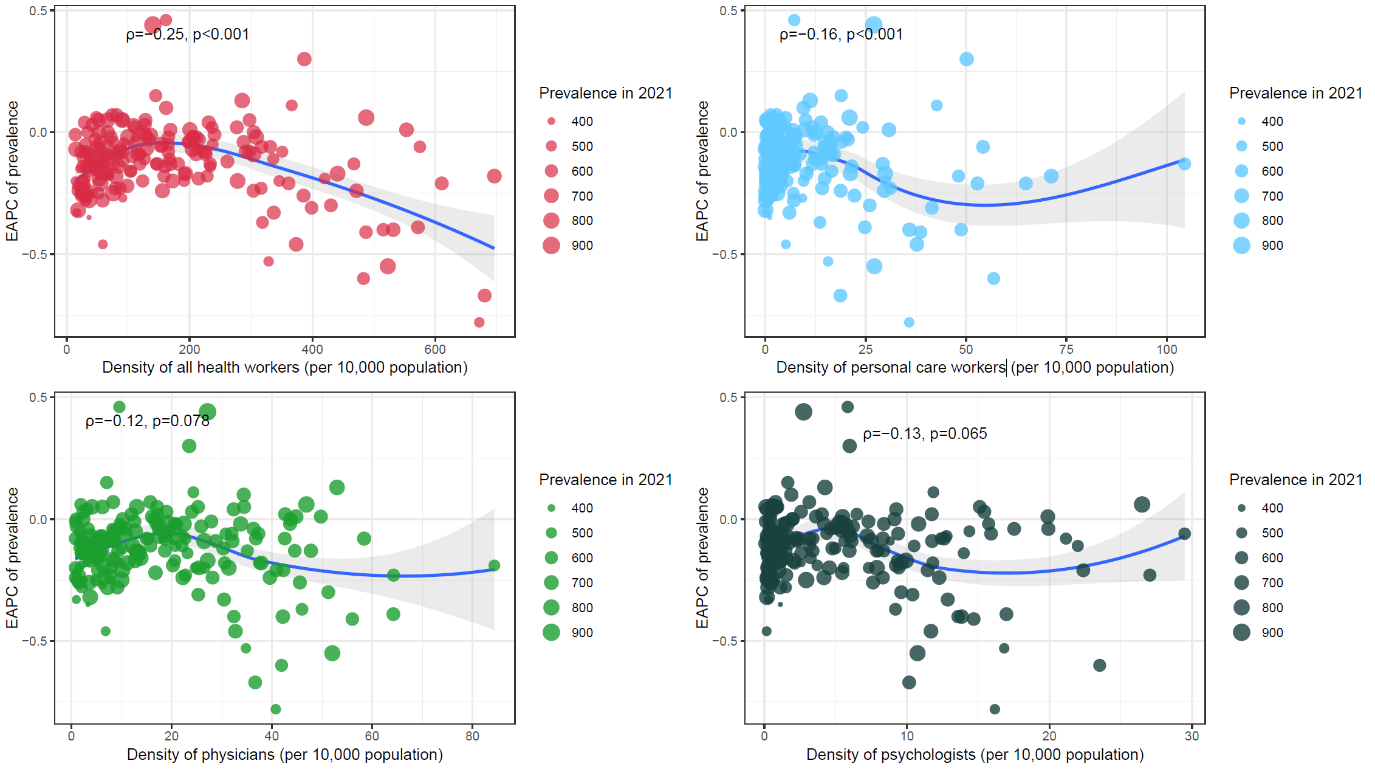


**Figure S4**. **The correlations between EAPC of age-standardized prevalence rate and HRH.**

Notes: EAPC, estimated annual percentage change; HRH, human resources for health

**Table S5**. **The DALYs and age-standardized DALYs rate for dementia and their temporal change at global and regional level, 1990−2021**

|  | **DALYs (95% UI) per 1000 population** | | | **Age-standardized DALYs rate (95% UI), per 100,000 population** | | |
| --- | --- | --- | --- | --- | --- | --- |
| **Regions** | **1990** | **2021** | **Percentage change (%)** | **1990** | **2021** | **EAPC (95% CI)** |
| **Global** | 13572.31 (6439.34, 29586.87) | 36332.69 (17237.62, 76873.28) | 167.7 | 445.75 (206.08, 958.03) | 450.98 (212.69, 950.16) | -0.02 (-0.03, -0.01) |
| **Gender** |  |  |  |  |  |  |
| Male | 4465.56 (2092.52, 9971.77) | 12524.13 (5871.76, 27158.68) | 180.46 | 362.99 (164.92, 799.27) | 372.53 (170.89, 805.03) | 0.05 (0.03, 0.06) |
| Female | 9106.75 (4330.35, 19615.10) | 23808.56 (11368.14, 49746.52) | 161.44 | 495.05 (231.43, 1054.27) | 504.87 (241.04, 1055.02) | 0.001 (-0.01, 0.02) |
| **Socio-demographic index** |  |  |  |  |  |  |
| High-middle SDI | 3550.38 (1664.39, 7789.66) | 9243.77 (4399.64, 19638.39) | 160.36 | 460.79 (211.15, 995.92) | 481.7 (228.79, 1023.96) | 0.07 (0.04, 0.09) |
| High SDI | 5178.32 (2446.91, 11074.2) | 11731.99 (5566.77, 24033.2) | 126.56 | 475.13 (223.26, 1007.56) | 460.75 (220.37, 948.43) | -0.11 (-0.12, -0.1) |
| Low-middle SDI | 1357.81 (641.76, 3022.1) | 3925.27 (1774.1, 8561.48) | 189.09 | 334.98 (157.27, 731.97) | 360.4 (164.09, 783.93) | 0.23 (0.22, 0.24) |
| Low SDI | 495.71 (228.39, 1102.22) | 1260.61 (565.04, 2856.16) | 154.31 | 358.32 (163.29, 788.84) | 383.04 (167.71, 863.74) | 0.24 (0.18, 0.29) |
| Middle SDI | 2975.64 (1398.26, 6593.08) | 10141.16 (4901.24, 21854.76) | 240.81 | 434.43 (200.09, 947.97) | 455.35 (215.96, 982.76) | 0.03 (0, 0.05) |
| **GBD region** |  |  |  |  |  |  |
| High-income North America | 1931.57 (915.98, 4111.23) | 3664.55 (1728.3, 7650.51) | 89.72 | 522.13 (247.4, 1104.02) | 499.2 (236.41, 1041.63) | -0.18 (-0.19, -0.16) |
| Caribbean | 71.56 (35.51, 153.77) | 174.18 (85.23, 368.49) | 143.4 | 321.56 (155.29, 684.78) | 313.56 (153.61, 661.2) | -0.12 (-0.14, -0.11) |
| Andean Latin America | 47.31 (22.35, 102.11) | 151.02 (72.68, 318.35) | 219.21 | 278.53 (129.89, 603.92) | 272.02 (131.15, 573.64) | -0.11 (-0.12, -0.09) |
| Central Latin America | 220.4 (109.63, 471.32) | 783.86 (384.33, 1643.52) | 255.65 | 343.12 (166.95, 727.8) | 335.6 (163.8, 703.51) | -0.07 (-0.07, -0.06) |
| Tropical Latin America | 341.58 (159.91, 755) | 1226.23 (576.49, 2613.82) | 258.98 | 510.43 (234.39, 1109.41) | 503.17 (235.73, 1070.77) | -0.05 (-0.07, -0.04) |
| North Africa and Middle East | 578.98 (280.43, 1264.23) | 1566.07 (753.72, 3314.93) | 170.49 | 515.8 (245.62, 1112.57) | 476.29 (225.56, 1004.2) | -0.27 (-0.3, -0.25) |
| South Asia | 1009.43 (467.21, 2351.86) | 3450.14 (1531.59, 7711.44) | 241.79 | 272.02 (126.23, 615.5) | 308.27 (135.52, 684.96) | 0.4 (0.37, 0.43) |
| Central Sub-Saharan Africa | 62.7 (28.37, 141.97) | 178.4 (78.7, 408.1) | 184.51 | 535.4 (238.5, 1185.21) | 591.41 (255.67, 1360.26) | 0.36 (0.33, 0.4) |
| Eastern Sub-Saharan Africa | 191.45 (89.28, 426.49) | 488.06 (218.47, 1080.94) | 154.93 | 430.46 (196.38, 949.41) | 460.68 (200.73, 1028.27) | 0.24 (0.23, 0.25) |
| Southern Sub-Saharan Africa | 81.98 (39.19, 177.35) | 168.43 (77.83, 376.44) | 105.46 | 401.95 (189.45, 867.33) | 408.97 (187.4, 904.57) | 0.05 (0, 0.1) |
| Western Sub-Saharan Africa | 181.91 (82.65, 402.09) | 407.63 (181.17, 942.45) | 124.08 | 311.57 (138.94, 686.95) | 320.74 (138.35, 745.09) | 0.14 (0.1, 0.18) |
| Oceania | 6.68 (3.2, 14.22) | 17.63 (8.41, 38.11) | 163.83 | 427.58 (201.47, 930.31) | 397.82 (184.77, 863.92) | -0.28 (-0.3, -0.26) |
| Central Asia | 149.21 (71.67, 319.46) | 232.27 (112.59, 498.32) | 55.67 | 388.52 (183.29, 831.85) | 379.25 (181.2, 819.07) | -0.1 (-0.11, -0.09) |
| Central Europe | 488.56 (236.36, 1078.06) | 933.2 (446.02, 1970.59) | 91.01 | 391.81 (185.38, 853.38) | 386.04 (184.19, 812.17) | -0.05 (-0.06, -0.04) |
| East Asia | 2781.64 (1277.19, 6270.51) | 10359.13 (5080.42, 22833.68) | 272.41 | 527.37 (233.39, 1172.37) | 555.11 (267.58, 1222.86) | -0.02 (-0.06, 0.02) |
| Eastern Europe | 934.16 (452.8, 2040.93) | 1438.73 (673.86, 3104.95) | 54.01 | 403.75 (191.34, 870.95) | 396.72 (186.44, 853.77) | -0.07 (-0.09, -0.05) |
| High-income Asia Pacific | 795.71 (373.98, 1726.61) | 3028.73 (1439.61, 6015.52) | 280.63 | 470.24 (218.13, 1006.65) | 461.33 (222.39, 928.28) | 0.01 (-0.01, 0.04) |
| Australasia | 97.15 (47.03, 204.5) | 252.18 (117.59, 522.76) | 159.59 | 443.49 (212.56, 928.52) | 405.09 (190.47, 836.34) | -0.3 (-0.32, -0.29) |
| Western Europe | 2746.24 (1292.22, 5841.69) | 5385.86 (2539.41, 11019) | 96.12 | 460.18 (215.66, 971.82) | 443.24 (211.93, 909.88) | -0.12 (-0.13, -0.11) |
| Southeast Asia | 703.42 (344.01, 1485.73) | 2086.18 (973.51, 4362.2) | 196.58 | 400.2 (192.5, 839.35) | 418.63 (193.3, 883.66) | 0.12 (0.08, 0.15) |
| Southern Latin America | 150.66 (73.81, 321.85) | 340.21 (162.52, 709.45) | 125.81 | 379.33 (183.13, 803.78) | 368.23 (176.23, 766.73) | -0.08 (-0.1, -0.07) |

Note: CI, confidence interval; DALYs: disability adjusted life years; EAPC, estimated annual percentage change; GBD, Global Burden of Disease; SDI, socio-demographic index; UI, uncertainty interval

**Table S6**. **The deaths and age-standardized mortality rate for dementia and their temporal change at national level, 1990−2021**

|  | **Deaths (95% UI) per 1000 population** | | | **Age-standardized mortality rate (95% UI), per 100, 000 population** | | |
| --- | --- | --- | --- | --- | --- | --- |
| **Countries and territories** | **1990** | **2021** | **Percentage change (%)** | **1990** | **2021** | **EAPC (95% CI)** |
| Afghanistan | 1.26 (0.3, 3.4) | 1.62 (0.4, 4.29) | 28.24 | 35.31 (8.65, 95.86) | 33.27 (8.44, 89.55) | -0.2 (-0.23, -0.17) |
| Albania | 0.31 (0.08, 0.84) | 0.79 (0.19, 2.17) | 151.85 | 21.63 (5.36, 59.16) | 20.82 (4.95, 56.6) | -0.12 (-0.14, -0.09) |
| Algeria | 1.5 (0.35, 4.15) | 5.59 (1.38, 14.9) | 273.56 | 28.02 (6.74, 76.98) | 26.19 (6.43, 68.19) | -0.17 (-0.2, -0.14) |
| American Samoa | 0 (0, 0.01) | 0.01 (0, 0.02) | 171.16 | 24.16 (5.81, 66.64) | 22.47 (5.43, 60.53) | -0.17 (-0.22, -0.12) |
| Andorra | 0.01 (0, 0.02) | 0.05 (0.01, 0.12) | 378.36 | 25.55 (6.33, 63.33) | 24.08 (6.18, 64.18) | -0.12 (-0.16, -0.08) |
| Angola | 0.4 (0.09, 1.1) | 1.57 (0.37, 4.43) | 288.84 | 28.2 (6.57, 75.41) | 33.4 (8.06, 92.19) | 0.53 (0.48, 0.58) |
| Antigua and Barbuda | 0.01 (0, 0.03) | 0.01 (0, 0.03) | 25.39 | 16.71 (4.12, 45.6) | 15.78 (3.8, 44.13) | -0.07 (-0.11, -0.02) |
| Argentina | 5.22 (1.29, 14.32) | 12.02 (3.08, 31.56) | 130.48 | 20.88 (5.2, 56.06) | 20.21 (5.17, 53.04) | -0.06 (-0.07, -0.04) |
| Armenia | 0.39 (0.1, 1.05) | 0.9 (0.22, 2.39) | 133.08 | 19.98 (4.91, 54.39) | 21.34 (5.34, 56.32) | 0.3 (0.24, 0.36) |
| Australia | 4.1 (1, 10.98) | 12.85 (3.36, 32.38) | 213.23 | 24.2 (6.03, 63.96) | 22.89 (5.93, 57.75) | -0.16 (-0.18, -0.14) |
| Austria | 3.01 (0.74, 8.18) | 6.07 (1.57, 15.19) | 102.12 | 25.44 (6.22, 67.72) | 24.43 (6.28, 61.92) | -0.1 (-0.11, -0.09) |
| Azerbaijan | 0.72 (0.18, 2.04) | 1.29 (0.32, 3.6) | 78.03 | 21.19 (5.19, 58.29) | 20.54 (5.17, 57.09) | -0.04 (-0.08, 0.01) |
| Bahamas | 0.02 (0, 0.05) | 0.05 (0.01, 0.13) | 160.73 | 16.19 (3.87, 44.62) | 15.76 (3.78, 43.31) | -0.05 (-0.09, -0.02) |
| Bahrain | 0.02 (0, 0.04) | 0.07 (0.02, 0.2) | 368.78 | 29.27 (7.03, 80.92) | 25.61 (6.06, 72.79) | -0.49 (-0.56, -0.41) |
| Bangladesh | 4.77 (1.12, 13.19) | 16.56 (3.87, 49.23) | 247.32 | 15.4 (3.61, 43.17) | 17.27 (4.04, 50.94) | 0.29 (0.17, 0.4) |
| Barbados | 0.05 (0.01, 0.13) | 0.08 (0.02, 0.23) | 75.5 | 15.84 (3.71, 43.45) | 15.98 (3.78, 44.24) | 0.08 (0, 0.17) |
| Belarus | 2.33 (0.57, 6.49) | 3.42 (0.83, 9.38) | 46.34 | 21.03 (5.28, 57.99) | 20.58 (4.99, 56.43) | -0.1 (-0.11, -0.08) |
| Belgium | 4.42 (1.14, 11.61) | 8.83 (2.41, 21.58) | 99.6 | 29.12 (7.39, 75.81) | 26.47 (7.1, 65.12) | -0.27 (-0.33, -0.2) |
| Belize | 0.02 (0, 0.04) | 0.04 (0.01, 0.1) | 152.36 | 16.52 (4.04, 43.14) | 16.19 (3.93, 43.09) | 0.02 (-0.05, 0.09) |
| Benin | 0.24 (0.06, 0.64) | 0.55 (0.13, 1.49) | 124.92 | 17.98 (4.32, 48.5) | 18.44 (4.51, 49.83) | 0.09 (0.06, 0.12) |
| Bermuda | 0.01 (0, 0.02) | 0.03 (0.01, 0.07) | 231.9 | 17.02 (4.16, 46.25) | 16.45 (4.07, 42.53) | -0.1 (-0.14, -0.07) |
| Bhutan | 0.02 (0, 0.05) | 0.09 (0.02, 0.27) | 431.33 | 15.51 (3.62, 42.83) | 19.63 (4.83, 54.56) | 0.85 (0.79, 0.91) |
| Bolivia (Plurinational State of) | 0.29 (0.07, 0.77) | 0.93 (0.22, 2.55) | 223.01 | 15.49 (3.8, 40.1) | 15.65 (3.84, 41.49) | 0.05 (0.02, 0.08) |
| Bosnia and Herzegovina | 0.51 (0.12, 1.36) | 1.2 (0.29, 3.13) | 137.62 | 20.38 (5, 53.64) | 19.57 (4.76, 50.44) | -0.13 (-0.16, -0.1) |
| Botswana | 0.05 (0.01, 0.16) | 0.17 (0.04, 0.47) | 215.88 | 23.01 (5.35, 67.1) | 22.59 (5.43, 60.14) | 0 (-0.06, 0.07) |
| Brazil | 15.46 (3.83, 40.59) | 63.9 (16.79, 161.44) | 313.4 | 28.16 (7.35, 73.41) | 27.29 (7.15, 69.06) | -0.07 (-0.09, -0.06) |
| Brunei Darussalam | 0.01 (0, 0.04) | 0.03 (0.01, 0.09) | 170.96 | 22.44 (5.55, 61.65) | 23.27 (5.71, 62.03) | 0.23 (0.14, 0.31) |
| Bulgaria | 1.35 (0.31, 3.82) | 2.79 (0.71, 7.81) | 107.32 | 19.96 (4.68, 56.18) | 20.04 (5.05, 55.24) | -0.01 (-0.03, 0.02) |
| Burkina Faso | 0.46 (0.11, 1.28) | 1.02 (0.24, 2.85) | 121.64 | 22.14 (5.3, 60.77) | 20.27 (4.98, 55.69) | -0.35 (-0.41, -0.29) |
| Burundi | 0.27 (0.06, 0.74) | 0.56 (0.13, 1.58) | 110.84 | 22.55 (5.54, 61.04) | 25.05 (6.1, 69.42) | 0.38 (0.35, 0.41) |
| Cabo Verde | 0.04 (0.01, 0.1) | 0.07 (0.02, 0.19) | 91.76 | 16.53 (4.02, 43.18) | 17.54 (4.33, 46.61) | 0.15 (0.12, 0.19) |
| Cambodia | 0.46 (0.11, 1.31) | 1.69 (0.4, 4.89) | 264.52 | 21.16 (5.08, 58.17) | 25.75 (6.12, 71.34) | 0.74 (0.7, 0.79) |
| Cameroon | 0.47 (0.11, 1.26) | 1.18 (0.27, 3.39) | 152.58 | 20.46 (4.98, 54.92) | 19.25 (4.44, 54.68) | -0.24 (-0.3, -0.17) |
| Canada | 6.68 (1.7, 17.58) | 18.65 (4.96, 47.2) | 179.14 | 22.03 (5.7, 57.72) | 21.15 (5.57, 54) | -0.16 (-0.18, -0.14) |
| Central African Republic | 0.13 (0.03, 0.34) | 0.22 (0.05, 0.61) | 75.75 | 32.85 (7.99, 86.6) | 31.97 (7.5, 83.07) | -0.08 (-0.13, -0.04) |
| Chad | 0.3 (0.07, 0.83) | 0.52 (0.12, 1.44) | 73.05 | 17.19 (4.16, 46.35) | 17.76 (4.06, 48.36) | 0.14 (0.12, 0.16) |
| Chile | 1.44 (0.35, 3.93) | 5.2 (1.32, 13.39) | 259.93 | 19.5 (4.8, 51.9) | 19.41 (4.93, 49.99) | -0.02 (-0.04, 0) |
| China | 119.81 (28.35, 322.1) | 491.77 (124.97, 1330.18) | 310.47 | 31.39 (7.6, 83.63) | 30.82 (7.88, 82.43) | -0.19 (-0.23, -0.15) |
| Colombia | 2.23 (0.55, 6.06) | 11.04 (2.81, 27.27) | 395.2 | 17.98 (4.49, 48.96) | 17.86 (4.53, 44.77) | -0.03 (-0.05, -0.02) |
| Comoros | 0.02 (0.01, 0.06) | 0.08 (0.02, 0.23) | 279.46 | 25 (6.06, 64.94) | 28.16 (7.02, 75.17) | 0.42 (0.4, 0.45) |
| Congo | 0.13 (0.03, 0.35) | 0.38 (0.09, 1.01) | 180.91 | 34.9 (8.36, 91.71) | 34.49 (8.41, 89.94) | -0.03 (-0.05, 0) |
| Cook Islands | 0 (0, 0.01) | 0.01 (0, 0.01) | 177.52 | 25.23 (6.12, 70.58) | 21.9 (5.71, 57.63) | -0.42 (-0.45, -0.38) |
| Costa Rica | 0.28 (0.07, 0.75) | 1.08 (0.28, 2.75) | 288.26 | 18.44 (4.61, 50.01) | 18.04 (4.54, 46.4) | -0.06 (-0.08, -0.04) |
| Cote d'Ivoire | 0.29 (0.07, 0.78) | 0.99 (0.22, 2.76) | 236.97 | 19.4 (4.8, 51.5) | 19.1 (4.39, 52.57) | -0.04 (-0.07, -0.01) |
| Croatia | 0.92 (0.22, 2.59) | 2.05 (0.49, 5.48) | 122.03 | 20.87 (5.08, 57.2) | 20.34 (4.92, 53.88) | -0.09 (-0.12, -0.07) |
| Cuba | 1.32 (0.32, 3.71) | 3.5 (0.86, 9.6) | 163.94 | 15.8 (3.79, 42.99) | 15.4 (3.82, 42.87) | -0.11 (-0.13, -0.1) |
| Cyprus | 0.12 (0.03, 0.35) | 0.44 (0.11, 1.21) | 257.58 | 28.94 (6.93, 81.14) | 25.81 (6.31, 69.51) | -0.37 (-0.44, -0.3) |
| Czechia | 2.39 (0.57, 6.7) | 4.9 (1.21, 12.87) | 105.16 | 20.56 (5.07, 56) | 20.37 (5.02, 53.57) | 0.02 (0, 0.04) |
| Democratic People's Republic of Korea | 1.97 (0.45, 5.46) | 5.83 (1.34, 16.38) | 195.65 | 23.32 (5.35, 64.29) | 24.21 (5.69, 67.83) | 0.21 (0.17, 0.26) |
| Democratic Republic of the Congo | 1.7 (0.41, 4.67) | 5.67 (1.35, 15.43) | 233.41 | 30.13 (7.3, 80.02) | 35.44 (8.35, 93.78) | 0.62 (0.55, 0.69) |
| Denmark | 2.05 (0.52, 5.57) | 3.51 (0.9, 8.82) | 71.43 | 23.22 (5.78, 62.26) | 24.12 (6.14, 60.94) | 0.1 (0.01, 0.18) |
| Djibouti | 0.01 (0, 0.03) | 0.07 (0.02, 0.18) | 447.32 | 26.14 (6.07, 70.91) | 28.76 (6.68, 76.15) | 0.31 (0.27, 0.34) |
| Dominica | 0.01 (0, 0.02) | 0.01 (0, 0.03) | 34.9 | 16.67 (4.07, 46.25) | 16.46 (4.04, 43.8) | -0.01 (-0.04, 0.02) |
| Dominican Republic | 0.41 (0.1, 1.14) | 1.53 (0.36, 4.03) | 271.74 | 17.1 (4.03, 48.37) | 16.39 (3.85, 43.31) | -0.03 (-0.08, 0.02) |
| Ecuador | 0.57 (0.13, 1.56) | 1.95 (0.44, 5.44) | 242.18 | 14.53 (3.4, 39.17) | 13.95 (3.22, 38.79) | -0.11 (-0.13, -0.09) |
| Egypt | 3.07 (0.73, 8.59) | 6.5 (1.56, 17.7) | 111.31 | 27.08 (6.42, 77.65) | 24.37 (5.88, 66.23) | -0.38 (-0.4, -0.35) |
| El Salvador | 0.52 (0.13, 1.35) | 1.41 (0.36, 3.51) | 170.76 | 18.68 (4.71, 48.91) | 18.28 (4.54, 46.04) | -0.07 (-0.09, -0.04) |
| Equatorial Guinea | 0.02 (0.01, 0.06) | 0.08 (0.02, 0.21) | 233.89 | 29.58 (7.12, 80.48) | 33.05 (7.47, 86.51) | 0.4 (0.32, 0.48) |
| Eritrea | 0.07 (0.02, 0.21) | 0.3 (0.07, 0.84) | 313.25 | 23.99 (5.81, 66.86) | 30.04 (7.02, 80.75) | 0.73 (0.65, 0.81) |
| Estonia | 0.36 (0.09, 1.01) | 0.78 (0.19, 2.06) | 119.69 | 20.76 (4.96, 57.16) | 21.97 (5.44, 57.14) | 0.19 (0.17, 0.2) |
| Eswatini | 0.03 (0.01, 0.09) | 0.05 (0.01, 0.16) | 68.75 | 23.35 (5.75, 64.74) | 23.38 (5.48, 63.37) | 0.18 (0.13, 0.23) |
| Ethiopia | 1.89 (0.44, 5.27) | 7.41 (1.81, 19.59) | 291.52 | 26.85 (6.56, 74.38) | 28.67 (7.39, 74.05) | 0.23 (0.15, 0.31) |
| Fiji | 0.04 (0.01, 0.1) | 0.08 (0.02, 0.21) | 114.79 | 23.56 (5.67, 67.01) | 22.54 (5.7, 59.17) | -0.21 (-0.25, -0.18) |
| Finland | 1.9 (0.49, 5.04) | 4.59 (1.23, 11.24) | 141.51 | 28.12 (7.3, 73.19) | 26.14 (7, 64.65) | -0.18 (-0.22, -0.15) |
| France | 20.5 (5.12, 54.43) | 46.57 (12.13, 116.35) | 127.2 | 22.98 (5.79, 60.15) | 21.69 (5.61, 54.3) | -0.18 (-0.2, -0.17) |
| Gabon | 0.12 (0.03, 0.32) | 0.19 (0.04, 0.51) | 56.47 | 35.33 (8.96, 91.15) | 35.44 (8.87, 93.89) | 0.01 (0, 0.01) |
| Gambia | 0.03 (0.01, 0.09) | 0.11 (0.03, 0.32) | 261.29 | 19.76 (4.77, 53.04) | 20.15 (4.74, 54.1) | 0 (-0.05, 0.04) |
| Georgia | 1.01 (0.25, 2.81) | 1.57 (0.41, 4.01) | 54.86 | 20.59 (5.04, 56.28) | 21.11 (5.51, 55.26) | 0.09 (0.01, 0.17) |
| Germany | 37.43 (9.42, 94.97) | 78.58 (21.42, 186.71) | 109.9 | 29.5 (7.52, 73.8) | 29.25 (7.91, 69.29) | -0.07 (-0.12, -0.03) |
| Ghana | 0.48 (0.11, 1.39) | 1.59 (0.37, 4.44) | 228.59 | 16.74 (4.02, 46.59) | 19.38 (4.6, 54.77) | 0.5 (0.45, 0.55) |
| Greece | 3.21 (0.79, 8.85) | 8.93 (2.26, 22.81) | 177.73 | 24.85 (6.22, 66.57) | 24.23 (6.08, 61.76) | -0.09 (-0.11, -0.08) |
| Greenland | 0 (0, 0.01) | 0.01 (0, 0.02) | 162.78 | 26.47 (6.28, 71.42) | 25.68 (6.77, 65.52) | -0.06 (-0.09, -0.03) |
| Grenada | 0.02 (0, 0.04) | 0.01 (0, 0.04) | -21.51 | 17.7 (4.2, 47.39) | 16.75 (3.95, 46.67) | 0.17 (0.07, 0.26) |
| Guam | 0.01 (0, 0.02) | 0.05 (0.01, 0.11) | 552.43 | 23.14 (5.54, 63.24) | 18.31 (5.14, 42.9) | -0.7 (-0.76, -0.64) |
| Guatemala | 0.32 (0.08, 0.9) | 1.53 (0.38, 4.11) | 372.67 | 18.9 (4.49, 51.79) | 18.29 (4.47, 49.71) | -0.13 (-0.16, -0.11) |
| Guinea | 0.36 (0.09, 1) | 0.65 (0.15, 1.8) | 80.64 | 17.04 (4.13, 46.39) | 18.5 (4.17, 50.59) | 0.31 (0.28, 0.34) |
| Guinea-Bissau | 0.03 (0.01, 0.09) | 0.05 (0.01, 0.15) | 74.63 | 18.9 (4.65, 51.96) | 19.74 (4.59, 54.93) | 0.17 (0.14, 0.2) |
| Guyana | 0.04 (0.01, 0.1) | 0.06 (0.01, 0.18) | 71.77 | 15.25 (3.67, 41.62) | 15.56 (3.64, 41.96) | 0.01 (-0.09, 0.12) |
| Haiti | 0.26 (0.06, 0.71) | 0.59 (0.13, 1.71) | 130.09 | 17.13 (4.07, 48.43) | 16.49 (3.62, 46.24) | -0.1 (-0.11, -0.09) |
| Honduras | 0.26 (0.06, 0.7) | 0.91 (0.22, 2.53) | 246.4 | 20.35 (5, 54.04) | 22.97 (5.57, 62.96) | 0.36 (0.27, 0.44) |
| Hungary | 2.36 (0.57, 6.64) | 4.56 (1.11, 12.11) | 93.22 | 20.73 (5.04, 57.34) | 20.1 (4.89, 53.41) | -0.06 (-0.07, -0.05) |
| Iceland | 0.08 (0.02, 0.2) | 0.18 (0.05, 0.45) | 132.41 | 24.68 (6.16, 64.12) | 24.3 (6.45, 60.39) | 0 (-0.05, 0.05) |
| India | 30.32 (6.85, 88.8) | 133.29 (31.38, 367.63) | 339.54 | 13.35 (3.01, 38.49) | 16.98 (4.05, 46.35) | 0.89 (0.83, 0.95) |
| Indonesia | 8.98 (2.11, 25.13) | 26.28 (6.33, 72.88) | 192.75 | 17.55 (4.1, 47.58) | 22.97 (5.62, 63.06) | 0.77 (0.68, 0.87) |
| Iran (Islamic Republic of) | 3.41 (0.83, 8.93) | 14.94 (3.72, 39.57) | 337.67 | 27.3 (6.77, 73.62) | 25.18 (6.28, 66.26) | -0.29 (-0.31, -0.27) |
| Iraq | 1.77 (0.44, 4.6) | 3.31 (0.83, 8.83) | 86.74 | 26.16 (6.43, 67.77) | 25.77 (6.39, 66.8) | -0.16 (-0.19, -0.12) |
| Ireland | 0.86 (0.21, 2.34) | 2.12 (0.55, 5.3) | 146.5 | 25.29 (6.23, 67.37) | 24.09 (6.25, 60.28) | -0.09 (-0.12, -0.07) |
| Israel | 0.95 (0.24, 2.6) | 3.61 (0.95, 8.83) | 281.28 | 24.43 (6.15, 64.85) | 24.29 (6.31, 60.01) | -0.02 (-0.05, 0.02) |
| Italy | 25.6 (6.51, 67.62) | 64.76 (17.89, 156.72) | 152.98 | 31.5 (8.08, 81.26) | 29.75 (8.1, 72.88) | -0.09 (-0.13, -0.05) |
| Jamaica | 0.31 (0.07, 0.84) | 0.65 (0.17, 1.69) | 111.82 | 16.42 (3.99, 45.09) | 16.02 (4, 43.04) | -0.09 (-0.12, -0.06) |
| Japan | 36.97 (9.42, 96.17) | 172.81 (50.41, 398.75) | 367.39 | 27.18 (7.14, 69.33) | 26.33 (7.39, 62.16) | -0.07 (-0.1, -0.05) |
| Jordan | 0.19 (0.05, 0.49) | 0.91 (0.22, 2.49) | 393.58 | 26.24 (6.72, 70.1) | 23.37 (5.78, 64.07) | -0.43 (-0.46, -0.4) |
| Kazakhstan | 1.89 (0.46, 5.07) | 2.14 (0.51, 6.15) | 13.14 | 20.79 (5.15, 55.93) | 19.44 (4.58, 55.94) | -0.33 (-0.38, -0.27) |
| Kenya | 1.1 (0.26, 3.12) | 3.15 (0.75, 8.1) | 184.89 | 23.88 (5.75, 66.29) | 28.7 (7.01, 72.33) | 0.67 (0.65, 0.7) |
| Kiribati | 0 (0, 0.01) | 0.01 (0, 0.02) | 107.98 | 23.75 (5.39, 63.95) | 27.39 (6.35, 72.65) | 0.46 (0.42, 0.5) |
| Kuwait | 0.08 (0.02, 0.21) | 0.47 (0.12, 1.22) | 462.87 | 26.98 (6.93, 67.44) | 24.36 (6.33, 63.3) | -0.36 (-0.47, -0.24) |
| Kyrgyzstan | 0.47 (0.12, 1.28) | 0.63 (0.16, 1.63) | 33.39 | 21.24 (5.22, 56.57) | 20.09 (5.07, 52.52) | -0.21 (-0.24, -0.19) |
| Lao People's Democratic Republic | 0.17 (0.04, 0.5) | 0.55 (0.13, 1.53) | 215.53 | 19.59 (4.6, 54.66) | 21.6 (5.14, 60.21) | 0.36 (0.32, 0.4) |
| Latvia | 0.66 (0.16, 1.86) | 1.05 (0.26, 2.84) | 59.71 | 20.83 (5.03, 57.58) | 20.79 (5.08, 55.96) | 0.02 (-0.01, 0.04) |
| Lebanon | 0.35 (0.09, 0.94) | 1.66 (0.42, 4.33) | 378.75 | 24.7 (6.34, 67.28) | 23.83 (6.08, 62.44) | -0.05 (-0.17, 0.06) |
| Lesotho | 0.12 (0.03, 0.32) | 0.12 (0.03, 0.33) | 0.33 | 22.14 (5.27, 60.07) | 23.44 (5.96, 65.49) | 0.4 (0.29, 0.51) |
| Liberia | 0.11 (0.03, 0.31) | 0.2 (0.04, 0.56) | 79.96 | 17.77 (4.21, 50.37) | 17.86 (4.03, 49.79) | 0.07 (0.04, 0.1) |
| Libya | 0.47 (0.12, 1.16) | 0.99 (0.24, 2.66) | 112.47 | 30.8 (8.02, 76.76) | 28.04 (6.73, 75.56) | -0.21 (-0.26, -0.16) |
| Lithuania | 0.86 (0.21, 2.4) | 1.55 (0.38, 4.19) | 79.76 | 20.74 (4.96, 57.05) | 21.02 (5.2, 56.8) | 0.04 (0.03, 0.06) |
| Luxembourg | 0.08 (0.02, 0.23) | 0.23 (0.06, 0.58) | 176.43 | 17.61 (4.2, 47.35) | 17.72 (4.55, 44.71) | 0.06 (0.02, 0.1) |
| Madagascar | 0.51 (0.12, 1.41) | 0.86 (0.2, 2.32) | 68.47 | 18.82 (4.53, 52.84) | 19.74 (4.71, 52.59) | 0.16 (0.14, 0.18) |
| Malawi | 0.4 (0.1, 1.15) | 0.96 (0.22, 2.6) | 137.08 | 23.75 (5.81, 68.09) | 27.07 (6.32, 73.02) | 0.49 (0.45, 0.53) |
| Malaysia | 1.72 (0.45, 4.48) | 4.72 (1.15, 12.89) | 173.57 | 23.63 (6.21, 62.13) | 24.39 (6.02, 65.59) | 0.02 (-0.05, 0.1) |
| Maldives | 0.01 (0, 0.02) | 0.05 (0.01, 0.13) | 619.7 | 17.74 (4.14, 50.44) | 19.55 (4.8, 52.57) | 0.3 (0.21, 0.38) |
| Mali | 0.38 (0.09, 1.03) | 0.9 (0.22, 2.54) | 135.27 | 22.03 (5.35, 60.22) | 21.85 (5.18, 61.16) | 0.04 (0, 0.08) |
| Malta | 0.08 (0.02, 0.23) | 0.29 (0.08, 0.71) | 250.72 | 24.71 (6.13, 65.73) | 24 (6.24, 59.13) | -0.17 (-0.2, -0.13) |
| Marshall Islands | 0 (0, 0.01) | 0 (0, 0.01) | 49.12 | 25.92 (6.15, 72.44) | 24.17 (5.79, 69.03) | -0.27 (-0.28, -0.25) |
| Mauritania | 0.11 (0.03, 0.3) | 0.27 (0.06, 0.78) | 151.72 | 18.8 (4.37, 52.5) | 19.08 (4.23, 56.09) | -0.02 (-0.04, 0.01) |
| Mauritius | 0.09 (0.02, 0.25) | 0.32 (0.08, 0.87) | 252.03 | 21.92 (5.34, 58.62) | 20.3 (5, 55.3) | -0.31 (-0.39, -0.22) |
| Mexico | 4.2 (1, 11.88) | 15.55 (3.65, 43.85) | 269.9 | 15.1 (3.65, 41.69) | 14.8 (3.49, 41.39) | -0.07 (-0.09, -0.06) |
| Micronesia (Federated States of) | 0.01 (0, 0.02) | 0.01 (0, 0.02) | 23.52 | 26.52 (6.41, 74.67) | 26.24 (6.29, 73.73) | -0.03 (-0.07, 0.01) |
| Monaco | 0.02 (0.01, 0.06) | 0.04 (0.01, 0.09) | 70.31 | 25.82 (6.09, 70.75) | 27.22 (6.93, 64.58) | 0.2 (0.17, 0.24) |
| Mongolia | 0.15 (0.04, 0.39) | 0.25 (0.06, 0.68) | 69.34 | 22.65 (5.65, 59.43) | 21.66 (5.32, 59.88) | -0.25 (-0.29, -0.21) |
| Montenegro | 0.11 (0.03, 0.29) | 0.15 (0.04, 0.41) | 40.23 | 20.59 (4.9, 55.78) | 20.47 (5.01, 54.91) | 0 (-0.06, 0.06) |
| Morocco | 2.62 (0.64, 7) | 6.09 (1.46, 16.45) | 132.45 | 26.47 (6.53, 70.71) | 26.13 (6.16, 70.4) | 0.01 (-0.02, 0.04) |
| Mozambique | 0.72 (0.18, 1.99) | 1.44 (0.34, 4.01) | 99.89 | 26.85 (6.81, 72.77) | 29.76 (7.04, 83.45) | 0.49 (0.44, 0.54) |
| Myanmar | 2.18 (0.51, 5.91) | 6.86 (1.57, 19.63) | 214.09 | 19.06 (4.6, 51.48) | 21.5 (5.04, 60.71) | 0.36 (0.32, 0.41) |
| Namibia | 0.06 (0.01, 0.18) | 0.18 (0.04, 0.51) | 178.39 | 21.69 (5.39, 62.32) | 24.26 (5.55, 69.21) | 0.39 (0.36, 0.42) |
| Nauru | 0 (0, 0) | 0 (0, 0) | 51.07 | 25.22 (5.94, 69.48) | 25.19 (5.89, 71.7) | 0.04 (0.01, 0.06) |
| Nepal | 0.66 (0.15, 1.86) | 2.49 (0.57, 6.98) | 279.82 | 13.97 (3.22, 38.57) | 17.32 (4.03, 48.09) | 0.77 (0.71, 0.84) |
| Netherlands | 5.68 (1.45, 14.43) | 11.65 (3.14, 29.3) | 105.08 | 28.41 (7.26, 71.5) | 27.94 (7.49, 70.46) | -0.03 (-0.05, -0.01) |
| New Zealand | 0.9 (0.23, 2.4) | 2.4 (0.64, 6.17) | 166.38 | 26.12 (6.67, 68.2) | 24.83 (6.58, 64) | -0.19 (-0.23, -0.14) |
| Nicaragua | 0.2 (0.05, 0.52) | 0.67 (0.16, 1.78) | 227.87 | 18.28 (4.67, 47.36) | 17.57 (4.39, 46.45) | -0.08 (-0.12, -0.05) |
| Niger | 0.21 (0.05, 0.56) | 0.72 (0.17, 1.97) | 240.22 | 18.87 (4.68, 50.47) | 19.32 (4.53, 52.21) | 0.18 (0.12, 0.24) |
| Nigeria | 4.32 (1.07, 12.02) | 9.54 (2.3, 25.99) | 120.85 | 17.36 (4.33, 47.96) | 19.01 (4.63, 51.88) | 0.43 (0.35, 0.5) |
| Niue | 0 (0, 0) | 0 (0, 0) | -35.26 | 27.77 (6.79, 75.94) | 24.96 (6.15, 65.56) | -0.37 (-0.39, -0.35) |
| North Macedonia | 0.28 (0.07, 0.76) | 0.37 (0.09, 1.04) | 34.59 | 19.21 (4.79, 52.96) | 19.67 (4.83, 54.02) | 0.02 (-0.03, 0.06) |
| Northern Mariana Islands | 0 (0, 0) | 0.01 (0, 0.02) | 351.44 | 23.33 (5.66, 64.94) | 22.81 (5.53, 62.38) | -0.05 (-0.11, 0) |
| Norway | 2.08 (0.53, 5.46) | 3.24 (0.86, 8.13) | 55.96 | 26.54 (6.74, 69.38) | 24.87 (6.46, 63.48) | -0.21 (-0.26, -0.16) |
| Oman | 0.1 (0.02, 0.28) | 0.23 (0.06, 0.64) | 121.27 | 28.27 (6.99, 74.05) | 26.12 (6.65, 71.88) | -0.24 (-0.37, -0.11) |
| Pakistan | 6.02 (1.46, 17.09) | 12.94 (3.05, 35.77) | 114.78 | 17.08 (4.08, 48.08) | 19.59 (4.73, 53.82) | 0.36 (0.31, 0.41) |
| Palau | 0 (0, 0) | 0 (0, 0.01) | 78.6 | 24.72 (6.01, 68.55) | 21.7 (5.15, 62.95) | -0.39 (-0.44, -0.34) |
| Palestine | 0.15 (0.04, 0.42) | 0.33 (0.08, 0.93) | 116.26 | 26.68 (6.42, 73.99) | 24.68 (6.05, 67.81) | -0.25 (-0.32, -0.19) |
| Panama | 0.22 (0.05, 0.59) | 0.86 (0.21, 2.17) | 290.31 | 17.53 (4.34, 46.56) | 17.6 (4.37, 44.67) | 0.04 (0.01, 0.07) |
| Papua New Guinea | 0.14 (0.03, 0.38) | 0.44 (0.1, 1.25) | 223.33 | 21.26 (5.2, 58.69) | 20.56 (4.68, 59.98) | -0.15 (-0.17, -0.14) |
| Paraguay | 0.46 (0.11, 1.19) | 1.33 (0.34, 3.38) | 188.43 | 24.93 (6.03, 64.42) | 25.62 (6.4, 65.43) | 0.13 (0.11, 0.16) |
| Peru | 1.44 (0.35, 3.91) | 4.79 (1.23, 12.68) | 233.05 | 14.42 (3.48, 39.42) | 13.9 (3.49, 37.12) | -0.17 (-0.21, -0.13) |
| Philippines | 2.97 (0.72, 8.31) | 11.05 (2.64, 27.7) | 272.49 | 20.02 (4.85, 56.29) | 21.49 (5.23, 54.38) | 0.29 (0.26, 0.32) |
| Poland | 7.4 (1.81, 20.75) | 17.16 (4.36, 44.66) | 131.89 | 21.47 (5.29, 58.89) | 21.08 (5.36, 55.12) | -0.05 (-0.07, -0.04) |
| Portugal | 2.72 (0.65, 7.69) | 8.56 (2.25, 21.4) | 215.09 | 25.65 (6.17, 70.72) | 24.79 (6.47, 62.09) | -0.09 (-0.12, -0.07) |
| Puerto Rico | 0.51 (0.12, 1.45) | 1.75 (0.44, 4.37) | 243.26 | 16.88 (4.05, 46.86) | 15.96 (4.06, 40.88) | -0.18 (-0.2, -0.16) |
| Qatar | 0.01 (0, 0.03) | 0.06 (0.02, 0.18) | 572.73 | 29.97 (7.17, 82.3) | 26.54 (6.57, 73.95) | -0.41 (-0.47, -0.34) |
| Republic of Korea | 4.93 (1.23, 12.87) | 25.86 (6.92, 62.63) | 425.03 | 35.68 (9.05, 90) | 29.02 (7.87, 69.71) | -0.53 (-0.59, -0.47) |
| Republic of Moldova | 0.54 (0.13, 1.5) | 1.2 (0.3, 3.19) | 121.97 | 20.76 (4.93, 57.7) | 19.02 (4.72, 50.98) | -0.29 (-0.32, -0.26) |
| Romania | 3.59 (0.86, 10.25) | 8.54 (2.06, 23.02) | 137.96 | 20.17 (4.82, 55.77) | 20.06 (4.83, 53.48) | -0.03 (-0.04, -0.02) |
| Russian Federation | 26.9 (6.59, 75.55) | 50.93 (12.58, 141.82) | 89.33 | 21.62 (5.27, 59.64) | 21.05 (5.19, 58.31) | -0.09 (-0.1, -0.08) |
| Rwanda | 0.29 (0.07, 0.81) | 0.89 (0.22, 2.4) | 205.88 | 25 (6.26, 69.32) | 29.4 (7.1, 78.95) | 0.56 (0.54, 0.59) |
| Saint Kitts and Nevis | 0 (0, 0.01) | 0.01 (0, 0.02) | 44.7 | 16.15 (3.76, 45.57) | 15.52 (3.73, 42.23) | 0.03 (-0.03, 0.1) |
| Saint Lucia | 0.01 (0, 0.03) | 0.04 (0.01, 0.1) | 252.6 | 16.63 (3.9, 46.02) | 16.4 (3.92, 45) | -0.14 (-0.18, -0.11) |
| Saint Vincent and the Grenadines | 0.01 (0, 0.03) | 0.02 (0, 0.05) | 89.01 | 17.79 (4.27, 48.01) | 15.86 (3.81, 43.79) | -0.23 (-0.31, -0.15) |
| Samoa | 0.01 (0, 0.04) | 0.03 (0.01, 0.07) | 79.39 | 27.74 (6.81, 74.15) | 26.49 (6.49, 74.06) | -0.15 (-0.21, -0.1) |
| San Marino | 0.01 (0, 0.02) | 0.03 (0.01, 0.06) | 197.63 | 23.65 (6.08, 60.98) | 21.49 (5.71, 51.92) | -0.2 (-0.27, -0.13) |
| Sao Tome and Principe | 0.01 (0, 0.02) | 0.01 (0, 0.03) | 39.81 | 17.81 (4.11, 49.62) | 17.68 (4.41, 47.61) | -0.01 (-0.07, 0.05) |
| Saudi Arabia | 0.93 (0.22, 2.46) | 1.7 (0.41, 4.71) | 81.7 | 28.48 (6.96, 74.02) | 25.85 (6.36, 71.39) | -0.36 (-0.39, -0.33) |
| Senegal | 0.34 (0.08, 0.95) | 0.97 (0.22, 2.71) | 184.47 | 18.91 (4.71, 50.97) | 20.86 (4.67, 57.98) | 0.3 (0.27, 0.32) |
| Serbia | 1.43 (0.33, 4.08) | 3.48 (0.86, 9.31) | 143.7 | 20.31 (4.77, 57.47) | 19.91 (4.9, 52.96) | -0.15 (-0.19, -0.12) |
| Seychelles | 0.01 (0, 0.03) | 0.02 (0, 0.05) | 51.66 | 22.01 (5.49, 61.18) | 20.76 (4.98, 56.32) | -0.15 (-0.18, -0.13) |
| Sierra Leone | 0.21 (0.05, 0.6) | 0.36 (0.09, 1.01) | 68.54 | 16.94 (3.86, 46.86) | 17.08 (4.1, 47.04) | 0.02 (0, 0.05) |
| Singapore | 0.25 (0.06, 0.66) | 1.46 (0.39, 3.57) | 478.93 | 18.8 (4.79, 49.2) | 17.9 (4.76, 44.1) | -0.08 (-0.11, -0.04) |
| Slovakia | 1.04 (0.25, 2.9) | 1.93 (0.49, 5.07) | 85.87 | 20.91 (5.16, 57.44) | 20.67 (5.22, 54.33) | 0.02 (-0.02, 0.05) |
| Slovenia | 0.45 (0.11, 1.22) | 1.22 (0.31, 3.08) | 172.55 | 20.17 (4.97, 53.7) | 20.8 (5.25, 51.88) | 0.12 (0.06, 0.18) |
| Solomon Islands | 0.01 (0, 0.03) | 0.04 (0.01, 0.1) | 218.41 | 23.45 (5.5, 69.31) | 23.63 (5.5, 67.3) | 0.02 (-0.01, 0.04) |
| Somalia | 0.2 (0.05, 0.55) | 0.49 (0.11, 1.38) | 142.79 | 22.73 (5.37, 62.88) | 24.59 (5.68, 70.98) | 0.44 (0.38, 0.51) |
| South Africa | 3.12 (0.76, 8.45) | 6.67 (1.62, 18.63) | 113.67 | 21.16 (5.19, 57.34) | 22.58 (5.4, 61.8) | 0.18 (0.1, 0.26) |
| South Sudan | 0.31 (0.07, 0.84) | 0.44 (0.1, 1.19) | 40.58 | 23.1 (5.34, 61.24) | 23.56 (5.5, 63.58) | 0.05 (0.01, 0.09) |
| Spain | 13.33 (3.28, 34.81) | 36.16 (9.67, 88.01) | 171.18 | 26.55 (6.65, 68.37) | 24.86 (6.56, 61.21) | -0.17 (-0.19, -0.15) |
| Sri Lanka | 1.14 (0.27, 3.16) | 3.83 (0.87, 10.18) | 235.05 | 18.87 (4.43, 51.85) | 18.92 (4.4, 49.86) | 0.03 (-0.01, 0.07) |
| Sudan | 1.26 (0.3, 3.5) | 2.77 (0.67, 7.5) | 119.31 | 25.22 (6, 70.09) | 23.36 (5.68, 62.7) | -0.27 (-0.29, -0.24) |
| Suriname | 0.04 (0.01, 0.1) | 0.09 (0.02, 0.24) | 147.98 | 16.58 (4.06, 44.78) | 16.3 (4.12, 43.02) | -0.04 (-0.08, 0) |
| Sweden | 4.2 (1.07, 11) | 6.92 (1.83, 17.51) | 64.91 | 24.65 (6.34, 63.75) | 23.2 (6.05, 59.29) | -0.12 (-0.18, -0.07) |
| Switzerland | 2.88 (0.73, 7.54) | 6.36 (1.74, 15.34) | 120.55 | 24.88 (6.22, 64.3) | 24.63 (6.67, 60.24) | -0.04 (-0.14, 0.06) |
| Syrian Arab Republic | 0.92 (0.22, 2.47) | 1.69 (0.39, 4.76) | 85.11 | 25.32 (6.15, 69.13) | 23.83 (5.62, 66.4) | -0.21 (-0.24, -0.19) |
| Taiwan (Province of China) | 1.63 (0.39, 4.43) | 10.05 (2.64, 25.17) | 514.95 | 20.2 (5.01, 53.73) | 21.13 (5.44, 53.08) | -0.24 (-0.39, -0.08) |
| Tajikistan | 0.44 (0.11, 1.22) | 0.67 (0.16, 1.79) | 52.06 | 20.91 (5.16, 57.22) | 21.59 (5.44, 58.06) | 0.08 (0.04, 0.12) |
| Thailand | 4.88 (1.2, 12.84) | 23.49 (6.11, 60.96) | 381.79 | 22.17 (5.37, 58.87) | 21.11 (5.47, 54.92) | -0.34 (-0.41, -0.28) |
| Timor-Leste | 0.03 (0.01, 0.07) | 0.11 (0.03, 0.31) | 319.63 | 18.75 (4.45, 53.28) | 20.93 (5, 59.12) | 0.42 (0.38, 0.46) |
| Togo | 0.11 (0.03, 0.31) | 0.32 (0.07, 0.92) | 193.31 | 18.55 (4.34, 50.51) | 20.09 (4.63, 57.3) | 0.26 (0.22, 0.29) |
| Tokelau | 0 (0, 0) | 0 (0, 0) | 30.47 | 28.32 (6.98, 78.23) | 25.39 (6.23, 67.04) | -0.38 (-0.39, -0.37) |
| Tonga | 0.01 (0, 0.02) | 0.02 (0, 0.05) | 104.86 | 26.42 (6.5, 71.51) | 25.89 (6.42, 67.51) | -0.01 (-0.03, 0.01) |
| Trinidad and Tobago | 0.09 (0.02, 0.26) | 0.28 (0.07, 0.74) | 204.55 | 16.66 (3.98, 45.8) | 15.74 (3.87, 42.35) | -0.1 (-0.14, -0.07) |
| Tunisia | 0.74 (0.18, 2.03) | 2.7 (0.66, 7.08) | 264.77 | 28.25 (6.9, 75.36) | 26.02 (6.42, 67.84) | -0.28 (-0.3, -0.26) |
| Turkey | 6.87 (1.71, 18.08) | 20 (5.05, 52.55) | 191.32 | 30.08 (7.54, 79.27) | 26.16 (6.61, 69.03) | -0.45 (-0.58, -0.33) |
| Turkmenistan | 0.25 (0.06, 0.68) | 0.57 (0.14, 1.59) | 124.88 | 21 (5.19, 56.74) | 19.71 (4.78, 54.98) | -0.32 (-0.37, -0.27) |
| Tuvalu | 0 (0, 0) | 0 (0, 0) | 111.63 | 26.98 (6.56, 77.12) | 25.84 (6.14, 72.71) | -0.18 (-0.19, -0.16) |
| Uganda | 0.71 (0.18, 1.95) | 2.06 (0.5, 5.67) | 191.83 | 22.03 (5.53, 58.86) | 26.87 (6.75, 71.15) | 0.7 (0.65, 0.74) |
| Ukraine | 11.53 (2.78, 33.3) | 16.38 (3.94, 44.76) | 42.12 | 21.5 (5.16, 60.47) | 21.01 (5.07, 57.17) | -0.09 (-0.11, -0.08) |
| United Arab Emirates | 0.04 (0.01, 0.12) | 0.16 (0.04, 0.45) | 257.65 | 28.84 (7.07, 74.4) | 24.18 (6.18, 63.07) | -0.37 (-0.5, -0.24) |
| United Kingdom | 21.69 (5.41, 58.2) | 37.56 (9.84, 94.97) | 73.18 | 23.69 (5.96, 62.48) | 22.61 (5.88, 57.62) | -0.09 (-0.13, -0.05) |
| United Republic of Tanzania | 1.32 (0.31, 3.65) | 3.85 (0.94, 10.34) | 192.66 | 25.72 (6.3, 68.21) | 25.84 (6.63, 68.55) | 0 (-0.03, 0.02) |
| United States of America | 99.96 (25.91, 258.21) | 198.05 (53.3, 494.05) | 98.13 | 29.64 (7.72, 76.08) | 29.17 (7.74, 73.52) | -0.08 (-0.09, -0.06) |
| United States Virgin Islands | 0.01 (0, 0.02) | 0.03 (0.01, 0.07) | 206.14 | 17.44 (4.33, 47.57) | 15.2 (3.67, 40.13) | -0.4 (-0.42, -0.38) |
| Uruguay | 0.74 (0.18, 2.06) | 1.57 (0.41, 4.05) | 111.64 | 20.7 (5.04, 56.48) | 21.17 (5.41, 54.56) | 0.08 (0.06, 0.09) |
| Uzbekistan | 1.93 (0.48, 5.27) | 3 (0.73, 8.54) | 55.57 | 20.28 (4.95, 54.41) | 19.32 (4.83, 54.55) | -0.15 (-0.16, -0.13) |
| Vanuatu | 0.01 (0, 0.02) | 0.02 (0, 0.05) | 220.98 | 22.86 (5.23, 64.5) | 23.09 (5.33, 64.74) | -0.01 (-0.03, 0.01) |
| Venezuela (Bolivarian Republic of) | 1.47 (0.36, 3.94) | 5.7 (1.43, 14.46) | 287.71 | 21.2 (5.32, 55.75) | 20.72 (5.12, 52.5) | -0.06 (-0.08, -0.03) |
| Viet Nam | 7.38 (1.76, 19.62) | 19.28 (4.68, 53.76) | 161.16 | 24.62 (5.91, 65.66) | 27.29 (6.67, 75.83) | 0.31 (0.27, 0.36) |
| Yemen | 0.61 (0.14, 1.67) | 1.93 (0.45, 5.53) | 217.82 | 27.41 (6.57, 74.87) | 26.09 (6.21, 74.58) | -0.17 (-0.19, -0.15) |
| Zambia | 0.29 (0.07, 0.81) | 0.77 (0.18, 2.21) | 161.94 | 21.96 (5.3, 62.38) | 24.58 (5.92, 67.83) | 0.46 (0.4, 0.52) |
| Zimbabwe | 0.49 (0.12, 1.35) | 0.67 (0.16, 1.94) | 35.81 | 24.81 (6.39, 65.56) | 23.11 (5.38, 63.68) | -0.16 (-0.25, -0.07) |

Note: CI, confidence interval; EAPC, estimated annual percentage change; GBD, Global Burden of Disease; UI, uncertainty interval


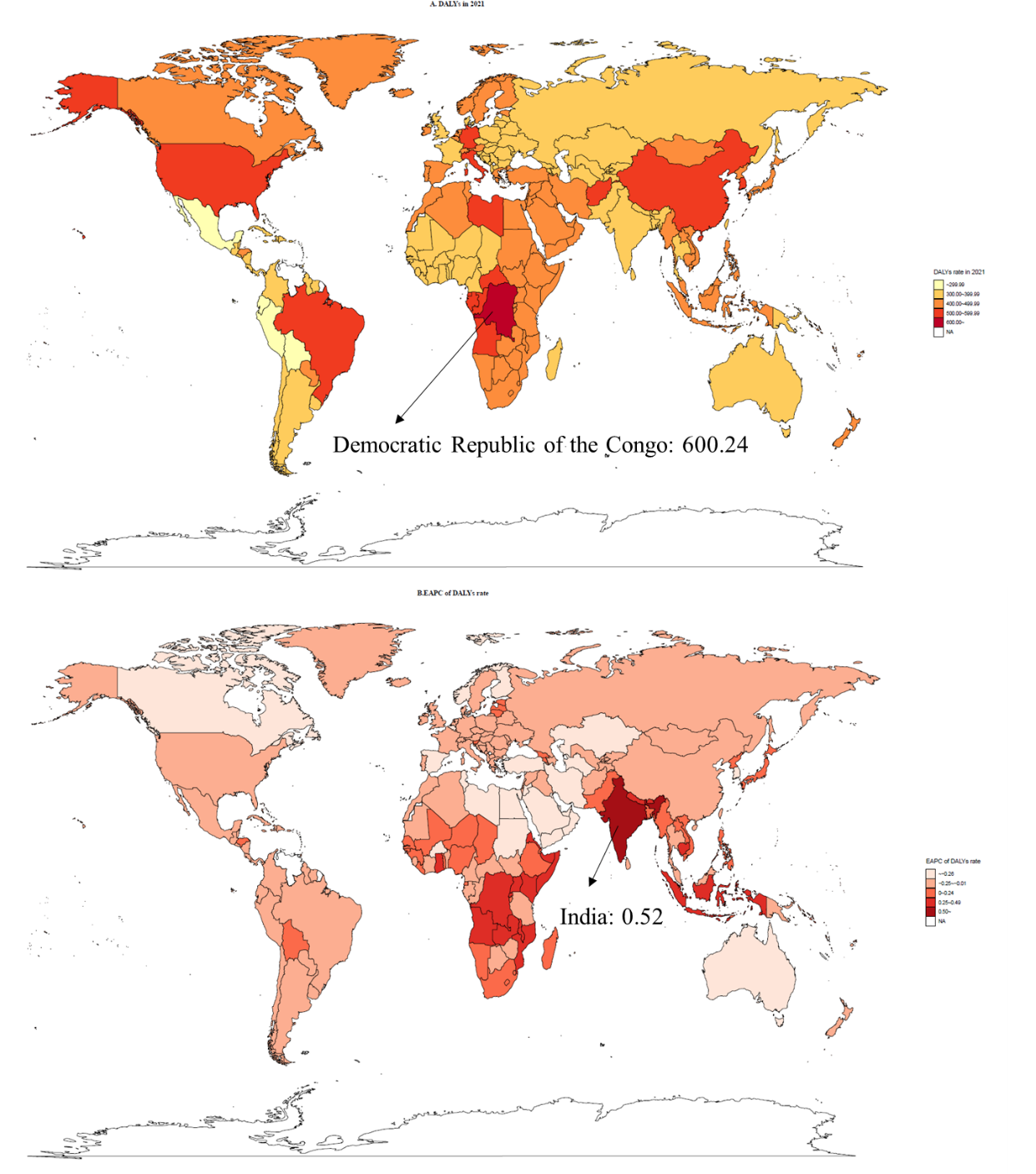


**Figure S5**. **The age-standardized DALYs rate for dementia and its temporal trend at national level, 1990−2021**

Note: DALYs: disability adjusted life years; EAPC, estimated annual percentage change

**Table S7**. **The DALYs and age-standardized DALYs rate for dementia and their temporal change at national level, 1990−2021**

|  | **DALYs (95% UI) per 1000 population** | | | **Age-standardized DALYs rate (95% UI), per 100, 000 population** | | |
| --- | --- | --- | --- | --- | --- | --- |
| **Countries and territories** | **1990** | **2021** | **Percentage change (%)** | **1990** | **2021** | **EAPC (95% CI)** |
| Afghanistan | 27.51 (12.14, 63.09) | 33.85 (15.2, 74.09) | 23.05 | 604.48 (259.91, 1383.43) | 577.72 (251.23, 1271.17) | -0.16 (-0.18, -0.14) |
| Albania | 6.4 (3.06, 14.14) | 16.19 (7.72, 34.86) | 153.07 | 402.99 (189.66, 887.25) | 391.82 (185.72, 836.06) | -0.08 (-0.1, -0.06) |
| Algeria | 38.63 (18.63, 88.17) | 124.68 (59.33, 268.17) | 222.77 | 510.58 (240.11, 1138.87) | 479.05 (224.05, 1029.3) | -0.19 (-0.21, -0.17) |
| American Samoa | 0.06 (0.03, 0.13) | 0.14 (0.07, 0.31) | 146.07 | 434.66 (193.98, 981.06) | 405.32 (188.48, 890.49) | -0.19 (-0.22, -0.15) |
| Andorra | 0.2 (0.1, 0.42) | 0.74 (0.35, 1.57) | 264.8 | 449.15 (213.89, 925.89) | 418.3 (197.77, 896.84) | -0.19 (-0.22, -0.16) |
| Angola | 10.1 (4.72, 22.38) | 35.83 (15.82, 84.58) | 254.66 | 511.9 (235.74, 1119.38) | 570.59 (247.01, 1310.57) | 0.34 (0.3, 0.38) |
| Antigua and Barbuda | 0.19 (0.09, 0.42) | 0.28 (0.13, 0.59) | 43.72 | 326.38 (156.48, 706.05) | 311.86 (148.11, 672.36) | -0.09 (-0.11, -0.06) |
| Argentina | 105.93 (52.09, 225.36) | 217.92 (103.87, 455.82) | 105.72 | 382.45 (185.41, 805.4) | 369.59 (176.33, 772.22) | -0.09 (-0.1, -0.08) |
| Armenia | 8.23 (4.04, 17.62) | 17.29 (8.32, 36.59) | 110.23 | 383.46 (186.79, 820.65) | 398.52 (189.72, 838.85) | 0.18 (0.14, 0.22) |
| Australia | 79.88 (38.81, 167.48) | 210.79 (97.74, 437.6) | 163.88 | 439.01 (211.17, 915.75) | 398.66 (187.36, 824.12) | -0.32 (-0.33, -0.31) |
| Austria | 55.67 (26.45, 120.34) | 98.13 (46.91, 203.13) | 76.27 | 448.76 (212.67, 960.88) | 425.92 (206.34, 877.24) | -0.16 (-0.17, -0.15) |
| Azerbaijan | 15.31 (7.5, 32.05) | 27.68 (13.24, 59.58) | 80.86 | 397.18 (188.19, 849.04) | 384.43 (179.15, 829.85) | -0.09 (-0.1, -0.08) |
| Bahamas | 0.4 (0.2, 0.87) | 1.04 (0.52, 2.2) | 157.29 | 322.24 (154.69, 691.36) | 312.32 (158.22, 666.95) | -0.09 (-0.11, -0.07) |
| Bahrain | 0.41 (0.2, 0.92) | 1.95 (0.96, 4.13) | 376.5 | 520.17 (240.47, 1181.12) | 464.81 (218.72, 1032.86) | -0.4 (-0.44, -0.35) |
| Bangladesh | 103.09 (47.74, 230.18) | 342.32 (154.93, 804.24) | 232.06 | 291.82 (135.94, 647.43) | 310.82 (138.97, 735.74) | 0.15 (0.09, 0.22) |
| Barbados | 1 (0.49, 2.12) | 1.64 (0.79, 3.49) | 64.76 | 319.07 (153.82, 684.03) | 313.79 (151.07, 667.52) | -0.02 (-0.07, 0.04) |
| Belarus | 47.35 (22.97, 100.41) | 65.53 (31.23, 139.74) | 38.39 | 398.18 (190.13, 842.13) | 393.88 (187.68, 837.83) | -0.04 (-0.06, -0.03) |
| Belgium | 80.55 (38.52, 171.38) | 138.88 (66.45, 285.46) | 72.42 | 507.61 (240.88, 1079.06) | 457.28 (222.55, 938.37) | -0.34 (-0.38, -0.29) |
| Belize | 0.3 (0.15, 0.62) | 0.79 (0.38, 1.66) | 164.58 | 330.6 (160.46, 686.06) | 321.18 (153.32, 672.41) | -0.04 (-0.08, 0) |
| Benin | 4.99 (2.29, 10.66) | 11.16 (5.06, 24.82) | 123.76 | 323.19 (146.11, 695.32) | 316.03 (141.36, 711.21) | -0.05 (-0.07, -0.02) |
| Bermuda | 0.18 (0.09, 0.38) | 0.51 (0.25, 1.05) | 183.62 | 333.74 (159.71, 708.87) | 322.9 (159.72, 664.5) | -0.12 (-0.14, -0.09) |
| Bhutan | 0.43 (0.2, 0.94) | 1.75 (0.77, 4.01) | 303.97 | 297.89 (137.34, 638.66) | 337.02 (145.62, 777.24) | 0.47 (0.43, 0.5) |
| Bolivia (Plurinational State of) | 6.64 (3.15, 14.28) | 20.52 (9.65, 44.55) | 209.04 | 293.85 (136.32, 635.66) | 294.23 (137.35, 638.66) | 0.02 (0, 0.04) |
| Bosnia and Herzegovina | 11.36 (5.5, 24.75) | 23.77 (11.55, 49.24) | 109.27 | 385.9 (182.97, 837.09) | 374.45 (182.06, 769.83) | -0.09 (-0.11, -0.08) |
| Botswana | 1.36 (0.63, 3.08) | 3.8 (1.76, 8.5) | 179.72 | 419.53 (189.14, 950.06) | 406.74 (186.63, 900.91) | -0.06 (-0.11, -0.01) |
| Brazil | 332.56 (155.59, 736.27) | 1201.68 (564.83, 2563.99) | 261.34 | 511.96 (234.64, 1114.18) | 503.99 (236.06, 1073.53) | -0.06 (-0.07, -0.05) |
| Brunei Darussalam | 0.26 (0.12, 0.57) | 0.78 (0.37, 1.67) | 201.48 | 386.48 (177.85, 839.95) | 396.91 (186.35, 850.7) | 0.17 (0.11, 0.24) |
| Bulgaria | 34.56 (17.16, 76.72) | 56.46 (27.4, 118.98) | 63.34 | 384.01 (179.87, 832.76) | 381 (182.04, 808.36) | -0.05 (-0.07, -0.02) |
| Burkina Faso | 10.15 (4.56, 22.8) | 20.98 (9.37, 47.86) | 106.66 | 374.24 (161.1, 852.32) | 341.84 (149.95, 776.62) | -0.34 (-0.39, -0.29) |
| Burundi | 6.27 (2.95, 14.04) | 12.34 (5.58, 28.64) | 96.87 | 412.68 (194.13, 912.37) | 431.79 (188.83, 1002.56) | 0.19 (0.16, 0.21) |
| Cabo Verde | 0.72 (0.34, 1.55) | 1.27 (0.57, 2.84) | 76.87 | 303.76 (141.96, 652.48) | 309.03 (138.82, 694.39) | 0.02 (0, 0.04) |
| Cambodia | 11.69 (5.69, 24.52) | 38.45 (17.79, 89.73) | 229.01 | 412.5 (196.15, 901.03) | 463.5 (209.71, 1084.98) | 0.44 (0.41, 0.46) |
| Cameroon | 9.79 (4.35, 21.56) | 25.1 (11.37, 59.28) | 156.48 | 346.06 (148.3, 758.27) | 325.66 (145.07, 779.51) | -0.23 (-0.28, -0.17) |
| Canada | 142.38 (73.45, 282.91) | 345.63 (172.91, 685.5) | 142.76 | 450.78 (230.93, 891.1) | 417.19 (213, 829.97) | -0.35 (-0.4, -0.3) |
| Central African Republic | 3.15 (1.44, 6.92) | 5.72 (2.54, 12.41) | 81.29 | 575.66 (257.05, 1267.47) | 567 (253.46, 1228.24) | -0.08 (-0.11, -0.05) |
| Chad | 6.5 (3.06, 14.01) | 11.4 (5.11, 25.94) | 75.37 | 312.02 (144.48, 689.01) | 311.23 (139.6, 709.81) | 0.01 (0, 0.03) |
| Chile | 30.26 (14.87, 64.03) | 96.15 (46.74, 199.85) | 217.69 | 366.35 (176.43, 772.1) | 361.94 (175.97, 753.94) | -0.04 (-0.05, -0.03) |
| China | 2702.48 (1239.18, 6085.39) | 10072.48 (4947.15, 22219.15) | 272.71 | 534.47 (236.2, 1190.6) | 562.39 (271.16, 1238.81) | -0.02 (-0.06, 0.02) |
| Colombia | 49.01 (24.25, 104.2) | 209.34 (102.59, 422.36) | 327.1 | 359.12 (175.51, 757.3) | 358.92 (176.4, 727.46) | -0.01 (-0.02, 0) |
| Comoros | 0.51 (0.24, 1.1) | 1.64 (0.72, 3.79) | 223.26 | 436.6 (198.84, 948.69) | 472.86 (202.81, 1099.2) | 0.29 (0.27, 0.31) |
| Congo | 3.28 (1.48, 7.22) | 8.33 (3.71, 18.68) | 154.08 | 585.15 (253.34, 1290.63) | 572.08 (242.82, 1281.13) | -0.06 (-0.08, -0.04) |
| Cook Islands | 0.04 (0.02, 0.09) | 0.1 (0.05, 0.21) | 151.29 | 446 (204.64, 1012.26) | 397.17 (185.81, 853.89) | -0.34 (-0.37, -0.32) |
| Costa Rica | 5.71 (2.81, 11.99) | 20.33 (9.95, 41.24) | 255.9 | 364.72 (178.33, 763.04) | 356.8 (176.36, 722.91) | -0.07 (-0.08, -0.05) |
| Cote d'Ivoire | 6.76 (3.06, 14.99) | 21.2 (9.51, 48.65) | 213.48 | 327.53 (145.45, 731.7) | 323.54 (141.27, 745.74) | -0.03 (-0.05, -0.01) |
| Croatia | 19.85 (9.64, 43.58) | 38.48 (18.39, 82.41) | 93.91 | 396.94 (190.69, 870.52) | 382.75 (182.83, 812.48) | -0.11 (-0.13, -0.09) |
| Cuba | 28.53 (14.18, 61.68) | 65.39 (31.39, 140.33) | 129.18 | 306.14 (147.67, 658.55) | 302.91 (147.14, 645.56) | -0.13 (-0.16, -0.11) |
| Cyprus | 2.79 (1.3, 6.51) | 8.47 (4.03, 18.25) | 203.32 | 494.58 (222.59, 1135.64) | 441.38 (204.91, 956.95) | -0.37 (-0.41, -0.32) |
| Czechia | 49.24 (23.99, 109.57) | 90.83 (43.92, 188.46) | 84.47 | 385.05 (181.59, 850.37) | 382.68 (184.26, 794.05) | -0.01 (-0.02, 0) |
| Democratic People's Republic of Korea | 43.34 (20.29, 97.05) | 117.03 (54.01, 258.18) | 170.01 | 422.86 (194.83, 954.64) | 431.35 (197.14, 950.17) | 0.12 (0.09, 0.15) |
| Democratic Republic of the Congo | 43.18 (19.35, 95.57) | 122.99 (53.71, 287.62) | 184.83 | 530.87 (234.29, 1153.36) | 600.24 (256.06, 1393.32) | 0.46 (0.42, 0.5) |
| Denmark | 35.86 (16.54, 77.86) | 53.95 (23.48, 117.13) | 50.44 | 402.72 (185.09, 869.35) | 385.44 (169.23, 831.45) | -0.16 (-0.22, -0.1) |
| Djibouti | 0.3 (0.14, 0.68) | 1.58 (0.71, 3.56) | 421.67 | 457.14 (205.06, 1006.6) | 481.94 (209.69, 1098.25) | 0.18 (0.15, 0.2) |
| Dominica | 0.18 (0.09, 0.4) | 0.24 (0.11, 0.49) | 30.3 | 329.64 (155.79, 712.6) | 321.07 (152.68, 665.17) | -0.07 (-0.08, -0.05) |
| Dominican Republic | 9.34 (4.67, 19.62) | 30.69 (14.86, 62.36) | 228.5 | 332.28 (160.12, 721.37) | 324.75 (156.9, 659.73) | -0.07 (-0.1, -0.04) |
| Ecuador | 12.18 (5.9, 26.51) | 40.48 (19.48, 86.46) | 232.23 | 280.71 (132.75, 606.44) | 270.88 (129.2, 583.75) | -0.09 (-0.11, -0.07) |
| Egypt | 73.95 (35.73, 167.55) | 159.72 (76.42, 354.13) | 115.99 | 491.36 (225.85, 1097.7) | 451.66 (212.99, 989.38) | -0.29 (-0.31, -0.27) |
| El Salvador | 10.12 (4.99, 21.54) | 25.53 (12.56, 52.15) | 152.2 | 365.91 (180.64, 780.61) | 361.86 (179.07, 738.85) | -0.03 (-0.04, -0.02) |
| Equatorial Guinea | 0.58 (0.27, 1.3) | 1.69 (0.75, 3.85) | 193.62 | 530.02 (238.47, 1152.8) | 562.36 (244.05, 1253.71) | 0.2 (0.15, 0.26) |
| Eritrea | 2.06 (0.97, 4.58) | 7 (3.02, 16.29) | 240.55 | 436.83 (201.55, 978.38) | 499.44 (210.46, 1155.36) | 0.44 (0.39, 0.49) |
| Estonia | 7.42 (3.65, 16.1) | 13.59 (6.54, 28.63) | 83.03 | 392.77 (190.1, 850.86) | 402.96 (193, 850.91) | 0.1 (0.06, 0.13) |
| Eswatini | 0.73 (0.33, 1.6) | 1.33 (0.6, 3.07) | 82.47 | 419.59 (187.58, 922.54) | 420.94 (191.51, 955.49) | 0.1 (0.07, 0.12) |
| Ethiopia | 48.78 (21.92, 112.65) | 146.6 (63.67, 332.07) | 200.55 | 472.01 (207.19, 1059.78) | 482.27 (208.72, 1084.59) | 0.08 (0.02, 0.15) |
| Fiji | 0.85 (0.4, 1.89) | 1.86 (0.89, 3.98) | 118.84 | 425.25 (192.76, 954.02) | 404.94 (191.74, 883.5) | -0.2 (-0.22, -0.18) |
| Finland | 33.85 (15.7, 74.49) | 71.28 (32.99, 149.13) | 110.58 | 476.52 (222.43, 1036.19) | 438.11 (204.07, 904.23) | -0.25 (-0.28, -0.23) |
| France | 352.24 (162.11, 767.03) | 699.42 (319.08, 1483.22) | 98.56 | 388.02 (176.76, 835.11) | 366.86 (171.08, 763.86) | -0.18 (-0.2, -0.17) |
| Gabon | 2.41 (1.08, 5.21) | 3.84 (1.72, 8.81) | 59.15 | 592.49 (256.17, 1284.17) | 588.29 (255.87, 1336.53) | -0.02 (-0.03, -0.01) |
| Gambia | 0.72 (0.33, 1.59) | 2.31 (1.01, 5.35) | 222.43 | 341.3 (154.14, 743.82) | 341.38 (142.91, 788.35) | -0.07 (-0.12, -0.02) |
| Georgia | 21.14 (10.18, 45.41) | 27.73 (13.46, 57.38) | 31.18 | 391.78 (185.7, 839.12) | 398.83 (191.96, 827.23) | 0.06 (0.02, 0.11) |
| Germany | 674.61 (328.26, 1415.81) | 1288.75 (625.38, 2595.51) | 91.04 | 510.92 (245.56, 1063.38) | 508.64 (250.83, 1028.42) | -0.05 (-0.08, -0.02) |
| Ghana | 11.18 (5.16, 25.85) | 34.03 (15.24, 80.4) | 204.36 | 297.5 (135.33, 682.06) | 327.77 (144.37, 776.72) | 0.33 (0.29, 0.38) |
| Greece | 62.96 (30.27, 135.84) | 143.59 (68.76, 305.89) | 128.06 | 442.61 (209.8, 948.67) | 429.55 (206.36, 907.38) | -0.1 (-0.11, -0.09) |
| Greenland | 0.09 (0.04, 0.19) | 0.22 (0.11, 0.44) | 144.07 | 485.34 (233.26, 1051.99) | 470.67 (229.5, 964.22) | -0.08 (-0.1, -0.05) |
| Grenada | 0.29 (0.14, 0.62) | 0.28 (0.14, 0.62) | -1.95 | 337.48 (163.41, 723.45) | 326.57 (158.38, 707.55) | 0.08 (0.04, 0.13) |
| Guam | 0.18 (0.09, 0.41) | 0.86 (0.43, 1.64) | 369.76 | 421.56 (191.4, 927.54) | 368.22 (185.54, 718.58) | -0.43 (-0.47, -0.39) |
| Guatemala | 8.12 (4.05, 17.68) | 33.39 (16.32, 68.47) | 311.02 | 369.03 (176.8, 798.15) | 360.29 (173.83, 750.81) | -0.09 (-0.1, -0.07) |
| Guinea | 7.71 (3.48, 16.63) | 13.02 (5.92, 29.99) | 68.77 | 310.3 (137.96, 669.31) | 321.03 (144.1, 737.33) | 0.12 (0.09, 0.15) |
| Guinea-Bissau | 0.74 (0.33, 1.68) | 1.29 (0.57, 3.01) | 74.01 | 329.06 (142.99, 735.28) | 338.39 (143.13, 800.84) | 0.1 (0.09, 0.12) |
| Guyana | 0.85 (0.42, 1.78) | 1.46 (0.73, 3.14) | 71.54 | 303.85 (145.81, 649.61) | 307.66 (149.8, 659.64) | 0 (-0.07, 0.08) |
| Haiti | 6.71 (3.32, 14.86) | 14.56 (7.03, 33.16) | 117.15 | 340.6 (164.07, 747.26) | 322.24 (153.66, 721.17) | -0.17 (-0.18, -0.16) |
| Honduras | 6 (2.87, 12.86) | 20.08 (9.34, 45.47) | 234.87 | 396.47 (190.93, 839.37) | 427.72 (199.15, 959.38) | 0.24 (0.19, 0.28) |
| Hungary | 50.2 (24.42, 111.16) | 84.26 (40.65, 173.85) | 67.87 | 385.8 (181.75, 842.77) | 380.96 (184.01, 785.57) | -0.01 (-0.02, 0.01) |
| Iceland | 1.42 (0.7, 2.95) | 2.98 (1.46, 6.07) | 110.22 | 454.55 (223.51, 942.86) | 429.58 (210.02, 870.83) | -0.16 (-0.19, -0.13) |
| India | 760.23 (352.48, 1776.41) | 2787.34 (1236.93, 6211.03) | 266.64 | 262.07 (122.6, 600.9) | 305.73 (135.42, 676.67) | 0.52 (0.48, 0.55) |
| Indonesia | 232.71 (116.39, 493.92) | 633.9 (302.56, 1387.13) | 172.4 | 365.07 (178.21, 779.12) | 422 (194.37, 933.56) | 0.41 (0.35, 0.47) |
| Iran (Islamic Republic of) | 78.65 (37.48, 171.63) | 300.43 (144.42, 638.66) | 281.97 | 506.44 (241.22, 1099.44) | 473.64 (225.81, 1003.84) | -0.25 (-0.26, -0.23) |
| Iraq | 33.79 (15.94, 72.41) | 72.8 (34.77, 156.09) | 115.46 | 490.69 (231.46, 1051.37) | 473.14 (223.03, 1007.5) | -0.2 (-0.23, -0.17) |
| Ireland | 16.74 (8.08, 36.52) | 36.03 (17.32, 74.45) | 115.24 | 444.18 (210.96, 962.81) | 417.27 (199.94, 861.14) | -0.17 (-0.19, -0.15) |
| Israel | 18.98 (9.18, 41.78) | 59.49 (28.62, 124.67) | 213.37 | 433.13 (204.21, 934.2) | 421.5 (202.2, 878.99) | -0.09 (-0.12, -0.07) |
| Italy | 450.86 (205.65, 977.09) | 1008.2 (479.89, 2046.52) | 123.62 | 516.62 (233.92, 1110.05) | 507.4 (243.87, 1036.59) | -0.03 (-0.06, -0.01) |
| Jamaica | 6.37 (3.18, 13.18) | 11.7 (5.78, 24.32) | 83.78 | 340.78 (169.9, 704.29) | 328.53 (164.6, 683.23) | -0.14 (-0.17, -0.11) |
| Japan | 688.56 (323.2, 1492.47) | 2539.29 (1209.73, 5046.91) | 268.78 | 460.57 (214.58, 985.33) | 456 (221.79, 920.17) | 0.03 (0.01, 0.06) |
| Jordan | 4.06 (2, 8.59) | 21.64 (10.92, 46.23) | 433.04 | 487.44 (236.71, 1035.26) | 450.02 (219.99, 960.28) | -0.29 (-0.31, -0.26) |
| Kazakhstan | 39.05 (18.99, 84.77) | 48.38 (23.94, 105.36) | 23.89 | 390.89 (184.64, 843.79) | 371.56 (177.75, 812.03) | -0.25 (-0.3, -0.21) |
| Kenya | 23.81 (11.02, 53.23) | 66.9 (29.26, 147.2) | 180.95 | 424.09 (190.83, 967.98) | 480.57 (205.49, 1052.02) | 0.46 (0.44, 0.47) |
| Kiribati | 0.09 (0.04, 0.21) | 0.19 (0.09, 0.41) | 101.73 | 444.73 (206.23, 1011.07) | 484.2 (221.09, 1072.53) | 0.27 (0.24, 0.31) |
| Kuwait | 1.8 (0.88, 3.79) | 9.29 (4.6, 18.99) | 415.38 | 504.17 (243.96, 1060.09) | 457.75 (224.95, 949.83) | -0.33 (-0.4, -0.26) |
| Kyrgyzstan | 9.75 (4.79, 20.63) | 13.6 (6.55, 27.88) | 39.52 | 396.12 (189.2, 837.35) | 386.99 (186.91, 800) | -0.08 (-0.1, -0.07) |
| Lao People's Democratic Republic | 4.77 (2.34, 10.48) | 12.59 (5.97, 27.89) | 163.81 | 389.09 (188.34, 847.42) | 407.47 (187.6, 907.38) | 0.19 (0.16, 0.21) |
| Latvia | 13.36 (6.42, 28.68) | 19.07 (9.16, 40.17) | 42.68 | 392.89 (186.13, 839.82) | 392.16 (187.44, 818.04) | 0.02 (-0.01, 0.05) |
| Lebanon | 7.59 (3.69, 15.75) | 30.71 (15.23, 63.47) | 304.64 | 473.64 (232.18, 975.21) | 460.13 (229.84, 949.69) | -0.07 (-0.14, 0.01) |
| Lesotho | 2.59 (1.22, 5.4) | 2.86 (1.31, 6.6) | 10.25 | 413.57 (194.21, 873.14) | 430.58 (193.02, 983.97) | 0.25 (0.18, 0.32) |
| Liberia | 2.42 (1.1, 5.71) | 4.09 (1.87, 9.51) | 69.23 | 308.34 (138.41, 703.75) | 307.31 (137.6, 719.91) | 0.03 (0, 0.06) |
| Libya | 8.52 (3.96, 17.6) | 19.17 (9.21, 42.78) | 125.08 | 550.6 (256.35, 1138.12) | 502.17 (241.77, 1123.68) | -0.26 (-0.29, -0.24) |
| Lithuania | 17.14 (8.17, 36.55) | 27.74 (13.34, 59.56) | 61.88 | 391.67 (185.38, 838.74) | 393.44 (190.01, 837.06) | 0.01 (-0.01, 0.04) |
| Luxembourg | 1.71 (0.84, 3.64) | 3.8 (1.76, 8) | 122.56 | 325.91 (156.55, 696.57) | 308.22 (144.66, 646.56) | -0.17 (-0.2, -0.15) |
| Madagascar | 11.99 (5.88, 26.26) | 21.67 (10.49, 47.29) | 80.81 | 360.43 (172.97, 789.78) | 365.63 (170.82, 805.91) | 0.06 (0.05, 0.08) |
| Malawi | 9.54 (4.5, 21.59) | 20.79 (9.39, 46.45) | 117.94 | 422.24 (194.25, 958.33) | 463.75 (206.32, 1030.55) | 0.34 (0.31, 0.37) |
| Malaysia | 33.86 (16.25, 72.59) | 98.75 (45.3, 216.71) | 191.67 | 445.1 (211.57, 957.71) | 445.34 (203.45, 976.51) | -0.05 (-0.09, 0) |
| Maldives | 0.19 (0.09, 0.42) | 1 (0.49, 2.09) | 430.66 | 362.78 (172.44, 801.4) | 384.86 (186.59, 802.36) | 0.18 (0.14, 0.22) |
| Mali | 8.52 (3.72, 19.05) | 19.55 (8.45, 45.89) | 129.59 | 366.7 (156.49, 838.12) | 363.61 (153.57, 846.85) | 0.01 (-0.02, 0.04) |
| Malta | 1.65 (0.79, 3.51) | 4.91 (2.4, 10.19) | 198.02 | 442.14 (207.61, 934.7) | 421.98 (204.82, 872.32) | -0.19 (-0.22, -0.17) |
| Marshall Islands | 0.04 (0.02, 0.1) | 0.07 (0.03, 0.17) | 66.81 | 450.32 (202.99, 1044.26) | 420.98 (191.27, 996.32) | -0.24 (-0.25, -0.23) |
| Mauritania | 2.3 (1.05, 5.18) | 5.29 (2.37, 12.77) | 129.62 | 331.16 (148.76, 747.32) | 329.08 (146.78, 795.2) | -0.07 (-0.09, -0.05) |
| Mauritius | 2.08 (1, 4.59) | 6.52 (3.15, 13.91) | 213.75 | 410.23 (193.18, 896.71) | 390.08 (186.92, 832.38) | -0.18 (-0.25, -0.12) |
| Mexico | 100.58 (50.17, 215.76) | 331.08 (163.37, 717.52) | 229.18 | 309.4 (150.68, 660.7) | 296.84 (144.66, 642.67) | -0.11 (-0.12, -0.1) |
| Micronesia (Federated States of) | 0.16 (0.07, 0.37) | 0.2 (0.09, 0.45) | 27.62 | 476.07 (218.92, 1100.61) | 468.56 (217.61, 1082.02) | -0.04 (-0.07, 0) |
| Monaco | 0.39 (0.18, 0.85) | 0.58 (0.27, 1.17) | 49.35 | 453.72 (215.99, 1003.31) | 450.66 (212.6, 919.67) | -0.01 (-0.03, 0.01) |
| Mongolia | 3.14 (1.49, 6.54) | 5.7 (2.78, 12.46) | 81.12 | 412.86 (193.85, 881.39) | 402.27 (192.91, 881.94) | -0.15 (-0.18, -0.13) |
| Montenegro | 2.18 (1.04, 4.67) | 3.26 (1.56, 6.97) | 49.61 | 392.24 (185.5, 838.01) | 383.69 (179.33, 823.48) | -0.09 (-0.11, -0.06) |
| Morocco | 55.38 (27.16, 120.17) | 127.78 (59.7, 281.25) | 130.72 | 498.06 (241.3, 1079.72) | 479.67 (223.95, 1061.79) | -0.09 (-0.11, -0.07) |
| Mozambique | 15.97 (7.35, 35.81) | 30.65 (13.27, 71.35) | 91.9 | 456.75 (201.73, 1027.76) | 490.46 (207.92, 1152.97) | 0.35 (0.31, 0.38) |
| Myanmar | 58.09 (29.18, 124.56) | 149.83 (70.32, 330.24) | 157.94 | 390.49 (192.75, 822.66) | 407.47 (187.74, 911.87) | 0.12 (0.09, 0.14) |
| Namibia | 1.56 (0.73, 3.41) | 3.88 (1.76, 9.22) | 149.2 | 402.65 (187.31, 907.77) | 432.91 (196.45, 1035.86) | 0.24 (0.22, 0.26) |
| Nauru | 0.01 (0, 0.02) | 0.01 (0.01, 0.03) | 40.01 | 447.11 (201.16, 1005.34) | 448.4 (201.57, 1042.39) | 0.03 (0, 0.05) |
| Nepal | 16.48 (8.14, 34.5) | 53.24 (24.52, 119.23) | 223.05 | 280.91 (139.12, 594.87) | 310.85 (140.5, 699.68) | 0.34 (0.3, 0.39) |
| Netherlands | 99.01 (47.15, 208.67) | 192.57 (90.11, 397.97) | 94.5 | 482.11 (228.55, 1010.99) | 474.98 (223.6, 983.43) | -0.03 (-0.04, -0.02) |
| New Zealand | 17.27 (8.26, 36.98) | 41.39 (19.7, 85.37) | 139.71 | 465.69 (219.62, 984.68) | 441.17 (210.93, 909.28) | -0.21 (-0.25, -0.17) |
| Nicaragua | 4.41 (2.22, 9.15) | 14.68 (7.5, 30.01) | 232.5 | 365.48 (181.44, 755.05) | 356.84 (181.81, 732.29) | -0.05 (-0.07, -0.02) |
| Niger | 5.07 (2.31, 11) | 16.3 (7.39, 36.65) | 221.34 | 330.91 (147.98, 724.22) | 331.02 (147.57, 734.6) | 0.07 (0.03, 0.11) |
| Nigeria | 89.84 (41.32, 200.5) | 186.09 (80.14, 431.73) | 107.14 | 296.84 (131.5, 656.93) | 309.45 (131.77, 715.01) | 0.22 (0.16, 0.28) |
| Niue | 0.01 (0.01, 0.03) | 0.01 (0, 0.02) | -28.55 | 478.71 (213.11, 1086.49) | 434.13 (196.03, 954.18) | -0.33 (-0.35, -0.32) |
| North Macedonia | 5.7 (2.78, 12.22) | 9.17 (4.47, 19.5) | 60.78 | 371.22 (179.98, 794.46) | 373.95 (177.2, 816.33) | -0.01 (-0.03, 0.02) |
| Northern Mariana Islands | 0.03 (0.02, 0.07) | 0.13 (0.06, 0.29) | 310.22 | 420.69 (193.45, 939.21) | 410.96 (189.74, 911.58) | -0.05 (-0.09, -0.01) |
| Norway | 37.08 (17.73, 79.03) | 51.75 (24.04, 108.68) | 39.56 | 477.46 (227.82, 1011.83) | 427.76 (202.41, 901.9) | -0.38 (-0.41, -0.35) |
| Oman | 2.2 (1.06, 4.7) | 5.23 (2.53, 11.95) | 137.6 | 512.85 (245.4, 1088.66) | 472.28 (223.68, 1076.18) | -0.26 (-0.35, -0.17) |
| Pakistan | 129.2 (58.72, 291.18) | 265.49 (113.13, 614.29) | 105.49 | 310.92 (138.64, 710.19) | 333.84 (140.09, 758.92) | 0.16 (0.13, 0.2) |
| Palau | 0.03 (0.01, 0.06) | 0.05 (0.03, 0.12) | 91.49 | 435.69 (194.62, 990.99) | 389.68 (175.49, 883.59) | -0.33 (-0.37, -0.3) |
| Palestine | 3.22 (1.54, 7.17) | 7.5 (3.7, 16.35) | 132.64 | 494.79 (232.85, 1111.89) | 461.77 (223.02, 1011.99) | -0.23 (-0.29, -0.18) |
| Panama | 4.58 (2.28, 9.77) | 16.2 (7.99, 32.47) | 254.03 | 349.94 (172.43, 743.98) | 348.86 (174.09, 698.83) | 0 (-0.02, 0.02) |
| Papua New Guinea | 3.82 (1.83, 8.36) | 11.06 (5.31, 24.71) | 189.68 | 413.72 (196.86, 911.19) | 393.4 (182.94, 868.87) | -0.21 (-0.24, -0.19) |
| Paraguay | 9.02 (4.31, 19.01) | 24.54 (11.31, 51.41) | 172.13 | 467.69 (223.43, 985.68) | 465.79 (215.8, 974.39) | 0 (-0.02, 0.01) |
| Peru | 28.49 (13.38, 62.17) | 90.02 (43.35, 191.86) | 216.01 | 274.95 (128.33, 602.68) | 268.5 (129.48, 574.76) | -0.14 (-0.17, -0.11) |
| Philippines | 76.34 (38, 164.18) | 245.43 (116.78, 509.6) | 221.48 | 393.94 (186.07, 844.77) | 410.29 (191, 846.37) | 0.15 (0.14, 0.17) |
| Poland | 156.21 (75.21, 344.07) | 312.58 (149.06, 666.12) | 100.1 | 406.31 (192.93, 884.27) | 394.77 (187.59, 844.38) | -0.09 (-0.1, -0.08) |
| Portugal | 53.77 (25.62, 119.91) | 138.95 (66.14, 290.91) | 158.44 | 444.35 (208.98, 981.3) | 432.79 (205.33, 900.75) | -0.07 (-0.09, -0.05) |
| Puerto Rico | 11.13 (5.44, 24.06) | 30.27 (14.87, 62.17) | 171.86 | 332.94 (158.99, 712.73) | 318.47 (158.35, 648.17) | -0.16 (-0.17, -0.14) |
| Qatar | 0.23 (0.11, 0.51) | 1.73 (0.84, 3.83) | 649.91 | 518.27 (237.9, 1181) | 467.95 (215.8, 1029.22) | -0.31 (-0.36, -0.26) |
| Republic of Korea | 101.64 (47.1, 222.69) | 461.8 (218.82, 928.41) | 354.34 | 580.65 (260.79, 1256.03) | 500.05 (234.92, 1004.38) | -0.36 (-0.4, -0.31) |
| Republic of Moldova | 12.32 (5.91, 26.72) | 23.05 (11.5, 49.15) | 87.07 | 387.64 (183.52, 834.96) | 372.43 (187, 797.15) | -0.14 (-0.17, -0.11) |
| Romania | 83.05 (40.76, 184.47) | 160.36 (77.96, 338.94) | 93.09 | 382.93 (182.26, 838.65) | 382.75 (185.17, 803.55) | -0.02 (-0.03, -0.01) |
| Russian Federation | 589.96 (286.54, 1293.55) | 971.9 (459.55, 2086.55) | 64.74 | 406.03 (192.39, 878.44) | 397.87 (188.83, 852.89) | -0.08 (-0.1, -0.06) |
| Rwanda | 6.95 (3.26, 15.41) | 18.9 (8.4, 42.83) | 171.99 | 438.8 (199.65, 980.2) | 492.89 (213.52, 1114.28) | 0.41 (0.39, 0.43) |
| Saint Kitts and Nevis | 0.11 (0.05, 0.23) | 0.15 (0.07, 0.33) | 44.85 | 314.31 (149.41, 678.21) | 307.67 (147.14, 668.04) | 0.02 (-0.02, 0.06) |
| Saint Lucia | 0.24 (0.12, 0.52) | 0.73 (0.35, 1.54) | 207.83 | 325.28 (155.18, 704.03) | 318.21 (152.98, 674.9) | -0.14 (-0.16, -0.12) |
| Saint Vincent and the Grenadines | 0.21 (0.1, 0.45) | 0.4 (0.2, 0.85) | 87.44 | 343.72 (163.72, 733.64) | 313.61 (151.38, 678.48) | -0.22 (-0.27, -0.17) |
| Samoa | 0.28 (0.13, 0.64) | 0.49 (0.22, 1.13) | 74 | 479.55 (214.02, 1073.98) | 457.23 (202.49, 1060.53) | -0.15 (-0.19, -0.12) |
| San Marino | 0.16 (0.08, 0.33) | 0.41 (0.2, 0.83) | 153.33 | 426.84 (208.93, 870.41) | 384.39 (193.81, 772.22) | -0.28 (-0.32, -0.24) |
| Sao Tome and Principe | 0.16 (0.07, 0.37) | 0.23 (0.1, 0.53) | 45.4 | 311 (139.5, 704.54) | 305.55 (136.27, 683.23) | -0.05 (-0.09, 0) |
| Saudi Arabia | 19.12 (8.99, 40.2) | 40.84 (19.15, 87.87) | 113.55 | 501.05 (232.4, 1064.97) | 456.71 (208.96, 1019.77) | -0.33 (-0.35, -0.31) |
| Senegal | 7.29 (3.32, 16.34) | 19.2 (8.36, 44.63) | 163.26 | 330.44 (147.37, 740.71) | 349.47 (149.3, 822.3) | 0.17 (0.14, 0.19) |
| Serbia | 31.71 (15.24, 69.05) | 66.36 (31.7, 142.06) | 109.25 | 383.3 (178.08, 837.65) | 378.95 (180.18, 812.59) | -0.1 (-0.13, -0.07) |
| Seychelles | 0.23 (0.11, 0.5) | 0.37 (0.18, 0.8) | 61.39 | 417.52 (195.33, 908.01) | 390.79 (185.16, 847.26) | -0.2 (-0.22, -0.18) |
| Sierra Leone | 4.59 (2.12, 10.3) | 7.75 (3.56, 17.8) | 68.59 | 304.5 (140.12, 675.61) | 303.05 (134.33, 690.1) | 0 (-0.02, 0.02) |
| Singapore | 5.25 (2.54, 11.36) | 26.86 (13.12, 54.78) | 411.47 | 334.82 (160.51, 718.72) | 327.69 (160.06, 667.67) | -0.01 (-0.03, 0.02) |
| Slovakia | 21.3 (10.31, 46.09) | 36.92 (17.76, 77.05) | 73.28 | 392.5 (186.93, 840.91) | 386.66 (186.37, 808.1) | -0.02 (-0.04, 0) |
| Slovenia | 8.98 (4.23, 19.75) | 20.97 (10.18, 42.89) | 133.44 | 382 (178.71, 834.83) | 385.66 (186.57, 793.44) | 0.05 (0, 0.1) |
| Solomon Islands | 0.3 (0.14, 0.72) | 0.87 (0.41, 1.96) | 186.43 | 431.35 (195.74, 993.79) | 431.44 (198.77, 981.36) | 0.02 (-0.02, 0.05) |
| Somalia | 4.93 (2.31, 10.78) | 12.91 (5.87, 28.94) | 161.73 | 415.69 (191.62, 905.33) | 444.96 (198.6, 1030.23) | 0.34 (0.29, 0.38) |
| South Africa | 64.72 (31.06, 139.27) | 140.2 (65.09, 313.93) | 116.63 | 396.83 (186.63, 850.32) | 406.65 (185.95, 901.85) | 0.07 (0.01, 0.12) |
| South Sudan | 7.03 (3.23, 15.57) | 9.58 (4.29, 21.62) | 36.3 | 411.64 (184.29, 891.43) | 413.22 (178.87, 939.24) | 0 (-0.04, 0.05) |
| Spain | 245.88 (113.83, 515.37) | 547.72 (251.38, 1125.39) | 122.76 | 460.46 (211.09, 964.8) | 418.19 (195.24, 860.08) | -0.25 (-0.28, -0.22) |
| Sri Lanka | 28.17 (14.05, 61.1) | 84.9 (40.78, 173.23) | 201.38 | 374.41 (178.08, 808.5) | 369 (177.21, 764.88) | -0.04 (-0.06, -0.02) |
| Sudan | 29.96 (14.45, 69.69) | 60.19 (28.93, 127.73) | 100.91 | 478.34 (226.03, 1088.16) | 442.22 (209.16, 934.95) | -0.28 (-0.3, -0.26) |
| Suriname | 0.74 (0.37, 1.57) | 1.85 (0.93, 3.91) | 150.28 | 333.79 (166.14, 711.38) | 323.39 (161.32, 680.94) | -0.11 (-0.13, -0.08) |
| Sweden | 76.67 (37.24, 158.36) | 115.34 (55.53, 237.4) | 50.44 | 447.24 (215.79, 921.22) | 416.99 (202.33, 857.28) | -0.16 (-0.18, -0.13) |
| Switzerland | 52.15 (24.89, 111.06) | 99.78 (47.3, 203.58) | 91.35 | 449.64 (212.92, 955.62) | 425.52 (205.38, 877.7) | -0.19 (-0.26, -0.12) |
| Syrian Arab Republic | 18.72 (9.09, 40.38) | 40.57 (19.81, 87.23) | 116.65 | 482.32 (235.56, 1046.32) | 451.67 (219.33, 983.43) | -0.23 (-0.25, -0.21) |
| Taiwan (Province of China) | 35.82 (16.8, 80.33) | 169.63 (79.1, 360.77) | 373.6 | 350.75 (161.94, 766.05) | 372.14 (175.26, 790.04) | 0 (-0.1, 0.1) |
| Tajikistan | 8.92 (4.21, 19.27) | 14.54 (6.78, 31.22) | 63.11 | 392.87 (186.09, 849.45) | 391.81 (182.51, 858.86) | -0.03 (-0.05, 0) |
| Thailand | 103.41 (48.46, 221.45) | 436.13 (204.62, 922.68) | 321.74 | 405.1 (188.11, 872.98) | 395.44 (185.94, 837.14) | -0.15 (-0.19, -0.12) |
| Timor-Leste | 0.62 (0.31, 1.32) | 2.5 (1.2, 5.52) | 300.98 | 382.17 (185.81, 835.81) | 399.65 (185.73, 906.77) | 0.2 (0.16, 0.24) |
| Togo | 2.45 (1.12, 5.29) | 7.36 (3.25, 17.46) | 200.18 | 324.67 (143.62, 721.44) | 340.84 (148.6, 819.2) | 0.17 (0.14, 0.19) |
| Tokelau | 0.01 (0, 0.01) | 0.01 (0, 0.01) | 18.68 | 491.49 (217.21, 1100.53) | 446.5 (201.71, 992.48) | -0.33 (-0.33, -0.32) |
| Tonga | 0.18 (0.09, 0.42) | 0.33 (0.15, 0.72) | 78.7 | 466.65 (214.52, 1043.66) | 451.66 (205.62, 990.56) | -0.07 (-0.08, -0.06) |
| Trinidad and Tobago | 2.17 (1.09, 4.8) | 5.76 (2.81, 12.25) | 165.1 | 326.9 (158.27, 713.26) | 314.81 (154.46, 665.73) | -0.07 (-0.09, -0.05) |
| Tunisia | 17.79 (8.57, 38.94) | 54.87 (26.64, 115.64) | 208.54 | 527.4 (245.11, 1131.51) | 485.94 (234.03, 1019.04) | -0.27 (-0.28, -0.26) |
| Turkey | 141.13 (68.91, 297.64) | 401.91 (196.64, 841.83) | 184.77 | 551.48 (267.29, 1158.43) | 491.99 (236.53, 1031.11) | -0.4 (-0.47, -0.32) |
| Turkmenistan | 5.42 (2.62, 11.75) | 11.36 (5.51, 25.24) | 109.56 | 394.54 (187.86, 847.99) | 373.23 (181.83, 824.24) | -0.26 (-0.29, -0.23) |
| Tuvalu | 0.02 (0.01, 0.04) | 0.03 (0.02, 0.08) | 84.29 | 479.06 (219.38, 1112.09) | 456.66 (210.11, 1054.29) | -0.17 (-0.18, -0.17) |
| Uganda | 16.15 (7.64, 34.23) | 42.25 (19.16, 96.81) | 161.69 | 394.33 (180.61, 852.94) | 453.76 (201.58, 1038.69) | 0.49 (0.45, 0.52) |
| Ukraine | 246.61 (119.63, 538.88) | 317.86 (145.5, 694.75) | 28.89 | 402.57 (191.76, 867.85) | 396.3 (181.53, 863.58) | -0.06 (-0.08, -0.04) |
| United Arab Emirates | 1.04 (0.48, 2.3) | 5.91 (2.85, 13.11) | 470.06 | 507.16 (231.84, 1108.06) | 427.48 (198.64, 928.24) | -0.42 (-0.51, -0.33) |
| United Kingdom | 388.79 (183.32, 833.87) | 615.41 (288.56, 1286.18) | 58.29 | 409.43 (191.44, 870.23) | 390.4 (183.98, 817.67) | -0.1 (-0.13, -0.08) |
| United Republic of Tanzania | 30.16 (13.86, 67.59) | 77.34 (34.29, 175.05) | 156.39 | 448.87 (200.94, 973.23) | 441.03 (193.01, 993.62) | -0.06 (-0.08, -0.04) |
| United States of America | 1789.06 (842.34, 3821.14) | 3318.64 (1550.26, 6957.87) | 85.5 | 528.79 (248.85, 1122.13) | 509.74 (240.09, 1068.07) | -0.15 (-0.16, -0.13) |
| United States Virgin Islands | 0.2 (0.1, 0.42) | 0.55 (0.28, 1.14) | 177.48 | 337.77 (159.15, 712.1) | 302.56 (148.84, 624.93) | -0.34 (-0.35, -0.33) |
| Uruguay | 14.46 (6.94, 31.02) | 26.12 (12.4, 55.82) | 80.67 | 381.12 (181.52, 811.18) | 379.78 (180.21, 797.09) | -0.03 (-0.03, -0.02) |
| Uzbekistan | 38.26 (18.19, 81.47) | 65.99 (32.04, 141.97) | 72.48 | 378.52 (178.1, 809.14) | 367.07 (176.44, 801.55) | -0.09 (-0.1, -0.08) |
| Vanuatu | 0.14 (0.06, 0.32) | 0.42 (0.19, 0.96) | 202.8 | 414.9 (186.63, 961.38) | 414.82 (185.48, 949.4) | -0.04 (-0.06, -0.02) |
| Venezuela (Bolivarian Republic of) | 31.86 (15.76, 67.18) | 113.23 (56.09, 235.49) | 255.37 | 415.88 (202.04, 869.58) | 409.15 (203.98, 846.15) | -0.06 (-0.08, -0.05) |
| Viet Nam | 150.24 (70.42, 325.42) | 372.89 (167.94, 839.08) | 148.2 | 454.56 (211.42, 984.4) | 476.41 (214.33, 1068.35) | 0.13 (0.1, 0.16) |
| Yemen | 14.96 (7.27, 32) | 43.85 (21.31, 99.86) | 193.18 | 521.37 (246.81, 1115.43) | 486.21 (234.59, 1103.25) | -0.25 (-0.27, -0.22) |
| Zambia | 6.87 (3.24, 15.19) | 17.47 (8.02, 39.67) | 154.34 | 396.43 (182.19, 890.11) | 431.45 (196.36, 999.22) | 0.35 (0.31, 0.39) |
| Zimbabwe | 11.02 (5.12, 23.19) | 16.37 (7.4, 38.02) | 48.49 | 434.92 (201.17, 966.47) | 417.09 (191.94, 965.99) | -0.11 (-0.17, -0.05) |

Note: CI, confidence interval; DALYs: disability adjusted life years; EAPC, estimated annual percentage change; UI, uncertainty interval


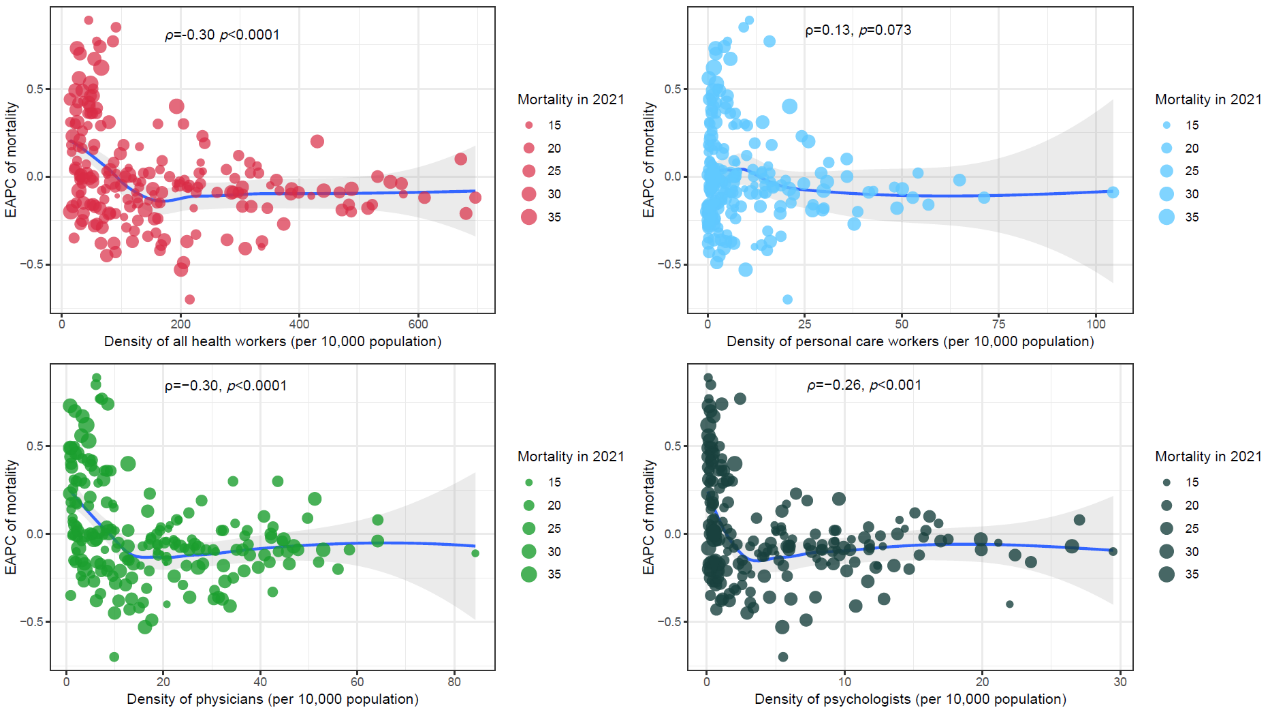


**Figure S6**. **The correlations between EAPC of age-standardized mortality rate and HRH.**

Notes: EAPC, estimated annual percentage change; HRH, human resources for health


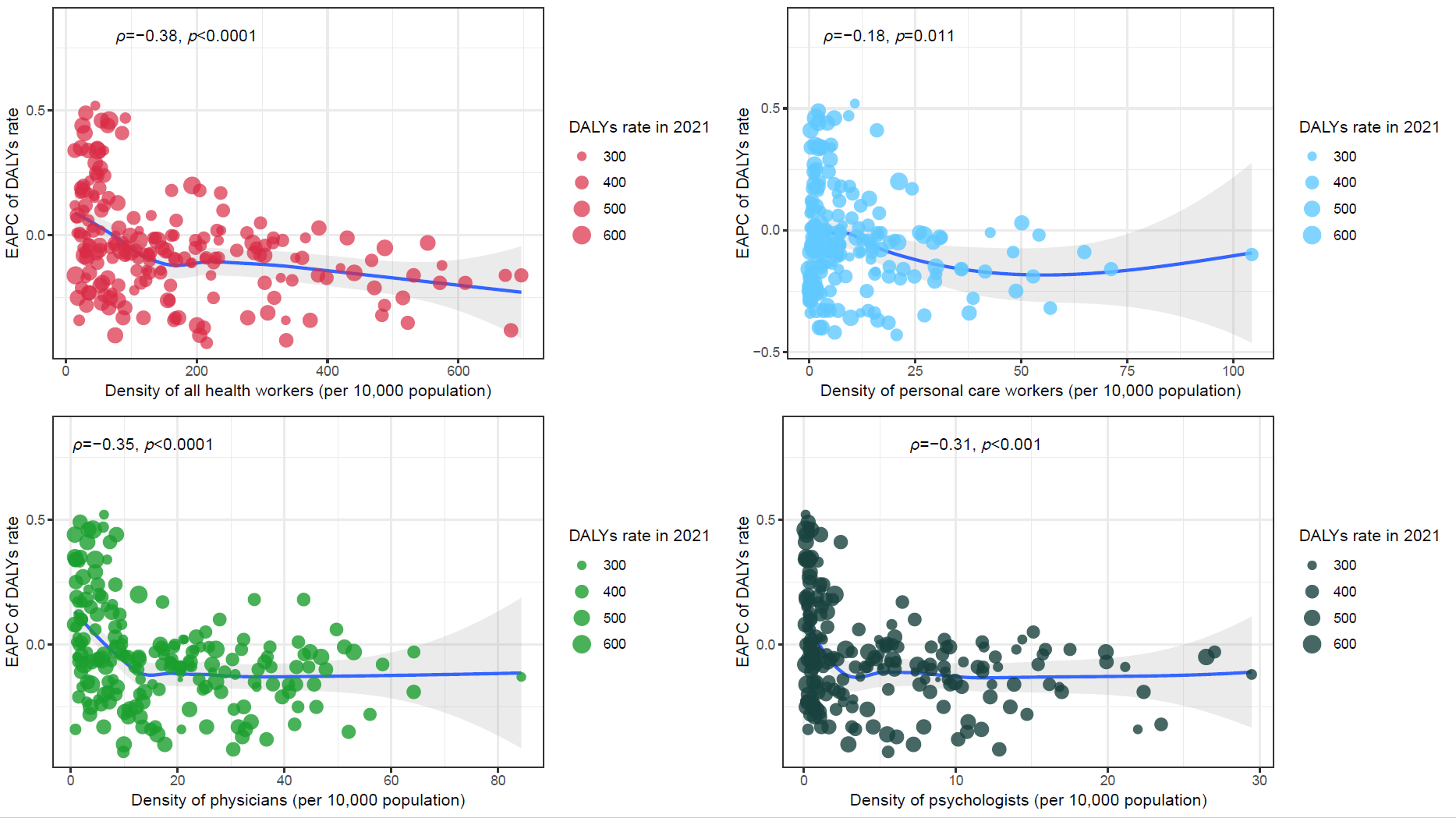


**Figure S7**. **The correlations between EAPC of age-standardized DALYs rate and HRH.**

Notes: DALYs: disability adjusted life years; EAPC, estimated annual percentage change; HRH, human resources for health


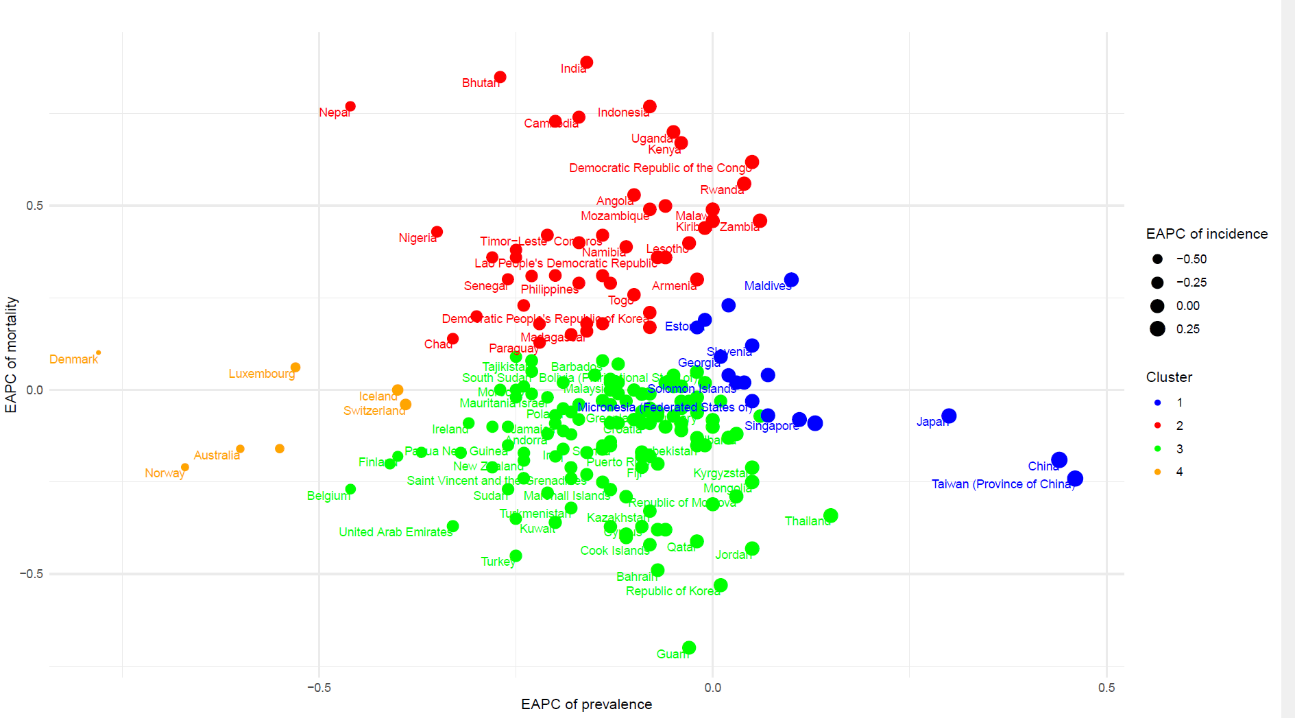


**Figure S8. A hierarchy cluster analysis in terms of temporal trends in diseases burden of dementia.**

Notes: EAPC, estimated annual percentage change


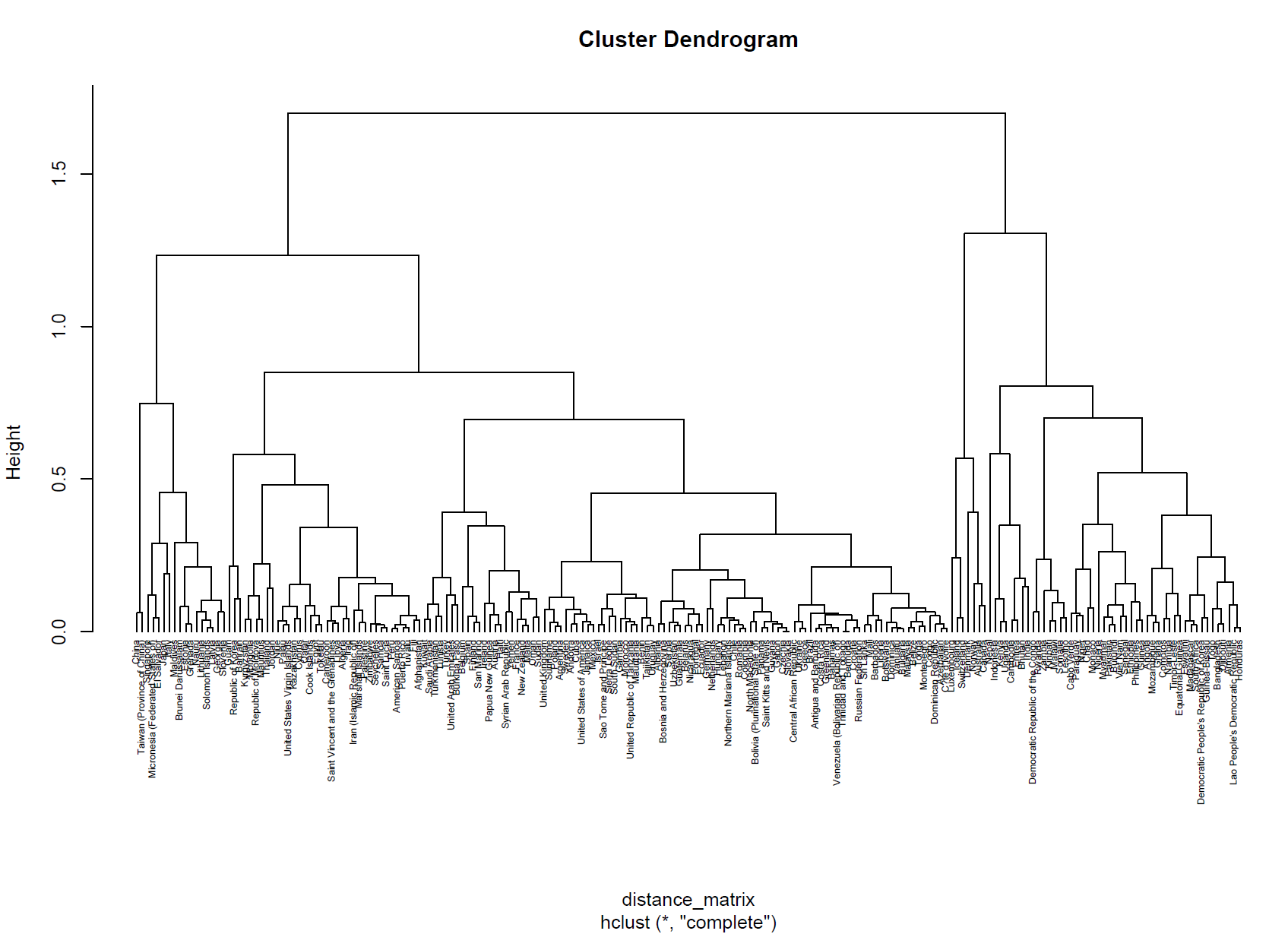


**Figure S9**. **The cluster dendrogram of hierarchy cluster analysis in terms of temporal trends in diseases burden of dementia.**

Notes: EAPC, estimated annual percentage change
